# Splice site m^6^A methylation prevents binding of U2AF35 to inhibit RNA splicing

**DOI:** 10.1016/j.cell.2021.03.062

**Published:** 2021-06-10

**Authors:** Mateusz Mendel, Kamila Delaney, Radha Raman Pandey, Kuan-Ming Chen, Joanna M. Wenda, Cathrine Broberg Vågbø, Florian A. Steiner, David Homolka, Ramesh S. Pillai

**Affiliations:** 1 Department of Molecular Biology, Science III, University of Geneva, 30 Quai Ernest-Ansermet, 1211 Geneva 4, Switzerland; 2 Proteomics and Modomics Experimental Core (PROMEC), Department of Clinical and Molecular Medicine, Norwegian University of Science and Technology (NTNU) and St. Olavs Hospital Central Staff, Trondheim, Norway

**Keywords:** METTL16, METT-10, m^6^A, 3' splice site, U6 snRNA, splicing, SAM synthetase, U2AF35/65, spermatogenesis, SAM homeostasis

## Abstract

The *N*^*6*^-methyladenosine (m^6^A) RNA modification is used widely to alter the fate of mRNAs. Here we demonstrate that the *C. elegans* writer METT-10 (the ortholog of mouse METTL16) deposits an m^6^A mark on the 3′ splice site (AG) of the S-adenosylmethionine (SAM) synthetase pre-mRNA, which inhibits its proper splicing and protein production. The mechanism is triggered by a rich diet and acts as an m^6^A-mediated switch to stop SAM production and regulate its homeostasis. Although the mammalian SAM synthetase pre-mRNA is not regulated via this mechanism, we show that splicing inhibition by 3′ splice site m^6^A is conserved in mammals. The modification functions by physically preventing the essential splicing factor U2AF35 from recognizing the 3′ splice site. We propose that use of splice-site m^6^A is an ancient mechanism for splicing regulation.

## Highlights

• m^6^A deposited at 3′ splice site by worm METT-10 inhibits splicing
• Methylation blocks 3′ splice site recognition by splicing factor U2AF35
• Methylation and splicing inhibition is a response to change in worm diet
• Splicing inhibition by 3′ splice site m^6^A is conserved in mammals

## Graphical abstract

**Figure:**
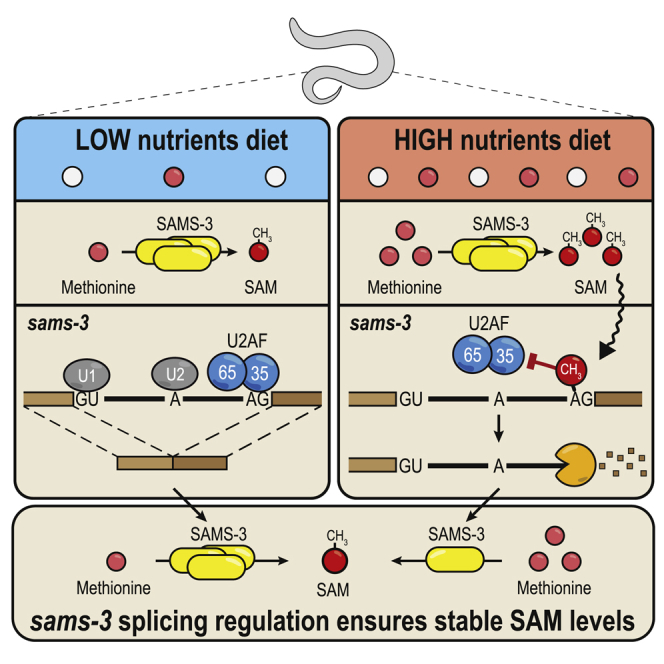

## Teaser

m^6^A methylation of a 3ʹ splice site blocks its recognition by splicing factors to inhibit pre-mRNA splicing in nematodes and mammals. In worms, this mechanism is used to modulate splicing in response to a change in diet.

## Introduction

It has been known since the early 1970s that RNAs can be modified with *N*^*6*^-methyladenosine (m^6^A) (Desrosiers et al., 1974, 1975; Schibler et al., 1977; Wei and Moss, 1977; Wei et al., 1975a, 1975b). It is the most abundant internal modification on eukaryotic mRNA (Fu et al., 2014; Patil et al., 2018; Roignant and Soller, 2017), with ∼4 m^6^A/10^4^ nucleotides (nt) detected in poly(A)^+^ RNA from adult mouse testes (Pandey et al., 2020). The mammalian heterodimeric METTL3/METTL14 RNA methyltransferase complex is the dominant m^6^A “writer,” with orthologs in organisms such as yeast, flies, and plants (Liu et al., 2014; Śledź and Jinek, 2016; Wang et al., 2016). The complex installs the m^6^A mark within a loosely defined RR**m**^**6**^**A**CH motif at thousands of sites in the transcriptome, with a bias towards the 3′ end of the RNA, where it is enriched near the stop codon (Dominissini et al., 2012; Kan et al., 2017; Meyer et al., 2012; Schwartz et al., 2013). m^6^A marks are recognized by various “reader” proteins, like those belonging to the YTH family (Patil et al., 2018), to modulate RNA splicing, stability, and translation (Li et al., 2014; Theler et al., 2014; Zhang et al., 2010). Gene regulation by this writer-reader system is essential for embryonic development in plants and mice (Batista et al., 2014; Geula et al., 2015; Kasowitz et al., 2018; Lasman et al., 2020; Zhong et al., 2008), mammalian fertility (Hsu et al., 2017; Ivanova et al., 2017; Jain et al., 2018; Wojtas et al., 2017), sex determination in flies (Haussmann et al., 2016; Lence et al., 2016), and many other developmental processes. Notably, this m^6^A writer-reader system is absent in nematodes.

The second mRNA m^6^A writer, METTL16, is highly conserved, with current knowledge of the enzyme coming from investigation of the protein in mammals. METTL16 (Brown et al., 2016) has a very strict requirement for target methylation because it methylates an adenosine within a nonamer consensus motif (UAC**m**^**6**^**A**GAGAA) only when it is present in a structured RNA context (Doxtader et al., 2018; Mendel et al., 2018; Pendleton et al., 2017). S-adenosylmethionine (SAM) synthetase *MAT2A* mRNA and the spliceosomal U6 small nuclear RNA (snRNA) are the two known targets of mammalian METTL16 (Pendleton et al., 2017; Warda et al., 2017). SAM synthetase is the enzyme responsible for production of the methyl donor SAM, which is required for methylation reactions in the cell. In the case of human *MAT2A* mRNA, there are six methylation sites in the 3′ UTR, each with the motif occupying the single-stranded region of a stem-loop structure (Pendleton et al., 2017; Shima et al., 2017; Warda et al., 2017). Methylation of these sites has been proposed to recruit the nuclear reader protein YTHDC1, which promotes decay of the *MAT2A* mRNA (Shima et al., 2017). However, the central gene-regulatory role of METTL16 appears to be non-catalytic because it has been shown to bind the stem-loop structure to promote splicing of a frequently retained terminal intron (Pendleton et al., 2017). Efficient splicing is critical to produce the MAT2A enzyme and maintain cellular SAM levels. Mammalian METTL16 has a highly conserved N-terminal RNA methyltransferase domain and a C-terminal region that is present only in vertebrates. Importantly, this non-catalytic C-terminal vertebrate-conserved region (VCR) of METTL16 is critical for splicing regulation of the human SAM synthetase *MAT2A* mRNA (Pendleton et al., 2017). Supporting such a non-catalytic splicing stimulation role, loss of mouse *Mettl16* leads to reduced levels of mature *Mat2a* mRNA, causing pre-implantation embryonic lethality (Mendel et al., 2018). This raises the question of the relevance of METTL16’s catalytic activity, which is conserved from bacteria to humans. By investigating the invertebrate and vertebrate orthologs of the enzyme, our study identifies 3ʹ splice-site m^6^A methylation as a conserved mechanism to regulate splicing.

## Results

### The m^6^A transcriptome of *C. elegans*

**Figure S1:**
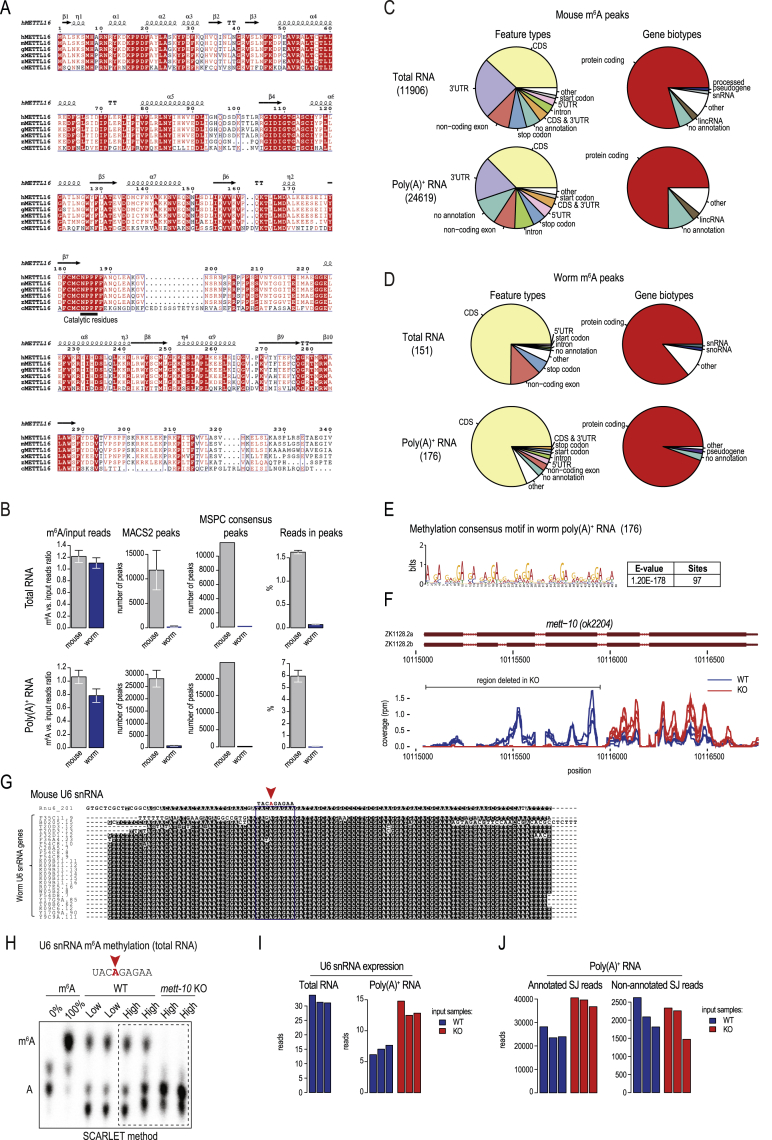
Distribution of m^6^A in the worm transcriptome, related to Figure 1 (A) Protein sequence alignment of the methyltransferase domain of METTL16. h, *Homo sapiens* (NP_076991.3); m, *Mus musculus* (NP_080473.1); g, *Gallus gallus* (NP_001026773.1); x, *Xenopus laevis* (NP_001085334.1); z, *Danio rerio* (NP_001003611.1); c, *Caenorhabditis elegans* (NP_499247.2). Secondary structure features from the human METTL16 core methyltransferase domain (PDB: 6GT5) are indicated: α helices, β strands and η-3_10_ helix. (B) Equimolar amounts of total or poly(A)+ RNA from the adult mouse testes and adult worms (*C. elegans*) were pre-mixed together before performing m^6^A-IP-seq. This allowed us to compare the m^6^A distribution between the species. The worm and the mouse RNAs reveal a similar amount of m^6^A-enriched sequences but only very low number of worm reads pile up as m^6^A peaks. Mean values ± s.d. are plotted (n = 3). (C) Analysis of mouse m^6^A peaks (peak counts are indicated within brackets). (D) Analysis of worm m^6^A peaks (peak counts are indicated within brackets). (E) A consensus sequence identified in the small number (176) of m^6^A peaks identified in worm poly(A)+ RNA. Its significance is not known. (F) RNA-seq analysis of wild-type (WT) and *mett-10* (*ok2204*) knockout (KO) mutant worms showing loss of RNA coverage from the 5′ end of the *mett-10* gene in the KO, consistent with the genomic deletion in the mutant. Biological replicas (n = 3) are plotted separately. (G) Multiple worm U6 snRNA transcripts were identified based on sequence homology to mouse *Rnu6*. The METTL16/METT-10 methylation consensus sequence and position of m^6^A (red arrowhead) are indicated. (H) Detection of m^6^A methylation in U6 snRNA from total RNA using the SCARLET method (STAR Methods). The method allows interrogation of site-specific methylation status (red arrowhead indicates the nucleotide position we examined). The thin-layer-chromatography (TLC) assay used in the protocol is shown. The total RNA is from wild-type (WT) or *mett-10* KO worms, grown on nutrient-high or nutrient-low plates. m^6^A, refers to synthetic RNA oligos without (0%) or with (100%) m^6^A (Table S3), used here as positive controls for the experiment (see STAR Methods). A part (dotted box) of this image is reproduced as Figure 1J. (I) The loss of U6 snRNA methylation in the *mett-10* KO results in slight increase of cellular U6 snRNA levels. Three input replicas are plotted separately for each tested genotype. (J) The loss of U6 snRNA methylation in the *mett-10* KO does not result in overall change in counts of reads covering splice junctions, therefore has no drastic effect on general splicing. Three input replicas are plotted separately for each genotype.

**Figure 1:**
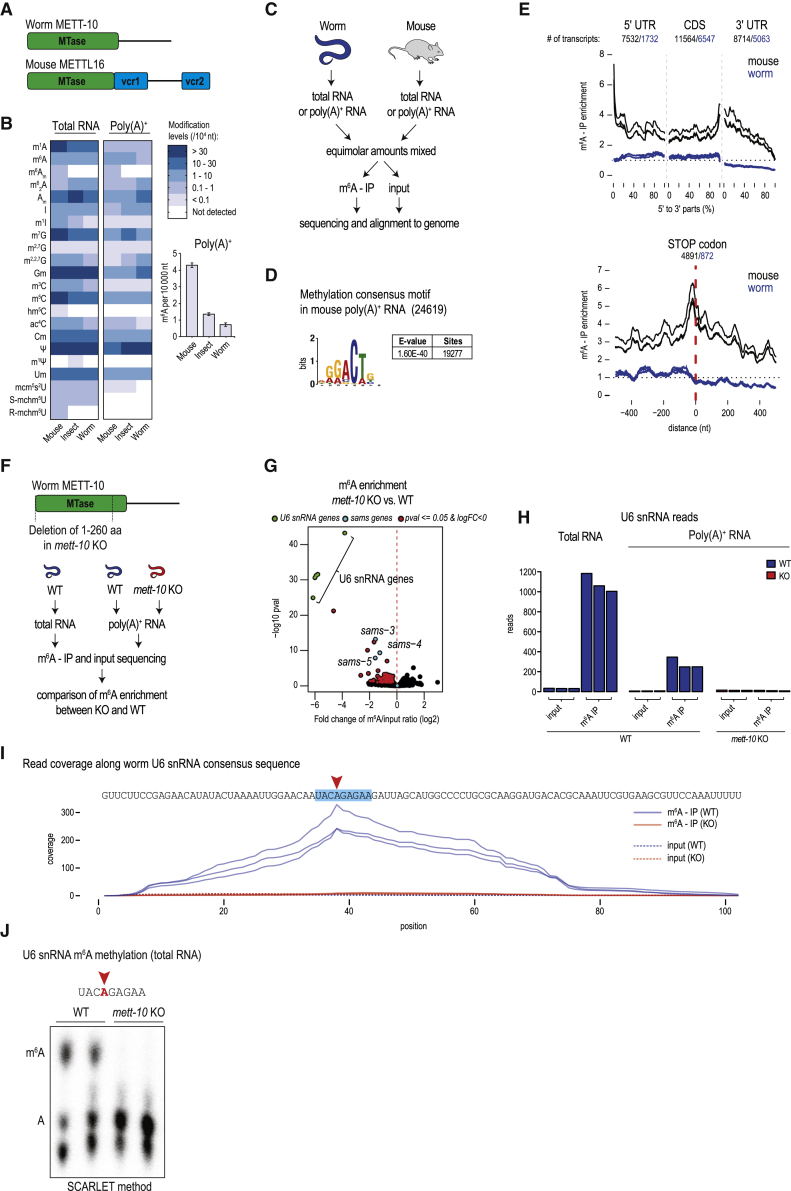
Worm METT-10 is an m^6^A writer for U6 snRNA and SAM synthetase mRNA (A) Domain organization of the m^6^A writers: mammalian METTL16 and *Caenorhabditis elegans* METT-10. MTase, methyltransferase domain; VCR, vertebrate-conserved region. See also Figure S1A. (B) Quantification of RNA modifications in total and poly(A)^+^ RNA from mouse (*Mus musculus*), insect (silkworm, *Bombyx mori*), and worm (*C. elegans*) using liquid chromatography-tandem mass spectrometry (LC-MS/MS). The barplot shows the level of m^6^A in poly(A)^+^ RNA. (C) Scheme for mapping m^6^A sites catalyzed by worm METT-10 with m^6^A-IP-seq. Mouse testes RNA is used as an internal control. See also Figure S1B. (D) The METTL3/METTL14 methylation consensus motif (RRACH) is found on the majority of the mouse m^6^A peaks (total number of peaks in brackets). (E) Meta-analysis of the distribution of m^6^A reads over mouse and worm transcripts. (F) Scheme for identification of m^6^A targets of *C. elegans* METT-10 by m^6^A-IP-seq. See also Figure S1F. (G) Based on decreased m^6^A enrichment in *mett-10* KO worms compared with the control wild type (WT), we identified the indicated transcripts to be targets of METT-10. See also Figure S1G. (H) Worm U6 snRNA is enriched in m^6^A-IP with total and poly(A)^+^ RNA, and this enrichment is lost in the *mett-10* KO . The normalized counts (reads per million [rpm]) are plotted separately for biological replicates (n = 3). (I) Coverage of m^6^A-enriched reads along the worm U6 snRNA sequence identifies the adenosine (red arrowhead), which is part of the conserved UACm^6^AGAGAA motif, that is methylated. Methylation is lost in *mett-10* KO worms. The normalized coverages (rpm) from three biological replicates are plotted separately. (J) Detection of U6 snRNA m^6^A (red arrowhead) in total RNA from WT control or *mett-10* KO worms (in biological duplicates). The thin-layer chromatography (TLC) analysis used in the SCARLET method (STAR Methods) is shown. See also Figure S1H.

To study the conserved role of the catalytic activity of METTL16, we chose the nematode *Caenorhabditis elegans* (hereafter referred to as worm). The worm ortholog METT-10 (Dorsett et al., 2009) contains the highly conserved RNA methyltransferase domain (Figure S1A) but lacks the VCRs found in mammalian METTL16 (Figure 1A). We began the study by detecting various ribose and base modifications in total and poly(A)^+^ RNAs from adult worms (Figure 1B; STAR Methods). RNA from adult mouse testes and an insect cell line (*Bombyx mori* BmN4 cells) were used for comparison. The m^6^A modification is detected in poly(A)^+^ RNA from all three biological sources, including *C. elegans* (Figure 1B), which is important for this study.

To identify worm transcripts that carry the m^6^A methylation, we carried out m^6^A-IP-seq (Ke et al., 2015) with a mixture of poly(A)^+^ RNAs from adult *C. elegans* and mouse testicular RNA (Figure 1C; Table S1; STAR Methods). The mouse RNA serves as an internal technical control because m^6^A sites are already mapped in this system (Wojtas et al., 2017). Compared with over 20,000 mouse peaks, we identified only 176 m^6^A peaks in the worm poly(A)^+^ transcriptome (Figures S1B–S1D), which likely reflects the absence of the METTL3/METTL14 writer complex in worms (Sendinc et al., 2020; van Delft et al., 2017). Indeed, a motif analysis of the mouse peaks reveals the presence of the expected RRACH context (R = A and G; H = A, C, and U) used by the dominant mammalian METTL3/METTL14 writer (Figure 1D; Dominissini et al., 2012; Ke et al., 2015; Meyer et al., 2012), and this is absent in worms. Meta-analysis of m^6^A-IP reads mapping to all mouse transcripts produces the typical profile, characterized by high levels of methylation over the coding sequences with peaks at the 5′ end and over the stop codon (Figure 1E). In contrast, such a pattern of m^6^A distribution is clearly absent over worm sequences (Figure 1E), and a motif search did not recover any particular sequence context for the worm m^6^A-enriched reads (Figure S1E).

### Worm METT-10 is an m^6^A writer for U6 snRNA and SAM synthetase RNA

Having confirmed the presence of m^6^A on worm poly(A)^+^ RNA, we wished to determine the contribution of METT-10 (Dorsett et al., 2009). To search for its methylation targets, we used a comparative analysis of m^6^A-IP-seq datasets to identify poly(A)^+^ transcripts that show reduced m^6^A methylation (m^6^A-IP reads/input reads) in the *mett-10* knockout (KO) mutant (Figure 1F and S1F; Table S2). Of these, the top 20 encode the U6 snRNA sequences (Figures 1G and S1G). U6 snRNA is a non-polyadenylated transcript, so its presence in the poly(A)^+^ dataset is likely due to remnants left after poly(A)^+^ enrichment from total RNA. Consistent with this, a separate m^6^A-IP-seq experiment conducted with total RNA samples shows a higher enrichment of the U6 snRNA reads (Figure 1H). Human U6 snRNA is methylated within a nonamer motif (UAC**m**^**6**^**A**GAGAA) by human METTL16 (Pendleton et al., 2017; Warda et al., 2017), and mapping of m^6^A-IP reads shows that the worm U6 snRNA is also methylated within an identical site (Figures 1I and S1G). Importantly, this m^6^A signal is lost in the *mett-10* KO (Figures 1H and 1I). As an independent validation, we used the SCARLET method, which allows examination of the methylation status in a nucleotide-specific manner (Liu et al., 2013). Analysis of total RNA from adult worms confirms methylation of this specific adenosine within the methylation consensus motif, and this is completely lost in the *mett-10* KO (Figures 1J and S1H). Loss of methylation in the *mett-10* KO has a slightly positive influence on the overall U6 snRNA levels (Figures S1I and S1J).

Other transcripts that display a significant drop in m^6^A levels in the *mett-10* KO are *sams-3*, *sams-4*, and *sams-5* (Figure 1G). These duplicated genes encode the SAM synthetase, the enzyme responsible for production of the methyl donor SAM, which is required for methylation reactions in the cell. We identify worm METT-10 as an m^6^A RNA methyltransferase, and, like its mammalian ortholog METTL16, it has U6 snRNA and SAM synthetase RNA as conserved targets.

### 3ʹ splice site m^6^A methylation of SAM synthetase pre-mRNA inhibits its splicing

**Figure 2:**
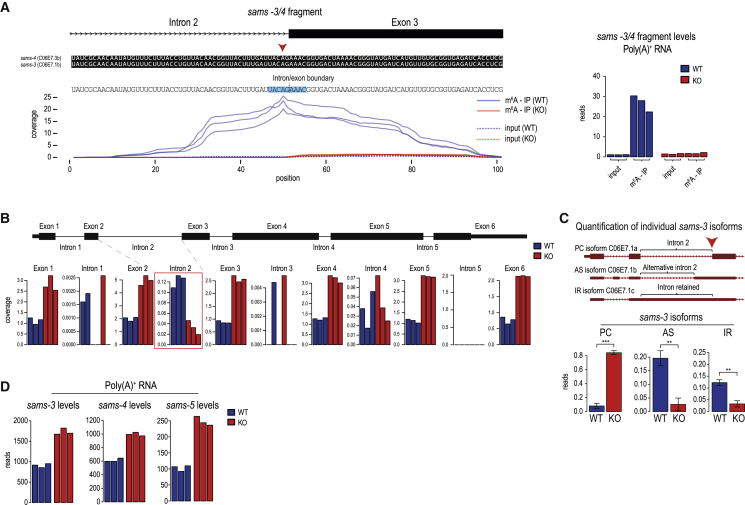
A 3′ splice site m^6^A inhibits splicing of SAM synthetase pre-mRNA (A) Mapping of m^6^A reads identifies the 3′ splice site adenosine (red arrowhead) of intron 2 in the *sams-3/4* pre-mRNA as being methylated, and this methylation is lost in *mett-10* KO worms. The METT-10 methylation consensus motif is highlighted. The normalized coverages (rpm) from three biological replicates are plotted separately. See also Figures S2A and S2B. The barplot shows quantification (rpm) of the reads mapping to the *sams3/4* genomic window . (B) Normalized read coverage (rpm) along the *sams-3* genomic locus shows uniformly increased exonic coverage and lower intron 2 coverage in the *mett-10* KO, suggesting more efficient splicing. Three biological replicates are plotted separately. (C) Three *sams-3* isoforms that differ in utilization of the methylated 3′ splice site are annotated in ENSEMBL. Quantification of the different *sams-3* splice isoforms (rpm; STAR Methods) in WT and *mett-10* KO worms shows an increase in the mature, fully spliced PC isoform in the KO. PC, protein-coding; AS, alternative splice; IR, intron-retained. Mean values ± SD are plotted (n = 3). The p values were calculated using t tests. ^∗∗^p ≤ 0.01, ^∗∗∗^p ≤ 0.001. (D) Read counts (DESeq2 normalized) for the three different *sams* genes in the poly(A)^+^ transcriptome from WT and *mett-10* KO worms show an overall increase in the KO. The three biological replicates are plotted separately. See also Figure S2C.

**Figure S2:**
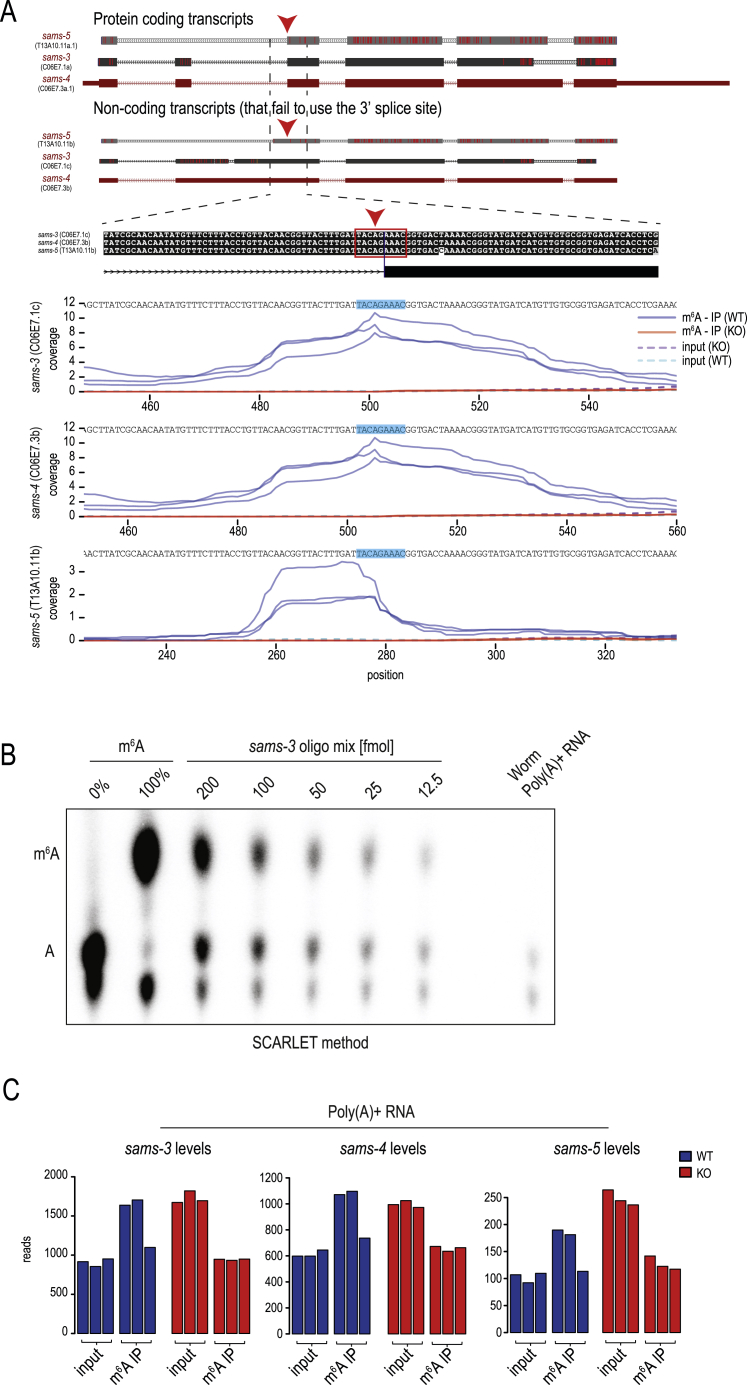
The worm m^6^A writer METT-10 methylates the 3′ ss in an intron of the three *sams* homologs, related to Figure 2 (A) Three highly similar *sams* duplicated genes are present in the *C. elegans* genome. Splicing isoforms that differ in utilization of the methylated 3′ splice site (indicated with red arrowhead) are annotated in ENSEMBL. The cartoon shows the *sams-4* genomic locus and the *sams-3*/*-5* transcripts mapped to the *sams-4* locus using BLAT. All *sams-3/-4/-5* loci encode for the protein-coding isoform which uses the methylated 3′ splice site for splicing, but also non-coding variants where this splice site is not used. Coverage of m^6^A along the intron-exon boundary identifies the methylated adenosine at the 3′ splice site of the three SAM synthetase homologs *sams-3*, *sams-4* and *sams-5*. The m^6^A-IP-seq coverage has insufficient resolution to identify the methylated adenosine (red arrowhead) in *sams-5*. However, methylation is completely absent for all three homologs in the *mett-10* KO. Biological replicas (n = 3) are plotted separately. The METT-10 methylation consensus motif is highlighted. (B) Detection of m^6^A methylation at the 3ʹ splice site in the *sams-3* pre-mRNA using poly(A)+ RNA from adult worms grown on a nutrient-high diet. The SCARLET method was used to specifically probe the 3ʹ splice site adenosine in intron2, but was undetectable. The thin-layer-chromatography (TLC) assay used in the protocol is shown. The same procedure was carried out with a dilution series of control synthetic RNA oligos (an equal mixture of oligos were the target adenosine is either methylated or unmethylated) mimicking the *sams-3* target sequence. Methylation of the oligos can be detected, but efficiency drops with a decrease in the amount of the oligos used. (C) Loss of 3′ splice site methylation results in increased expression of SAM synthetase (*sams*) genes. Compare input reads in WT versus *mett-10* KO from the m^6^A-IP-seq experiment. Biological replicas (n = 3) are plotted separately. The input data from this plot is reproduced in Figure 2D.

Although mammalian METTL16 and worm METT-10 methylate SAM synthetase RNA, mapping of the m^6^A-IP reads reveals very different locations for the modification. There are six methylation sites within the 3′ UTR of mammalian *MAT2A* SAM synthetase mRNA (Pendleton et al., 2017; Warda et al., 2017). In contrast, mapping of reads from three independent m^6^A-IP datasets to the worm genome reveals a single discrete peak over the intron 2/exon 3 junction of the s*ams* pre-mRNAs, and this signal is not detectable in the *mett-10* KO (Figure 2A). This peak is seen when reads were mapped over the duplicated *sams-3*, *sams-4*, and *sams-5* genes (Figure S2A). Because *sams-3* and *sams-4* are identical in sequence at this junction region and, hence, indistinguishable, we refer to these genes together in some of the analyses (Figure 2A). Compared with the methylation motif in worm U6 snRNA and in mammalian targets (UAC**m**^**6**^**A**GAGAA), a variant motif is identified at the *sams* m^6^A peak (UAC**m**^**6**^**A**GAAAC; identical sequences are underlined). Importantly, the methylated adenosine within this motif is at the 3ʹ splice site (AG) of intron 2 (Figures 2A and S2B).

The significance of this finding became clear when we examined sequence databases. In addition to the mature spliced protein-coding (PC isoform) version of the *sams-3/4* transcript, two noncoding versions that fail to use the 3′ splice site within intron 2 are detected (Figures 2C and S2A). One noncoding version is an alternative splice (AS isoform) variant that uses an upstream cryptic 3′ splice site, and the other is an intron-retained version (IR isoform) that retains the complete intron 2. Compared with wild-type (WT) worms, the overall intron 2 read counts are lower in the *mett-10* KO (Figure 2B), indicating its efficient splicing in the absence of 3′ splice site methylation. Consistent with this, quantification of splice junction reads in the RNA sequencing (RNA-seq) datasets shows that this particular 3′ splice site (producing the PC isoform) is used preferentially (∼8-fold higher) in the *mett-10* KO, whereas use of the upstream AS site (producing the AS isoform) and intron 2 retention (IR isoform) is higher in the WT (Figure 2C). This suggests that m^6^A methylation at the 3ʹ splice site prevents its use and, instead, promotes use of an alternative upstream 3ʹ splice site or intron retention. The consequence of this m^6^A-mediated splicing inhibition is a general increase in *sams* mRNA levels in the *mett-10* KO (Figures 2D and S2C). We show that worms use METT-10-mediated 3ʹ splice site m^6^A methylation to inhibit splicing and production of SAM synthetase mRNA.

### An RNA secondary structure is required for m^6^A methylation at the 3ʹ splice site

**Figure 3:**
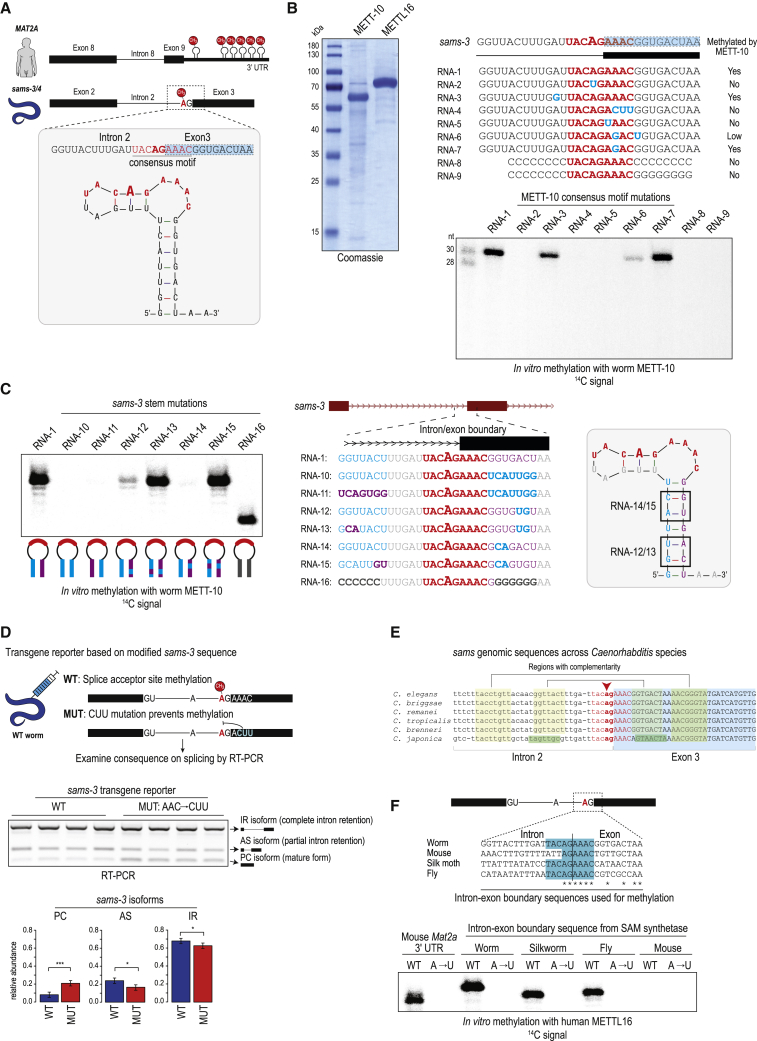
A conserved stem-loop structure containing the 3′ splice site identifies it for methylation by METT-10 (A) Position of m^6^A marks introduced by human METTL16 on the 3ʹ UTR of human *MAT2A* SAM synthetase as well as *C. elegans* METT-10 on the 3ʹ splice site of worm *sams-3* pre-mRNA. A 30-nt RNA fragment (RNA-1; Table S3) spanning the intron 2-exon 3 boundary of the worm *sams-3* gene is predicted to fold into a stem-loop structure, with the METT-10 methylation motif UACm^6^AGAAAC (red) present in the loop region. This is very similar to the substrate requirement of mammalian METTL16. (B) Purification of recombinant worm METT-10 and human METTL16 proteins for *in vitro* methylation assays. Shown are *in vitro* methylation assays with METT-10 and the indicated RNA substrates, based on the *sams-3* intron 2/exon 3 junction sequence, using radioactive ^14^C-SAM as a methyl donor. The UACAGAAAC motif (red) and residues that were mutated (blue) are highlighted. The reaction products were resolved by PAGE and exposed to detect the radioactivity (^14^C) signal. (C) *In vitro* methylation with recombinant METT-10 and the RNA substrates, based on the *sams-3* intron 2/exon 3 junction sequence, carrying mutations in the stem region. (D) Splicing of WT and mutant (MUT) transgene reporter constructs injected into worm gonads. A MUT construct with triple mutations (AAC→CUU) within the methylation consensus motif (in the exon 3 part) increases 3′ splice site use, producing higher amounts of the PC isoform. Barplots depict the mean relative proportion of individual isoforms ± SD (n = 4). The p values were calculated using t tests. ^∗^p ≤ 0.05, ^∗∗∗^p ≤ 0.001. See also Figure S3A for transgene analysis in the *mett-10* KO background. (E) METT-10 consensus motif (red) and regions allowing secondary structure formation (yellow) are conserved in various worm species. Changes (green) in *C. japonica* are compensatory. (F) Sequence alignment of the genomic region at the intron-exon boundary of the SAM synthetase gene from different organisms. The METT-10/METTL16 methylation consensus motif is highlighted (blue). Shown are *in vitro* methylations with ~30-nt RNAs corresponding to the intron-exon boundary sequence, carried out with recombinant human METTL16. The reaction products were resolved by PAGE and exposed to detect the radioactivity (^14^C) signal. See also Figure S3B for the same reactions carried out with worm METT-10.

Methylation by mammalian METTL16 in the *MAT2A* 3′ UTR requires the presence of the methylation consensus motif in the context of a stem-loop structure (Doxtader et al., 2018; Mendel et al., 2018; Pendleton et al., 2017). Similarly, secondary structure prediction shows that a 30-nt RNA fragment of the *sams-3* pre-mRNA that spans the 3′ splice site folds into a stem-loop structure, with the consensus motif (UAC**m**^**6**^**A**GAAAC) occupying part of the loop region (Figure 3A). To confirm that this sequence can be methylated by worm METT-10, we incubated the 30-nt RNA with recombinant full-length worm METT-10 and radioactive ^14^C-SAM as a methyl donor (Figure 3B). The RNA is methylated specifically at the 3′ splice site (AG) because mutation (A→U) of the adenosine abolishes this activity (Figure 3B, compare RNA-1 with RNA-2). Single or triple (CUU) mutations within the consensus motif (Figure 3B, RNA-4 and RNA-5) also abolish *in vitro* methylation activity of METT-10.

The stem region is also critical for methylation because placement of the motif within a single-stranded context (poly-C flanks) kills all activity, and this cannot be rescued by placing the motif alone within an artificial C:G stem (Figure 3B, RNA-8 and RNA-9). Similarly, large-scale mutations that disrupt the stem cannot be rescued (Figure 3C, RNA-10 and RNA-11). Interestingly, 2-nt mutations (RNA-12 and RNA-14) that disrupt pairing within the stem abolish activity, while compensatory mutations (RNA-13 and RNA-15) that restore pairing at these sites can rescue the activity (Figure 3C). Furthermore, a limited 6-bp artificial C:G stem (RNA-16) in the context of the original sequence supports activity (Figure 3C). These results show that the methylation consensus motif and stem-loop formation at the 3′ splice site of the *sams* pre-mRNA are prerequisites for its recognition by METT-10.

### Mutations that abolish 3ʹ splice site methylation alter splicing *in vivo*

**Figure S3:**
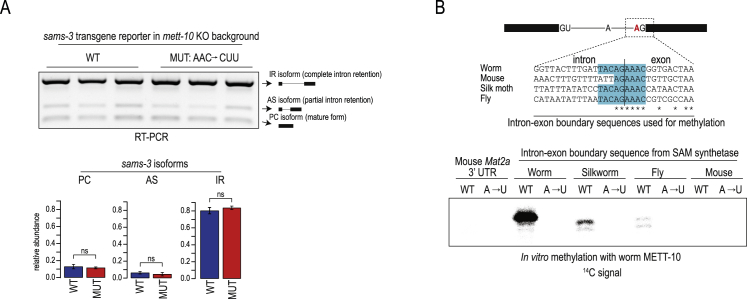
m^6^A methylation of a specific 3′ ss in SAM synthetase pre-mRNA requires a stem-loop structure, related to Figure 3 (A) Wild-type (WT) transgene reporter constructs based on the *sams-3* gene were injected into *mett-10* KO worm gonads and multiple independent progeny lines stably expressing them were derived. Splicing patterns were analyzed by RT-PCR analysis using primers specific to the reporter. A mutated (MUT) construct with triple mutations (AAC →CUU) within the methylation consensus motif (in the exon3) was also tested. Lack of 3′ splice site m^6^A methylation in the KO worms results in similar isoform levels from both WT and MUT constructs. Barplots depict mean relative proportion of individual isoforms ± s.d. (n = 3). See also Figure 3D. PC, protein-coding; AS, alternatively spliced; IR, intron-retained. (B) *In vitro* methylation assay using recombinant worm METT-10 protein and synthetic RNAs. The RNAs correspond to the intron-exon boundary of the SAM synthetase pre-mRNA from the indicated organisms, where the 3′ splice has the METT16/METT-10 methylation consensus motif. Note that the corresponding intron-exon boundary sequence in mouse *Mat2a* pre-mRNA has no consensus motif, unlike the confirmed METTL16 target site in its 3′ UTR. See also Figure 3F for the *in vitro* methylations with human METTL16. It appears that the worm METT-10 is inefficient on targets other than its own *sams* target site, while human METTL16 is active on all targets carrying the methylation consensus motif.

To directly analyze the effect of 3ʹ splice site m^6^A methylation *in vivo*, we prepared a wild-type (WT) transgene splicing reporter construct based on *sams-3* (STAR Methods), where the 3ʹ splice site of intron 2 is methylated by METT-10. We also created a mutant (MUT) version where the methylation consensus motif has the mutations AAC→CUU, which, as we demonstrated, abolish methylation *in vitro* (Figure 3B). These mutations are in exon 3 and do not alter the 3′ splice site. The constructs were injected into the gonads of WT worms, and multiple independent progeny lines showing stable expression of the transgene were established (Figure 3D). Using adult transgenic worms, splicing of these constructs was investigated by reverse-transcriptase polymerase chain reaction (RT-PCR) analysis with transgene-specific primers (Table S3). Each experiment consisted of analysis of three independent progeny lines per construct and was repeated at least three times. We observed three distinct RT-PCR products: the unspliced or IR isoform, the AS isoform, and the correctly spliced mature PC isoform (Figure 3D). The MUT construct, which has mutations preventing m^6^A methylation (Figure 3B), shows increased use of the 3ʹ splice site and efficient splicing *in vivo*, as evidenced by higher PC isoform levels and a decrease in the AS isoform (Figure 3D). This demonstrates the direct role of m^6^A in preventing 3ʹ splice site recognition and inhibition of splicing. Consistent with the requirement of m^6^A methylation, there is no difference in splicing between WT and MUT transgenes when expressed in the *mett-10* KO worms (Figure S3A). We show that it is the presence of an m^6^A at the 3ʹ splice site and not binding of METT-10 per se that regulates splicing and expression of the worm SAM synthetase transcript. This is in stark contrast to the mechanism used by METTL16 to regulate mammalian SAM synthetase pre-mRNA (Pendleton et al., 2017).

Interestingly, *sams* gene sequences surrounding the 3′ splice site from various *Caenorhabditis* species show strong conservation of the capacity to form the stem-loop structure, with the METT-10 methylation motif in the loop region (Figure 3E). Indeed, mutations found in the flanking regions in *Caenorhabditis japonica* are compensatory, allowing continued maintenance of pairing. Moreover, the motif can also be found at *sams* splice sites of other invertebrates, like the fruit fly *Drosophila melanogaster* and the silk moth *Bombyx mori* (Figure 3F), indicating potential evolutionary conservation of this type of splicing regulation among invertebrates.

To functionally validate these insect 3′ splice sites as targets for m^6^A methylation, we carried out *in vitro* methylation assays with a 30-nt RNA spanning the region. We used human METTL16 (Figure 3F) or worm METT-10 (Figure S3B) as enzymes. The insect sequences are methylated specifically at the 3′ splice site (AG) adenosine within the consensus motif because mutation (A→U) of the splice site adenosine abolishes methylation of the RNA (Figure 3F). The homologous junction sequence from mouse *Mat2a* lacks the motif and is not methylated in this experiment, whereas the validated methylation site from the 3′ UTR of mouse *Mat2a* is methylated (Figure 3F). The presence of a conserved methylation motif within a structured RNA at the intron-exon boundary of invertebrate SAM synthetase pre-mRNA transcripts is required for 3′ splice site m^6^A methylation and splicing regulation.

### Methylation of the *sams* 3′ splice site is triggered by a nutrient-rich diet

**Figure 4:**
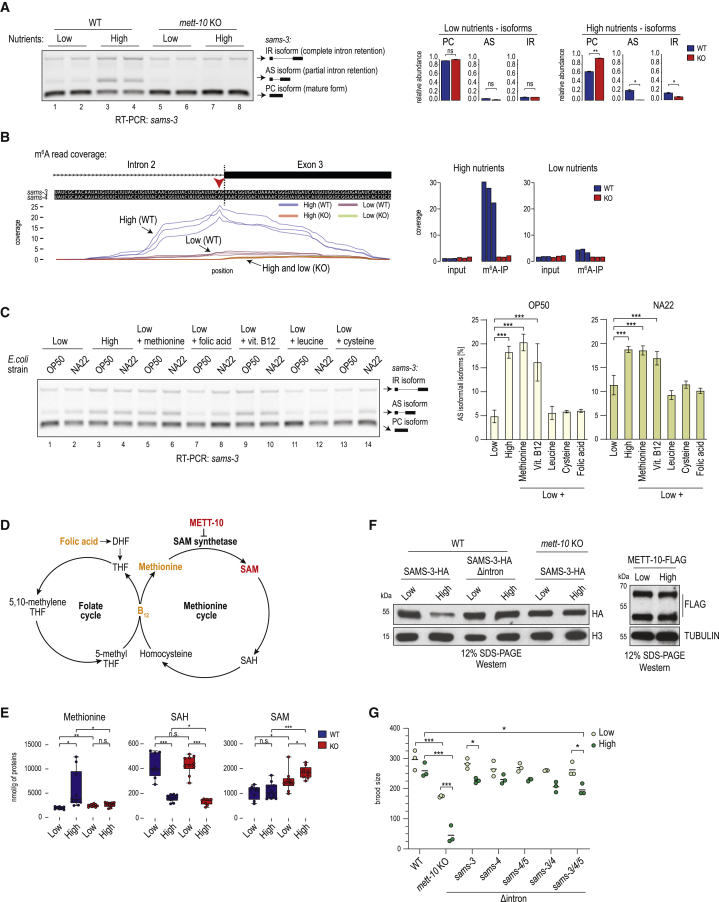
Worms methylate the 3′ splice site of the SAM synthetase transcripts to downregulate their expression in response to a nutrient-high diet (A) WT or *mett-10* KO worms were grown on plates that were high or low in nutrients. Splicing of intron 2 in the *sams-3* gene was monitored by RT-PCR analysis (biological duplicates are shown). Splicing of intron 2 in *sams-3* is different between WT and KO worms only under nutrient-high diet conditions. Barplots depict the mean relative proportion of individual isoforms ± SD (done in biological duplicates). The p values were calculated using t tests. ^∗^p ≤ 0.05, ^∗∗^p ≤ 0.01. See also Figure S4B for RNA-seq data. (B) Mapping of m^6^A-IP-seq reads (n = 3) from WT and *mett-10* KO worms fed on nutrient-high or nutrient-low plates. The m^6^A coverage on the intron 2-exon 3 boundary of the *sams-3* gene is shown. The normalized coverages (rpm) from three biological replicates are plotted separately. See also Figures S4A–S4C. The barplot shows quantification (rpm) of the reads mapping to the *sams-3/4* genomic window shown. The read counts from three biological replicates are plotted separately. (C) A nutrient-high diet inhibits splicing of *sams-3* intron 2 (RT-PCR analysis) in WT worms, as shown by an increased level of the AS isoform. Supplementing a nutrient-low diet with free methionine or vitamin B12 increases splicing inhibition. The barplots (mean ± SD) show quantification of the AS isoform band from three independent biological replicates. The nutrient-low and peptone-rich, nutrient-high media contained OP50 or the NA22 strain of *E. coli*. The p values were obtained by Tukey’s HSD after ANOVA. ^∗^p ≤ 0.05, ^∗∗^p ≤ 0.01, ^∗∗∗^p ≤ 0.001. (D) A simplified scheme showing the methionine and folate cycles. (E) Metabolomics analysis detecting the indicated metabolites. The p values were calculated using t tests and adjusted using Benjamini-Hochberg correction. ^∗^p ≤ 0.05, ^∗∗^p ≤ 0.01, ^∗∗∗^p ≤ 0.001. (F) Western blot analysis of knockin worms expressing SAMS-3-HA or METT-10-FLAG proteins under different diet conditions. One of the worm lines has intron 2 deleted (Δintron) in the *sams-3-HA* gene locus. See also Figures S4D–S4F. (G) Analysis of brood size in worms of the indicated genotypes (Table S5) when grown on nutrient-low or nutrient-high plates. Δintron, deletion of intron having the METT-10 methylated 3ʹ splice site. n = 3 independent experiments, each done in 2–5 technical replicates. The p values were calculated by two-way ANOVA followed by Tukey’s HSD. ^∗^p ≤ 0.05, ^∗∗^p ≤ 0.01, ^∗∗∗^p ≤ 0.001.

**Figure S4:**
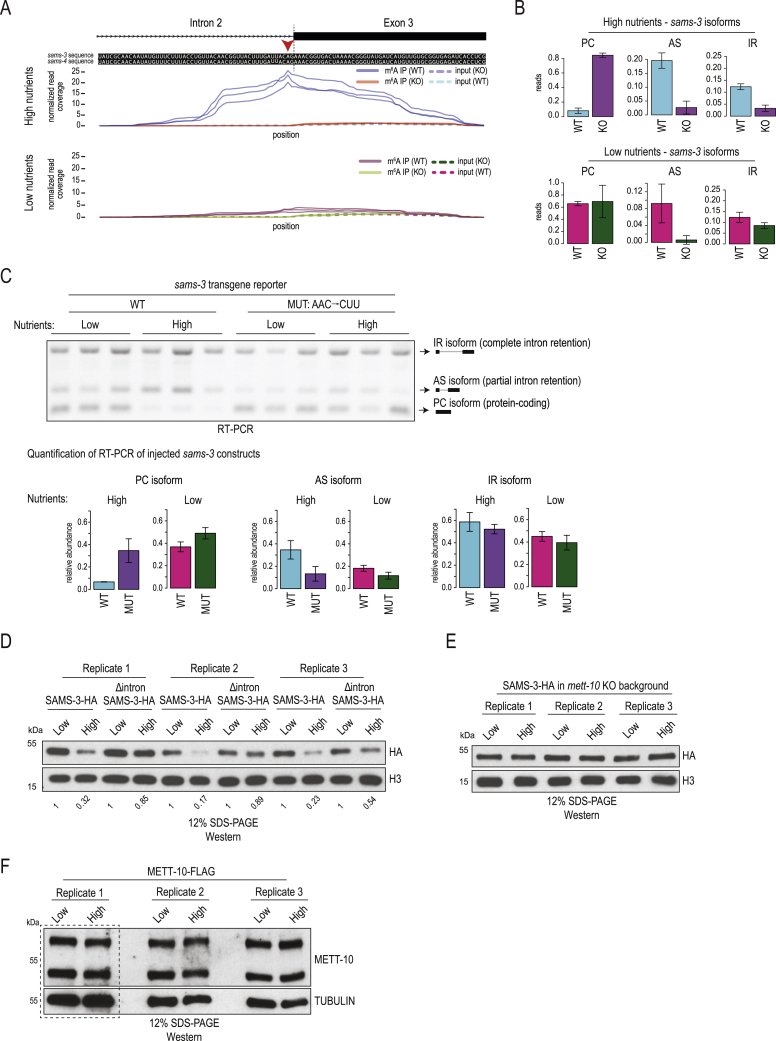
Diet-dependent change in m^6^A RNA methylation of the 3′ ss of SAM synthetase pre-mRNA in *C. elegans* regulates SAMS protein levels, related to Figure 4 (A) The m^6^A-IP-seq read coverages over the identical *sams-3* and *sams-4* intron 2/exon 3 boundary are shown. It shows a difference in methylation between the wild-type (WT) worms grown on nutrient-high and nutrient-low diets. Only the WT worms grown on nutrient-high plates show strong methylation of the 3′ splice site. When WT worms are grown on nutrient-low plates, the methylation is strikingly reduced. In *mett-10* KO the methylation is absent. (B) Quantification of the RNA-seq reads mapping to the various *sams-3* splice isoforms. PC, protein-coding isoform produced by correct use of the 3′ splice site in intron2; AS, alternatively spliced isoform due to use of an upstream 3′ cryptic splice site in intron2; IR, intron-retained isoform due to failure to use the 3′ splice site leading to intron2 being retained. All counts were normalized to library sizes (reads per million, rpm). Mean values are plotted ± s.d. (n = 3). Since there is almost no 3′ splice site methylation in WT worms grown on the nutrient-low plates, the removal of *mett-10* therefore has little effect on *sams-3* splicing (PC isoform). Consequently, both WT and *mett-10* KO worms (under nutrient-low conditions) use the site for splicing and produce predominantly the correctly spliced protein-coding (PC) version of *sams-3*, at levels comparable to KO worms grown on nutrient-high plates. (C) Transgene reporter constructs based on worm *sams-3* sequence were injected into wild-type worms to establish transgenic lines with stable expression. The constructs used had the wild-type (WT) *sams-3* sequence or had mutations (MUT: AAC→CUU) in the methylation consensus motif (on the part that sits on exon 3). This mutation is shown to abolish 3′ splice site m^6^A methylation by recombinant worm METT-10 *in vitro* (Figure 3B). Three independent transgenic isolates expressing the constructs were used in the experiment. The worms were grown on either nutrient-high or nutrient-low plates. Splicing patterns were analyzed by RT-PCR with transgene-specific primers, and quantifications are shown below where mean relative proportions of individual isoforms are plotted ± s.d. A representative ethidium bromide-stained agarose gel showing the resolved cDNA products is shown. On nutrient-high plates, levels of the protein-coding (PC) isoform from WT construct is lower than that seen from the MUT construct, presumably due to 3′ splice site m^6^A methylation in the former. The levels of the PC isoform from both constructs are similar in the nutrient-low plates, presumably, as the former is not methylated under these conditions. (D) Western analysis of SAMS-3-HA expressed from knock-in worm lines with or without intron2 in the *sams-3-HA* genomic locus. The worms were grown in nutrient-high or nutrient-low plates (Table S4). Three biological replicates are shown. Quantified HA signal normalized to that from endogenous histone H3 levels is shown below, with the value in nutrient-low diet being set to 1. Levels of SAMS-3-HA is reduced in nutrient-high diet condition, and this reduction is attenuated in the absence of intron2 in the *sams-3-HA* locus. The lysate from replicate #2 was re-run in the gel shown in Figure 4F. (E) Western analysis of SAMS-3-HA expressed from knock-in worm lines, in the *mett-10* background. Worms were grown on a nutrient-high or nutrient-low diet. Three biological replicates are shown. The lysate from replicate #2 was re-run in the gel shown in Figure 4F. (F) Western analysis of METT-10-FLAG expressed from knock-in worms grown in nutrient-high and nutrient-low plates. Three biological replicates are shown. Part of this image (replicate #1, dotted box) was reproduced in Figure 4F.

*C. elegans* is a bacterium-eating soil nematode that proliferates on rotting vegetal substrates (Félix and Duveau, 2012; Shtonda and Avery, 2006), but it is maintained in the laboratory on food that consists of different strains (OP50 or NA22) of *Escherichia coli*. For all experiments described above, where we noted 3ʹ splice site methylation-mediated splicing inhibition, the worms were grown on nutrient-high agar plates (peptone-rich medium + NA22 strain; Table S4). Changing the diet to nutrient-low agar plates (peptone-poor medium + OP50 strain; Table S4) led to the surprising loss of this splicing regulation and a similar isoform expression pattern among WT and *mett-10* KO worms (Figure 4A). RT-PCR analysis shows that intron 2 of the *sams-3* transcript is spliced efficiently in WT worms grown on nutrient-low agar plates, as evidenced by reduced levels of the AS isoform (Figure 4A, lanes 1 and 2 versus lanes 3 and 4). In fact, the splicing pattern in WT worms grown on nutrient-low plates very much resembled the pattern seen in the *mett-10* KO (Figure 4A), as if m^6^A methylation on the 3′ splice site was absent in WT worms. This diet-dependent change in splicing pattern of endogenous *sams-3* was confirmed by RNA-seq analysis (Figure S4B) and also validated with our transgene reporter constructs based on *sams-3* (Figure S4C).

To directly establish that splice site m^6^A methylation responds to a change in diet, we carried out m^6^A-IP-seq with poly(A)^+^ RNA from WT and *mett-10* KO worms grown on the two different diets. Strikingly, WT worms grown on nutrient-high plates display strong m^6^A methylation of the 3′ splice site within intron 2 of the *sams-3* pre-mRNA, whereas this is reduced dramatically when WT worms are grown on nutrient-low plates (Figures 4B and S4A). The *mett-10* KO lacked this methylation under all conditions (Figure 4B), and, consequently, the splicing patterns were not altered when worms were grown on the different media (Figures 4A and S4B). This allows us to conclude that 3′ splice site m^6^A methylation takes place in response to a nutrient-high diet to inhibit proper splicing and expression of SAM synthetase pre-mRNA.

### m^6^A-mediated inhibition of splicing represents negative feedback regulation of SAM levels

Because RNA methylation depends on SAM as a methyl donor, we examined whether the pathway serves to regulate cellular SAM levels by feedback inhibition. To investigate this further, we asked which constituents in the diet are responsible for triggering splice site methylation. Keeping the bacterial strain constant (NA22 or OP50), we prepared plates with nutrient-low medium or peptone-rich nutrient-high medium (Figure 4C). Worms were grown on such plates, and RT-PCR analysis was conducted to examine splicing of intron 2 in the endogenous *sams-3* pre-mRNA transcript. Irrespective of the bacterial strain used, the nutrient-high medium is responsible for strong splicing inhibition, as determined by quantification of the AS isoform (Figure 4C). Nevertheless, the level of splicing inhibition in nutrient-low plates is slightly higher when the NA22 bacterial strain is used, but the major driving factor was still the peptone-rich nutrient-high medium (Figure 4C).

Production of SAM requires enzymatic activities represented in the inter-linked methionine and folate cycles (Figure 4D). Briefly, SAM is produced from ATP and methionine by the SAM synthetase (*sams* in worms) within the methionine cycle, whereas the downstream by-product homocysteine is regenerated to methionine via methionine synthase, which requires folate (5-methyl tetrahydrofolate) and the co-factor vitamin B12. Importantly, the key metabolites, like the essential amino acid methionine, folic acid, and vitamin B12, are all acquired through the diet. Consistent with this, supplementing the nutrient-low medium with additional free methionine or vitamin B12, which directly enhances SAM production via the methionine cycle, triggered splicing inhibition of *sams-3* (as indicated by AS-isoform levels) similar to that seen with the nutrient-high medium (Figure 4C). However, supplementation with amino acids not involved in the methionine cycle (leucine and cysteine) or folic acid, which feeds into the folate cycle, did not lead to splicing inhibition. These results support a model of regulation by feedback inhibition, where constituents in the diet that directly increase cellular SAM levels via the methionine cycle trigger 3′ splice site m^6^A methylation and splicing inhibition/alternative splicing of SAM synthetase pre-mRNA. This ensures optimal cellular SAM levels. Interestingly, it is known that a diet of the OP50 *E. coli* strain causes vitamin B12 deficiency in worms (Revtovich et al., 2019), probably explaining the reduced *sams-3* splicing inhibition compared with the NA22 strain (Figure 4C), and it also explains the reduced recycling of the by-product S-adenosylhomocysteine (SAH) via the methionine cycle under the nutrient-low diet (with the OP50 strain) condition (Figure 4E).

Validating the above model, metabolomics analyses (Figure 4E) show that, although WT worms are able to control SAM levels, the *mett-10* KO fails to do so. When grown on a nutrient-high diet that supplies an abundance of methionine, WT worms are able to maintain similar levels of SAM as under nutrient-low diet conditions (Figure 4E). Loss of *mett-10* upsets this homeostasis, resulting in elevated SAM concentrations under both diet conditions, with the levels being higher under the nutrient-high condition (Figure 4E). Thus, conditions that favor increased SAM production (such as nutrient-high diet) trigger m^6^A methylation of the splice site in intron 2 of the *sams-3/4* pre-mRNA to inhibit production of the PC isoform version of SAM synthetase mRNA, regulating SAM biosynthesis. To directly verify protein levels of the enzyme during this regulation, we created a worm strain with SAMS-3 hemagglutinin (HA)-tagged at the endogenous locus and then derived a strain with intron 2 removed from the gene (STAR Methods; Table S5). Consistent with the RNA analyses, we observe a reduction in SAMS-3-HA protein levels under the nutrient-high diet condition (Figures 4F and S4D), and this depends on METT-10 and the presence of intron 2 in the *sams-3-HA* genomic locus (Figures 4F, S4D, and S4E). The level of the RNA methyltransferase (METT-10-FLAG; STAR Methods) does not change under the two dietary conditions (Figures 4F and S4F). Thus, m^6^A-mediated reduction in protein levels of a key enzyme within the methionine cycle explains how WT worms cope with a diet that fuels this biosynthetic pathway to ensure SAM homeostasis.

Loss of *mett-10* results in a fertility defect phenotype (Dorsett et al., 2009), and here we examined the effect of diet. Compared with WT control animals, the *mett-10* KO has a reduced brood size with a nutrient-low diet, but this becomes worse with a nutrient-high diet, with very few progenies (Figure 4G). Interestingly, a triple-mutant worm strain (Table S5) lacking intron 2 in the three *sams* genes (*sams-3*^*Δintron2*^, *sams-4*^*Δintron2*^, and *sams-5*^*Δintron2*^) also shows a small but significant reduction in brood size (Figure 4G). This shows that the ability to tune down SAM levels in response to a rich diet, using the m^6^A-mediated splicing inhibition pathway we describe here, contributes to ensuring normal fertility in worms. Finally, the difference in the severity of the phenotypes of the *mett-10* KO and the triple mutant points to the existence of additional METT-10 targets that are required for fertility.

### 3ʹ splice site m^6^A methylation inhibits splicing in mammalian cells

**Figure 5:**
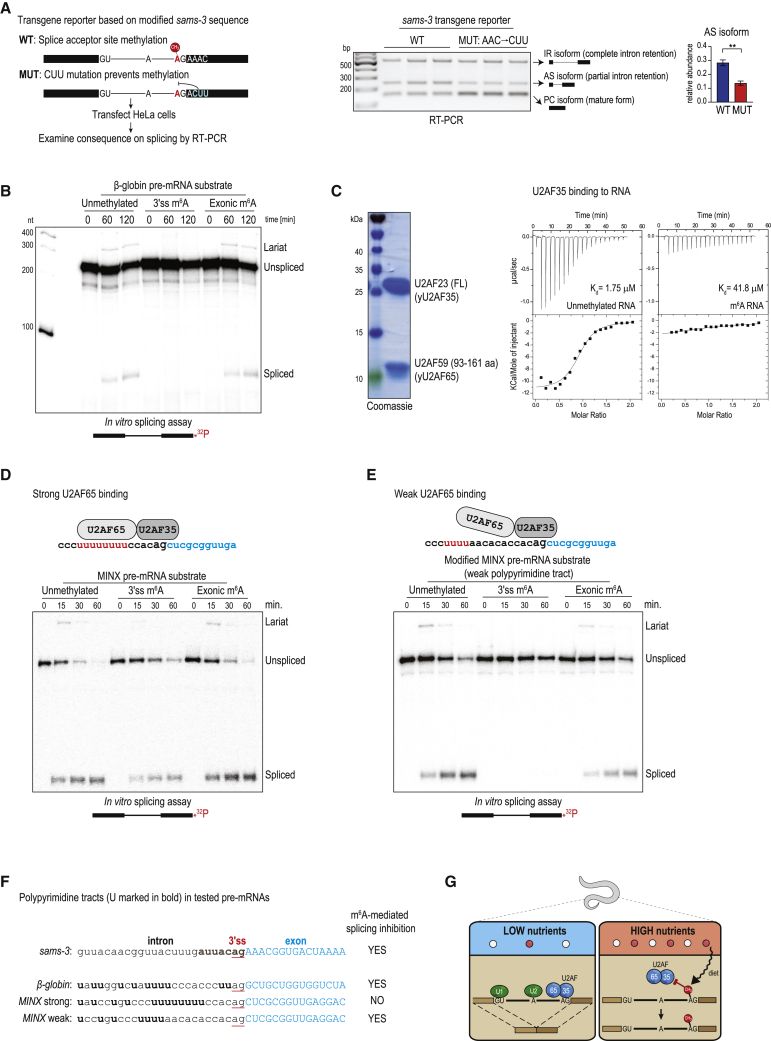
A 3′ splice site m^6^A inhibits splicing in human cells and blocks its recognition by U2AF35 (A) Worm transgene reporter constructs based on *sams-3* were transfected into human HeLa cells, and splicing patterns were analyzed by RT-PCR. A MUT construct with triple mutations (AAC→CUU) within the methylation consensus motif (in the exon 3 part) increases 3′ splice site use, producing lower amounts of the AS isoform. The barplot depicts the mean relative proportion of the AS isoform to the sum of all isoforms ± SD (n = 3). The p value was calculated using a t test. ^∗∗^p ≤ 0.01. See also Figure S5B for all replicates. (B) *In vitro* splicing assay with HeLa S3 nuclear extracts. The human β-globin pre-mRNA substrate is spliced correctly, whereas the same substrate with an m^6^A methylated 3′ splice site (ss) remains unspliced. The presence of the methyl mark on the exonic part does not inhibit splicing. A band corresponding to the lariat intermediate is visible in lanes where the substrate is spliced correctly. Substrates were incubated for different durations (time in minutes) with the extracts. See also Figure S5C. (C) ITC experiments reveal that the full-length (FL) yeast U2AF35 (stabilized with a fragment of yeast U2AF65; STAR Methods) strongly binds an unmethylated RNA substrate mimicking the 3′ ss (AG), whereas the presence of an m^6^A mark decreases affinity. The quality of the recombinant protein used is shown. See also Figure S6. (D) Splicing assays with the MINX pre-mRNA substrate. 3′ ss m^6^A does not inhibit splicing of this substrate, which has a strong polypyrimidine tract. (E) Mutations that weaken the polypyrimidine tract in MINX pre-mRNA make it sensitive to inhibition by 3′ ss m^6^A. The presence of the methyl mark on the exonic part does not inhibit splicing. (F) Sequence of the 3ʹ end of the intron in the splicing substrates, showing the polypyrimidine tract (bold) and the 3ʹ ss. A similar region from worm *sams-3* pre-mRNA is also shown, with the consensus ss motif shown (bold). (G) Model showing how 3′ ss m^6^A methylation under nutrient-high conditions prevents binding of U2AF35, leading to inhibition of splicing of *sams* pre-mRNA in worms.

**Figure S5:**
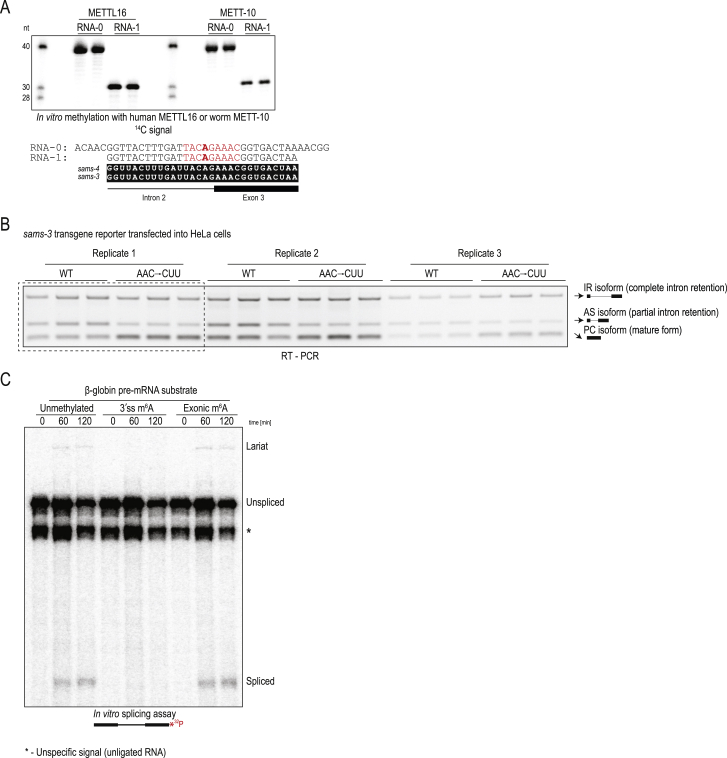
The 3′ ss m^6^A methylation-mediated splicing inhibition is conserved in human cells, related to Figure 5 (A) *In vitro* methylation assay using recombinant human METTL16 or worm METT-10 proteins and radioactive ^14^C-SAM as the methyl donor, using RNAs (two different lengths) corresponding to the intron 2-exon 3 boundary of the worm *sams-3* gene. The methylation consensus motif (red) and target adenosine (in bold) are shown. The reaction products were resolved by PAGE, the gel was stained with Methylene Blue to reveal the RNAs (to assure similar levels), and exposed to detect the radioactivity signal (^14^C). The human METTL16 is able to recognize and methylate the worm *sams-3* target site, allowing us to test worm transgene reporter constructs in human HeLa cells. See also Figure 5A, and below. (B) RT-PCR analysis of the transcripts produced from worm *sams-3* transgene construct transfected into HeLa cells. Wild-type (WT) construct with the 3′ splice site which can be methylated by human METTL16 shows different splicing pattern when compared to the construct with mutations (MUT: AAC→CUU) in the methylation consensus motif (on the part that sits on exon 3). Compare ratios of alternatively spliced (AS) and correctly spliced protein-coding (PC) isoforms. Three biological replicates, each with three technical replicates, were used to quantify the individual isoforms and produce the barplot in Figure 5A. Part of this panel (replicate #1, dotted box) is reproduced in Figure 5A. (C) *In vitro* splicing assay shows that an artificially introduced 3′ splice site (3′ ss) m^6^A within the human beta-globin pre-mRNA abolishes its splicing in human HeLa nuclear extracts, with neither the fully spliced product nor the lariat intermediate being detected. Presence of a single exonic m^6^A has no effect on splicing. See also Figure 5B. A major RNA band (indicated with an asterisk) below the unspliced RNA substrate is an irrelevant non-ligated species leftover from production of the splint-ligated RNA substrate (see STAR Methods)

The above experiments show that worm METT-10 regulates splicing of *sams* pre-mRNA through m^6^A methylation of a specific 3ʹ splice site. Because the basic mechanism of splicing is highly conserved from yeast to human (Fica and Nagai, 2017; Galej, 2018; Kastner et al., 2019), we wanted to find out whether the m^6^A-mediated inhibitory pathway can be active in the mammalian system. To investigate this, we transfected the transgene reporter constructs based on worm *sams-3* into human HeLa cell cultures (Figure 5A). We already know that the 3′ splice site within worm *sams-3* RNA can be methylated by human METTL16 (Figures 3F and S5A). Strikingly, RT-PCR analysis of this reporter with the WT sequences revealed a splicing pattern similar to that seen when the same construct was expressed in worms, with 3′ splice site methylation reducing its use and promoting alternative splicing (AS isoform) via use of a cryptic upstream 3ʹ splice site (Figures 5A and S5B). As seen in worms, the transgene reporter with mutations (MUT, AAC→CUU) in the methylation consensus motif allows increased 3′ splice site use, reducing levels of the AS isoform (Figures 5A and S5B). Thus, using this ectopic reporter system, we demonstrate that human METTL16 can catalyze 3′ splice site m^6^A methylation, which leads to splicing modulation in human cells.

Next we wanted to know whether the observed splicing modulation is a direct consequence of the m^6^A mark or whether the stem-loop structure that is required for recruitment of METTL16 plays any role. To demonstrate that the inhibitory effect is directly due to the presence of m^6^A, we artificially introduced an m^6^A at the 3′ splice site (by splint ligation; STAR Methods) of the unrelated human β-globin pre-mRNA and carried out *in vitro* splicing assays (Krainer et al., 1984). To this end, we prepared ^32^P-labeled splicing substrates and incubated them with human HeLa S3 extracts (Figure 5B). Splicing takes place via two transesterification reactions (Fica and Nagai, 2017; Shi, 2017; Will and Lührmann, 2011). In step 1, the free 5′ exon and the intron lariat-3′ exon intermediate are produced. In step 2, exon ligation joins the 5′ exon with the 3′ exon, releasing the branched lariat. Splicing of the unmethylated substrate proceeded normally, as expected (Padgett et al., 1984; Ruskin et al., 1984), with production of the lariat intermediate and the mature spliced product observed (Figures 5B and S5C). However, splicing of the substrate with the m^6^A modification at the 3ʹ splice site was blocked completely because the lariat and the mature product were absent (Figures 5B and S5C). Placing the m^6^A mark in the exonic part of the substrate did not hinder splicing (Figures 5B and S5C), demonstrating the specificity of the 3ʹ splice site inhibitory mechanism. Thus, we conclude that the human splicing machinery is also sensitive to the presence of m^6^A at the 3′ splice site, and this directly inhibits the first step of the splicing reaction.

### m^6^A methylation prevents splice site recognition by the essential splicing factor U2AF35

Recognition by splicing factors of the key *cis* elements within the pre-mRNA is critical for initiation of splicing in metazoans. The 5ʹ splice site is recognized by the U1 snRNP, the branchpoint sequence (BPS) by the mammalian branchpoint binding protein (mBBP)/SF1, and the 3ʹ splice site is bound by the U2 auxiliary factor (U2AF). mBBP/SF1 and U2AF then promote recruitment of the U2 snRNP, which pairs with the branch-site sequence. U2AF is a heterodimer composed of the U2AF35 and U2AF65 subunits (Zamore and Green, 1989). While U2AF65 recognizes the polypyrimidine tract that precedes the AG dinucleotide at the intron-exon junction (Sickmier et al., 2006; Zamore et al., 1992), U2AF35 has been shown to directly contact the 3′ splice site AG dinucleotide (Merendino et al., 1999; Soares et al., 2006; Wu et al., 1999; Zorio and Blumenthal, 1999a; Zuo and Maniatis, 1996). U2AF35 is highly conserved from fission yeast to human and essential for splicing *in vivo* in worms (Zorio and Blumenthal, 1999b) and flies (Rudner et al., 1996).

**Figure S6:**
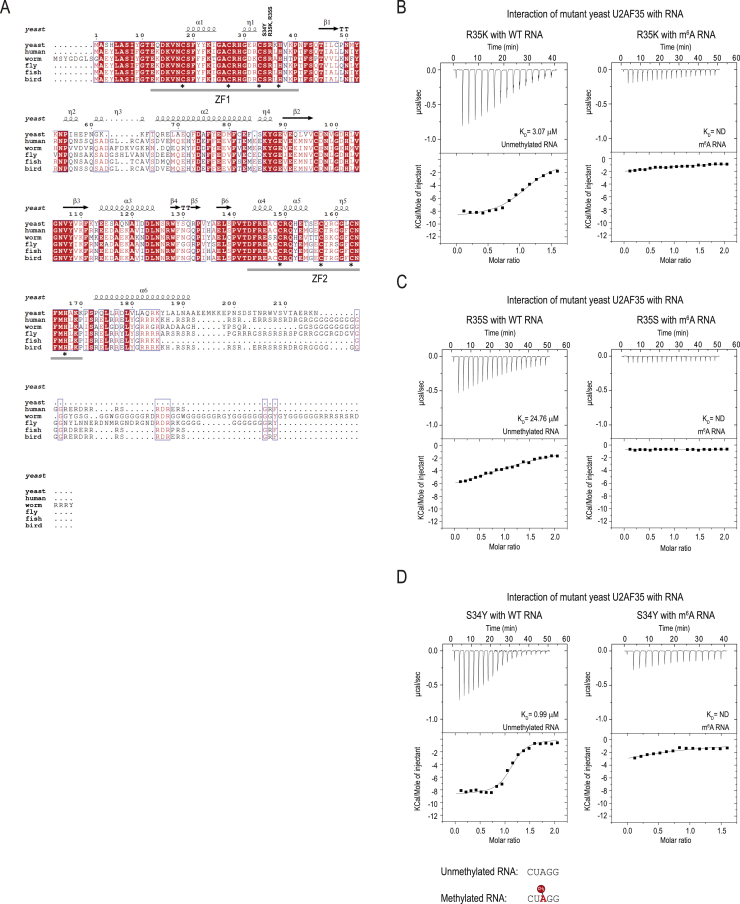
3′ ss m^6^A methylation blocks splicing by hindering its recognition by the U2AF35 splicing factor, Related to Figure 5 (A) Comparison of U2AF35 protein sequence among different species. The protein complex used for ITC experiments consists of the full-length U2AF23 (*S. pombe* U2AF35) and 93-161 aa of U2AF59 (*S. pombe* U2AF65). The Zinc Finger 1 (ZF1) and ZF2 domains in the yeast protein are highly similar to that in other organisms. The secondary structure features of *S. pombe* U2AF23 (PDB: 4YH8) is shown above the alignment: α helices, β strands and η-3_10_ helix. The asterisks at the bottom of the alignment indicate residues coordinating the zinc ion, while the residues we mutated are indicated on the top. (B-D) Isothermal calorimetry (ITC) experiments reveal that the yeast U2AF35 (in complex with a fragment of U2AF65) strongly binds an unmethylated RNA substrate (5′CU**AG**G) mimicking the 3′ splice site AG, while presence of an m^6^A mark decreases the affinity. See Figure 5C. Two mutations of Arginine 35 that is involved in recognition of the splice site adenosine were made. A conservative mutation to a positively charged lysine (R35K) or to a non-conservative mutation to uncharged serine (R35S). The R35K mutation was made to see if the shorter side-chain of lysine could allow recognition of m^6^A. We also made a mutation in the serine 34, which is frequently mutated to phenylalanine or tyrosine in human cancers, so we tested the S34Y mutant. Importantly, the two mutations replacing arginine 35 reduced binding to the unmethylated RNA, all three mutations did not bind to the methylated RNA.

This prompted us to examine whether 3′ splice site methylation can hinder U2AF35 binding. Our attempts to express full-length human or worm U2AF35 alone in a recombinant form were unsuccessful. However, we could stabilize fission yeast (*Schizosaccharomyces pombe*) full-length U2AF35 by expressing it in complex (Yoshida et al., 2015) with a minimal fragment of U2AF65 (the U2AF35-interacting region) lacking the RNA binding domains (Zamore et al., 1992; Figure 5C; STAR Methods). U2AF35 with its two zinc fingers (Figure S6A) is the only component in this complex with the ability to bind RNA, hence, hereafter, this preparation will be referred to as U2AF35. We used a short RNA fragment mimicking the 3ʹ splice site (AG) to test interactions with U2AF35. Isothermal calorimetry (ITC) experiments revealed that, although U2AF35 strongly (K_D_ = 1.75 μM) interacts with the unmethylated RNA, the presence of 3ʹ splice site m^6^A decreases the affinity by an order of magnitude (K_D_ = 41.8 μM) (Figures 5C and S6B–S6D). Thus, the 3ʹ splice site m^6^A inhibits splicing by physically hindering its recognition by the essential splicing factor U2AF35.

### Splice site m^6^A methylation inhibits splicing of AG-dependent introns

Of the different splicing signals within the intron, the polypyrimidine tract is the most variable. Its composition, measured by the number of uridines in the tract (Singh et al., 1995; Zamore et al., 1992), defines the strength of the 3ʹ splice site (Moore, 2000; Reed, 1989). *In vitro* splicing of an intron with a strong polypyrimidine tract (AG-independent introns) requires only U2AF65 (Valcárcel et al., 1996; Zamore et al., 1992), whereas that with a weak polypyrimidine tract (AG-dependent introns) additionally requires U2AF35, which recognizes the AG dinucleotide (Wu et al., 1999). Thus, although the conserved AG dinucleotide at the 3ʹ splice site is only required for the second step of splicing during exon ligation, AG-dependent introns require its recognition by U2AF35 early during spliceosome assembly and for the first step of splicing (Reed, 1989; Wu et al., 1999).

To determine whether splicing inhibition by 3′ splice site m^6^A depends on the type of intron involved, we experimented with the MINX (an adenovirus major late pre-mRNA derivative) splicing substrate (Figures 5D and 5E). Compared with the β-globin pre-mRNA substrate, the MINX substrate has a strong polypyrimidine tract with a run of eight uridines (U_8_), identifying the intron as AG independent (Figure 5F). When incubated with HeLa S3 extracts, the unmethylated MINX substrate is spliced, with the lariat intermediate and spliced product visible (Figure 5D). Interestingly, the MINX substrate with an m^6^A-methylated 3′ splice site is also spliced, albeit with slightly lower efficiency (Figure 5D). This is contrary to the observation for the β-globin pre-mRNA substrate, where the 3′ splice site m^6^A completely inhibits splicing (Figure 5B). This suggests that the inhibitory effect of 3′ splice site m^6^A is dependent on the type of intron being regulated. To verify whether this is due to the presence of a strong polypyrimidine tract (U_8_), we introduced mutations to convert the MINX construct into a substrate with only four uridines (U_4_) (Figure 5F). Strikingly, splicing of such a MINX pre-mRNA substrate with a weakened polypyrimidine tract (effectively making it an AG-dependent intron) is abolished completely in the presence of a 3′ splice site m^6^A (Figure 5E). An exonic methylation does not affect splicing of either substrate. This indicates that AG-dependent introns with a weakened polypyrimidine tract are sensitive to a 3′ splice site m^6^A because they require recognition by U2AF35 of the AG dinucleotide for efficient U2AF recruitment.

In this context, it is worth mentioning that introns in *C. elegans* lack the polypyrimidine tract consensus sequence as in other metazoans but instead have a conserved consensus sequence, U_4_CAG (Figure 5F), at the 3′ end (Blumenthal and Steward, 1997). The U_4_C sequence in this consensus sequence is bound by worm U2AF65, but this association is enhanced by simultaneous binding of worm U2AF35 to the AG dinucleotide (Zorio and Blumenthal, 1999a). We show that splicing of AG-dependent introns, which rely on U2AF35 binding to the AG dinucleotide to recruit the U2AF complex, can be regulated by m^6^A methylation of the 3′ splice site (Figure 5G).

### Search for 3′ splice sites potentially regulated by mammalian METTL16

**Figure S7:**
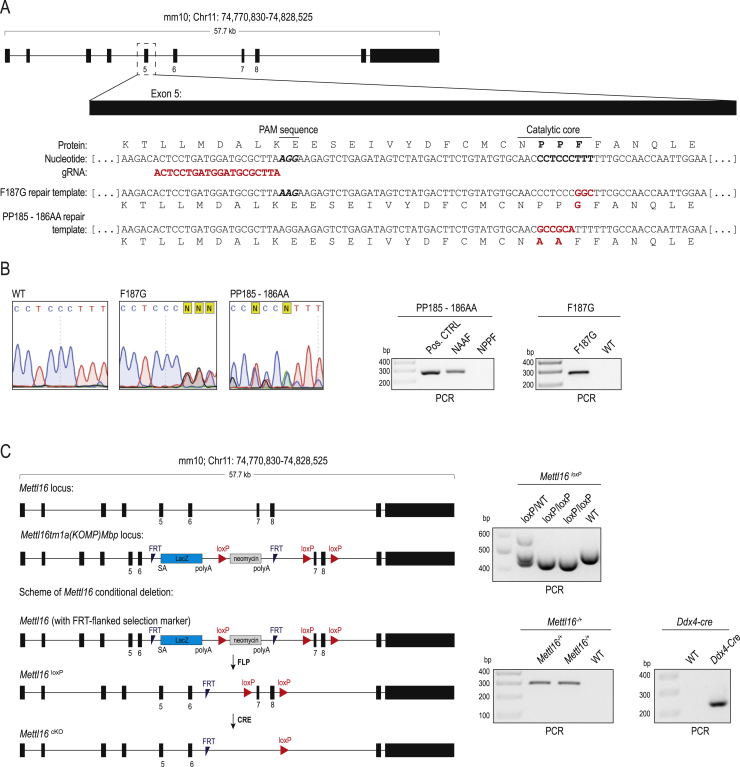
Creation of a mouse KI and cKO mutants for *Mettl16*, related to Figure 6 (A) Strategy for generation of the *Mettl16* knockin (KI) point mutant mice. A part of the genomic sequence of the *Mettl16* exon 5 and the predicted protein sequence encoded are shown. A single guide RNA (gRNA) targeting this region was used to guide Cas9 endonuclease activity and homology-mediated repair to introduce nucleic acid mutations that eventually result in the following amino acid changes: F187G (RNA binding mutant) and PP185-186AA (catalytic dead mutant). Sequence of part of the repair templates bringing the mutations (in red) are shown. (B) Examples of Sanger sequencing of genomic PCR to detect the WT, F187G and PP185-186AA *Mettl16* alleles (from mouse tail DNA). Representative ethidium bromide-stained agarose gels showing resolved PCR products is shown. Primer sequences are provided in Table S3. (C) Strategy for creation of the floxed *Mettl16* allele. The mouse line with floxed *Mettl16* allele and an inserted FRT-flanked selection markers cassette (LacZ and neomycin) was obtained from the KOMP repository at UC, Davis. Animals were crossed to remove the selection markers (STAR Methods). Using further crosses, we then brought together the floxed (*Mettl16*^*loxP*^) allele and the *Mettl16* null allele (*Mettl16 -*). Crosses between *Mettl16*^*loxP/-*^ and *Mettl16*^*loxP/+*^*; vasa-Cre* partners gave us the *Mettl16*^loxP/-^; *vasa-Cre* mice = conditional knockout (cKO) mutant. In the cKO, the gene is deleted in the male and female germline (starting from embryonic day E14.5 in the male germline). Representative ethidium bromide-stained agarose gels showing resolved PCR products detecting the different alleles and Cre driver is shown. Primer sequences are provided in Table S3.

**Figure 6:**
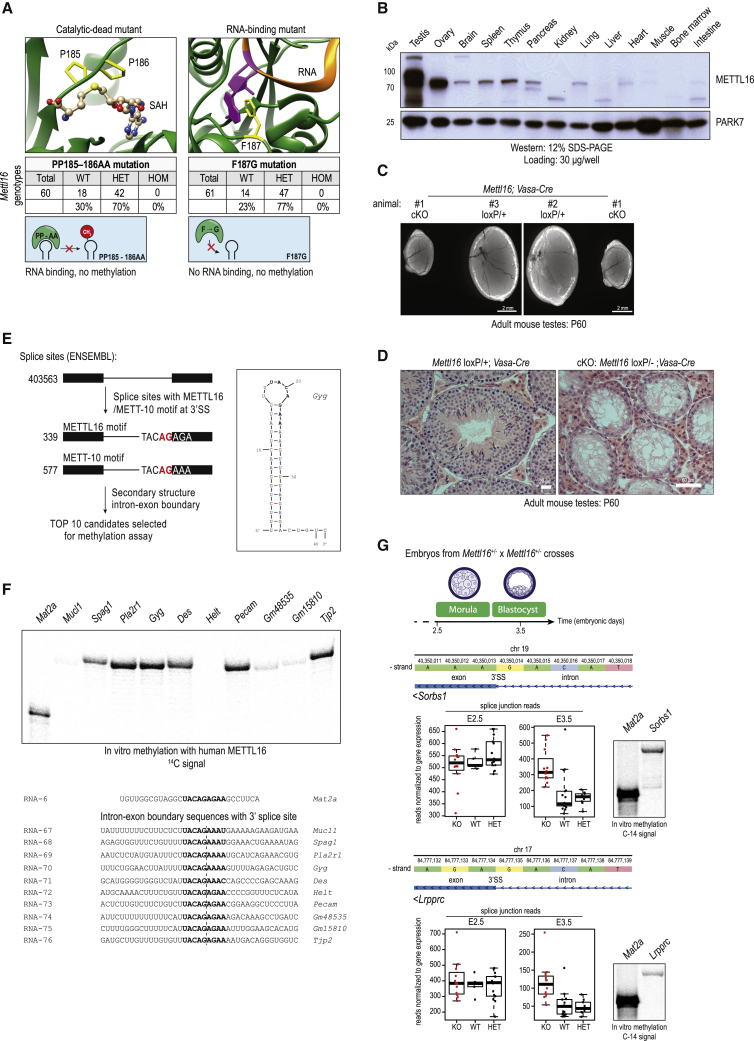
RNA m^6^A methylation activity of mouse METTL16 is essential for development and has the potential to methylate the 3′ ss of target RNAs (A) Analysis of knockin (KI) mouse mutants for *Mettl16*, with mutations abolishing catalytic activity or RNA binding. A structural model of human METTL16 (PDB: 6GFK) shows the two prolines (PP185–PP186) of the NPPF catalytic motif close to the bound SAH molecule, and a model of human METTL16 in complex with bound *MAT2A* hairpin RNA (PDB: 6DU4) shows the F187 that flips in to interact with the target adenosine upon substrate RNA binding. Introduced mutations are indicated. See also Figures S7A and S7B. Shown are genotypes of animals recovered in born litters from crosses between heterozygous *Mettl16* knockin (KI) parents (*Mettl16*^*KI/+*^). Homozygous KI mutants were not obtained for either mutation, indicating lethality. HET, heterozygous; HOM, homozygous KI. (B) Multiple-tissue western blot showing tissue-specific expression of mouse METTL16. A loading control is provided by detection of PARK7. (C) Representative picture of atrophied testes from a mouse with conditional (*Vasa*-Cre) deletion of *Mettl16* in the germline. Such animals are infertile. See also Figure S7C. (D) Histology of adult mouse testes showing complete absence of germ cells in seminiferous tubules from mice with conditional (*Vasa*-Cre) deletion of *Mettl16* in the germline. cKO, conditional KO. The control HET testis shows all different stages of germ cells, including post-meiotic round spermatids and elongated spermatids. (E) Scheme showing identification of putative targets of mammalian METTL16 on 3′ ss. The total numbers of 3′ ss checked and those recovered with the METTL16/METT-10 motifs are given. The predicted secondary structure of one such RNA (intron-exon boundary with 3′ ss) is shown. (F) *In vitro* methylation assays with recombinant human METTL16 and the indicated RNAs. The RNA sequence for mouse *Mat2a* is from the 3′ UTR, whereas for other mouse genes it spans the intron-exon boundary (sequences are shown below). Reactions were resolved by PAGE, and the radioactivity (^14^C) signal was detected. (G) Two transcripts that show increased splice junction reads specifically in *Mettl16* KO embryos (morulae at E2.5 or blastocysts at E3.5), indicating increased use of that ss in the absence of METTL16. Genomic coordinates of the 3′ ss and the underlying sequence on the Crick strand are shown. *In vitro* methylation assays with RNAs spanning the intron-exon boundary show methylation of the 3′ ss by mammalian METTL16.

Although we demonstrated that splicing inhibition by 3′ splice site m^6^A methylation is conserved in mammals, mammalian METTL16 was not shown to methylate 3′ splice sites of mammalian pre-mRNAs. Mammalian METTL16 regulates its conserved SAM synthetase *MAT2A* RNA target by promoting splicing via its non-catalytic C-terminal VCRs (Pendleton et al., 2017). Loss of *Mettl16* causes pre-implantation embryonic lethality in mice (Mendel et al., 2018). To examine the *in vivo* relevance of its catalytic activity, we created a knockin mouse mutant carrying mutations in the catalytic motif (Figures S7A and S7B; STAR Methods). Although the heterozygous mutants are viable and fertile, homozygous catalytic-dead *Mettl16* mutants are never recovered in the born litters, indicating developmental lethality (Figure 6A). Similarly, mutations designed to cause loss of RNA-binding activity also result in lethality (Figure 6A). METTL16 has a tissue-specific expression pattern in adult mice, with strong enrichment in the gonads (Figure 6B). To probe its relevance for fertility, we engineered conditional deletion of *Mettl16* in the mouse germline (Figure S7C; STAR Methods). Such conditional KO (cKO) males are infertile, as evidenced by atrophied testes (Figure 6C) and arrested germ cell development (Figure 6D). Taken together, our genetic analyses reveal an essential role of the catalytic activity of METTL16 during mouse development and show that the protein is also relevant outside of the embryonic stages.

Next we identified putative mammalian targets for METTL16-mediated 3′ splice site m^6^A methylation (STAR Methods). Briefly, these sites overlap one of the METTL16/METT-10 methylation motifs (UAC**m**^**6**^**A**GAGA or UAC**m**^**6**^**A**GAAA) and are present within a stem-loop structure (Figure 6E). Direct testing of the top 10 such sequences with recombinant human METTL16 shows that several of these are methylated efficiently *in vitro* (Figure 6F). To examine whether any of these putative targets are regulated differentially in the absence of *Mettl16*, we used single-embryo RNA-seq datasets prepared from *Mettl 16* KO embryonic day 2.5 (E2.5) morulae and E3.5 blastocysts (Mendel et al., 2018). This identified *Sorbs1* and *Lrpprc* as two transcripts that have increased use of the 3′ splice site in the *Mettl16* KO (Figure 6G). Furthermore, the target splice sites in these transcripts can be methylated *in vitro* by recombinant METTL16 (Figure 6G). Although our computational and biochemical analyses reveal the existence of putative 3ʹ splice site targets for mammalian METTL16, it remains to be seen whether they are indeed regulated by METTL16 *in vivo*.

## Discussion

SAM is the major methyl donor for methylation reactions in the cell (Cantoni, 1975). Production of SAM from methionine and ATP via the methionine cycle is carried out by methionine adenosyltransferase (MAT) or SAM synthetase, which is conserved from prokaryotes to humans. One conserved principle for regulation of SAM synthetase gene expression is use of RNA structures. Prokaryotes use complex RNA structures, called riboswitches, present in the 5′ leader sequence of SAM synthetase mRNA for feedback regulation by inhibiting translation or attenuating transcription (Batey, 2011; Mandal and Breaker, 2004). Binding of SAM alters the RNA structure, leading to gene repression; for example, by occluding key features like the Shine-Dalgarno sequence required for translation initiation (Breaker, 2018). Even in eukaryotes, fission yeast SAM synthetase *sam1* mRNA has a tertiary structure feature in the 5ʹ UTR, which, upon SAM binding, undergoes structural transition to regulate translation (Zhang et al., 2020).

**Figure 7:**
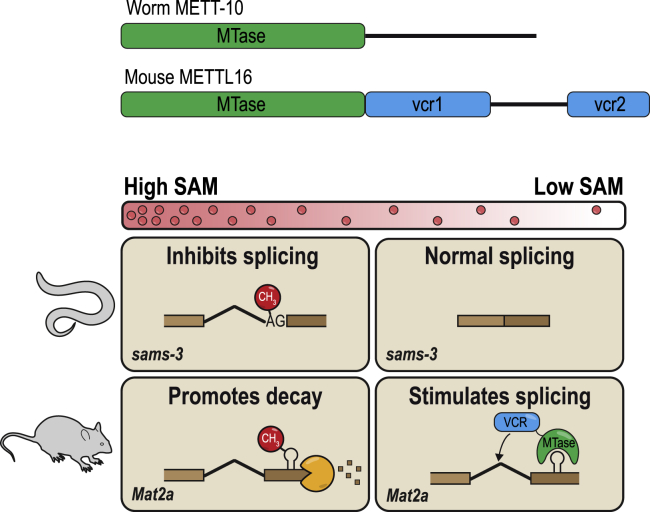
Conserved targets of METTL16-mediated m^6^A methylation activity and specialization of the C-terminal VCR in vertebrates SAM levels are highly regulated *in vivo*, and this is achieved by splicing regulation of the SAM synthetase RNA (*sams-3* or *MAT2A*). Under high-SAM conditions, METT-10 m^6^A methylates a 3ʹ ss in *sams-3* pre-mRNA to directly inhibit splicing, whereas methylation in the 3ʹ UTR of *MAT2A* by mammalian METTL16 leads to intron retention/decay of the RNA. Under low-SAM conditions, mammalian METTL16 binds hairpins in the 3ʹ UTR of *MAT2A* and uses its C-terminal VCR to stimulate splicing of the terminal intron, whereas in nematodes, absence of ss methylation allows normal splicing to proceed. The different mechanisms also highlight the different approaches to regulation of SAM levels: nematode METT-10 turns off SAM production, whereas mammalian METTL16 actively turns on SAM production.

Mammals use a different strategy to regulate *MAT2A* SAM synthetase expression that does not involve direct binding of SAM. Six hairpin structures in the 3′ UTR of the *MAT2A* pre-mRNA bind the m^6^A writer METTL16, which uses its non-catalytic C-terminal VCRs to enhance splicing of a frequently retained terminal intron (Pendleton et al., 2017). The role of SAM in this process is as a molecular regulator of METTL16’s dwell time at the 3′ UTR; low levels increase dwell time, whereas high levels, which lead to m^6^A methylation of the hairpins, rapidly evict the protein from the pre-mRNA (Figure 7). The VCR has also been shown to facilitate binding to the U6 snRNA (Aoyama et al., 2020). In this study, we show that the worm METTL16 ortholog METT-10 inhibits SAM synthetase pre-mRNA splicing via 3′ splice site m^6^A methylation in response to a rich diet. Identification of the precise 3′ splice site is by its presence within a stem-loop structure. Conservation of these sequence elements within SAM synthetase genes implies that this type of regulation might be common in invertebrates (Figures 3E and 3F).

Our findings also highlight the different strategies used to regulate SAM synthetase expression. Prokaryotes and invertebrates negatively regulate SAM production in response to high intracellular SAM levels. On the other hand, mammalian systems have opted for a mechanism that allows them to increase SAM production, probably to suit the requirements of early embryonic development, where optimal SAM levels are critical for survival of embryonic stem cells (ESCs) (Shiraki et al., 2014) and development of embryos (Sun et al., 2019). This critical role explains why mice lacking METTL16 die early during embryogenesis (Mendel et al., 2018), whereas worms lacking METT-10 are viable (Dorsett et al., 2009) because they just need to cope with the aberrantly high levels of SAM after a rich diet (Figure 4E). Nevertheless, the observed phenotypes of the *mett-10* KO and the *sams* triple mutant lacking intron 2 (Figure 4G) demonstrate that the ability to dial down SAM production in response to a rich diet is important for ensuring normal fertility in worms (Figure 4G).

Previous studies have linked m^6^A methylation to splicing regulation, and they document splicing changes in the absence of an m^6^A writer (Haussmann et al., 2016; Lence et al., 2016), reader (Kasowitz et al., 2018; Xiao et al., 2016; Zhou et al., 2019), or eraser (Bartosovic et al., 2017; Zhao et al., 2014). Our study identifies a direct role of the modification in interfering with splicing via precise methylation of a key sequence feature used by the splicing machinery. This mechanism is direct because it repels an essential splicing factor, U2AF35, leading to an early spliceosome assembly defect.

### Limitations of study

The strong fertility defect seen in the *mett-10* KO worms (Dorsett et al., 2009) sharply contrasts the relatively mild phenotype in the triple mutant, where intron 2 is deleted in the *sams-3/4/5* genes (Figure 4G). This points to the existence of additional targets for METT-10 that may contribute to fertility. Alternatively, although we did not observe any global splicing differences (Figure S1J), it is possible that loss of U6 snRNA m^6^A methylation in the *mett-10* KO may affect splicing of specific genes. We show that the 3ʹ splice site m^6^A inhibits splicing in *C. elegans*, and it is also active in human cell cultures and in *in vitro* HeLa splicing extracts, but there is no evidence of its actual use in splicing regulation in mammals. We identified several putative 3ʹ splice sites that are methylated by METTL16 *in vitro* or have, as an isolated RNA sequence, all requirements for methylation. It is possible that many of these sites are never methylated *in vivo* because transcription kinetics (Herzel et al., 2017) may affect the ability of the region to fold into the required stem-loop structure for methylation. Even for the two transcripts (*Sorbs1* and *Lrpprc*) that show altered splicing patterns in the *Mettl16* KO embryos, it is not clear whether this is actually due to m^6^A methylation of the specific 3ʹ splice sites. A search for such mammalian targets will have to involve analysis of specific cell types or tissues under specific developmental or environmental conditions. Nevertheless, given the conservation of the mechanisms involved, our work identifies 3′ splice site methylation as an ancient strategy for splicing control.

## STAR★Methods

### Key resources table

**Table:** 

REAGENT or RESOURCE	SOURCE	IDENTIFIER
**Antibodies**		

Polyclonal rabbit anti-m^6^A	Synaptic Systems	Cat. no. 202003; RRID:AB_2279214
Polyclonal rabbit anti-METT10D (METTL16)	Abcam	Cat. no. ab186012
Polyclonal rabbit anti-PARK7	Invitrogen	Cat. no. PA5-13404, RRID:AB_2160112
Polyclonal rabbit anti-Histone H3	Abcam	Cat. no. ab1791, RRID:AB_302613
Monoclonal mouse anti-FLAG	Sigma	Cat. no. F3165; RRID: AB_259529
Monoclonal rat anti-TUBULIN	Abcam	Cat. no. ab6160, RRID:AB_305328
HRP-conjugated anti-rabbit IgG	GE Healthcare	Cat. no. NA934, RRID:AB_772206
HRP-conjugated anti-mouse IgG	Thermo Fisher	Cat. no. A27025, RRID:AB_2536089
HRP-conjugated anti-rat IgG	GE Healthcare	Cat. no. NA935, RRID:AB_772207

**Bacterial and virus strains**		

BL21(DE3) bacterial strain	NEB	C2527H
DH10EMBacY bacterial strain	Bieniossek et al., 2012	N/A

**Chemicals, peptides, and recombinant proteins**		

γ-^32^P-Adenosine triphosphate	Perkin Elmer	Cat. no. NEG002A001MC
^32^P-Cytidine 3′, 5′ bis(phosphate) [pCp]	Perkin Elmer	Cat. no. NEG019A250UC
^14^C-S-Adenosyl-L-Methionine	Perkin Elmer	Cat. no. NEC363010UC
40% Acrylamide/Bis Solution 19:1	Bio-Rad	Cat. no. 1610144
30% acrylamide (37.5:1)	National Diagnostic	Cat. no. EC-890
N,N,N’,N’-Tetramethylethylendiamin	Merck	Cat. no. 1107320100
Amersham Prime Western Blotting Detection Reagent	GE Healthcare	Cat. no. RPN2232
Pierce ECL Plus Western Blotting Substrate	Thermo Fisher	Cat. no. 32134
Folic acid	Sigma	Cat. no. F8758-5G
Vitamin B12	Sigma	Cat. no. V6629-250MG
L-Methionine	Sigma	Cat. no. M5308-25G
L-Leucine	Sigma	Cat. no. L8912-25G
L-Cysteine	Sigma	Cat. no. C7602-25G
m7G(5′)ppp(5′)A RNA Cap Structure Analog	NEB	Cat. no. S1405S
Pierce Protease Inhibitor Tablets, EDTA-free	Thermo Fisher	Cat. no. A32965
Benzonase Nuclease	Santa Cruz Biotechnology	Cat. no. sc-202391
Nuclease P1	Sigma	Cat. no. N8630
RiboLock RNase Inhibitor	Thermo Fisher	Cat. no. EO0381
Chloroform	Merck	Cat. no. 102445
RNase H	Thermo Fisher	Cat. no. EN0201
FastAP	Thermo Fisher	Cat. no. EF0651
T4 PNK	NEB	Cat. no. M0201L
10 mM ATP	GE Healthcare	Cat. no. 27-2056-01
100% DMSO	Thermo Fisher	Cat. no. F-515
T4 DNA Ligase	NEB	Cat. no. M0202M
RNase T1	Thermo Fisher	Cat. no. EN0541
RNase A	Sigma	Cat. no. R6513
T4 RNA Ligase 1	NEB	Cat. no. M0204
Water-saturated phenol	AppliChem	Cat. no. A1624
Glycogen, RNA grade	Thermo Fisher	Cat. no. R0551
RiboRuler Low Range RNA Ladder	Thermo Fisher	Cat. no. SM1831
Bouin’s solution	Sigma	Cat. no. HT10132

**Critical commercial assays/kits**		

NEBNext Multiplex Small RNA Library Prep Set for Illumina	NEB	Cat. no. E7300
MinElute Gel Extraction Kit	QIAGEN	Cat. no. 28604
MEGAshortscript T7 Transcription Kit	Life Technologies	Cat. no. AM1354
Dynabeads Protein A	Life Technologies	Cat. no. 10002D
Dynabeads mRNA purification kit	Life Technologies	Cat. no. 61006
Maxima H Minus First Strand cDNA kit	Thermo Fisher	Cat. no. K1682
Phire Green Hot Start II PCR Master Mix	Thermo Fisher	Cat. no. F126L
DC Protein Assay Kit II	Bio-Rad	Cat. no. 5000112

**Deposited data**		

Deep sequencing datasets	Mendel et al., 2018	GEO: GSE116329
Deep sequencing datasets	This study.	GEO: GSE146873
All raw gel data are deposited at Mendeley Data.	This study.	https://doi.org/10.17632/s92zgtbhjp.1

**Experimental models: Cell lines**		

Sf21 insect cells for protein production	Eukaryotic Expression Facility, EMBL Grenoble, France	N/A
High Five (Hi5) insect cells for protein production	Eukaryotic Expression Facility, EMBL Grenoble, France	N/A
HeLa cells	ECACC	Cat. no. 93021013
HeLa S3 cells	ECACC	Cat. no. 87110901
*Bombyx* cell line (BmN4-SID1)	(Mon et al., 2012)	RRID:CVCL_Z091

**Experimental models: Organisms/strains**		

Mouse: *Mettl16 knock-out*	Mendel et al., 2018	EMMA (EM: 12199)
Mouse: *Mettl16 F187G mutation*	This study	Available from Lead Contact
Mouse: *Mettl16 185PP→AA186 mutation*	This study	Available from Lead Contact
Mouse: *Mettl16 Floxed*	KOMP repository	*Mettl16*^tm1a(KOMP)Mbp^
Mouse: *Ddx4-Cre*	The Jackson Laboratory	Cat. no. 006954, RRID:IMSR_JAX:006954
*C. elegans*: WT (N2 Bristol strain)	Caenorhabditis Genetics Center	
*C. elegans: mett-10* KO Genotype: ZK1128.2(*ok2204*) III.	Caenorhabditis Genetics Center	Strain VC1743 WormBase: WBStrain00036838
C. elegans strains generated in the study	This study	See Table S5

**Oligonucleotides**		

DNA and RNA oligos	This study	See Table S3

**Recombinant DNA**		

pACEBac2	Bieniossek et al., 2012	N/A
Human *Mettl16* cDNA	Mendel et al., 2018	NP_076991; NM_024086
Worm *mett-10* cDNA	This study	NP_499247.2, NM_066846.4
Worm *sams-3* full-length gene	This study	Gene ID: 177355
*S. pombe* U2AF35 cDNA	This study	NP_594945.1, NM_001020376.2
*S. pombe* U2AF65 cDNA	This study	NP_595396.1, NM_001021303.2

**Software and algorithms**		

Cutadapt		https://doi.org/10.14806/ej.17.1.200
MEME - Motif discovery tool	Bailey and Elkan, 1994	https://meme-suite.org/meme/
WebLogo		http://weblogo.berkeley.edu/
R	R Core Team, 2017	https://www.r-project.org
Bowtie	Langmead et al., 2009	http://bowtie-bio.sourceforge.net/
DESeq2	Love et al., 2014	https://bioconductor.org/packages/DESeq2
Bioconductor	Huber et al., 2015	https://www.bioconductor.org/
Salmon	Patro et al., 2017	https://combine-lab.github.io/salmon/
MACS2	Zhang et al., 2008	https://github.com/macs3-project/MACS
MSPC	Jalili et al., 2018	https://genometric.github.io/MSPC/
BLAST	Altschul et al., 1990	http://blast.ncbi.nlm.nih.gov//blast.ncbi.nlm.nih.gov/Blast.cgi
RNAfold	Lorenz et al., 2011	https://www.tbi.univie.ac.at/RNA/
STAR	Dobin et al., 2013	https://github.com/alexdobin/STAR

**Other**		

Chelating Sepharose Fast Flow beads	GE Healthcare	Cat. no. 17-0575-01
StrepTrap HP	GE Healthcare	Cat. no. 28-9075-46
Superdex S75 10/300 GL	GE Healthcare	Cat. no. 17-5174-01
Superdex 200 10/300 GL	GE Healthcare	Cat. no. 17-5175-01
MethaPhor agarose	Lonza	Cat. no. 50180
Amersham Protran 0.45 mm Nitrocellulose Membrane	GE Healthcare	Cat. no. 10600002
Amersham MicroSpin S-400 HR Columns	GE Healthcare	Cat. no. GE27-5140-01
TRIzol Reagent	Invitrogen	Cat. no. 15596026
TLC PEI Cellulose F plates	Merck	Cat. no. 1055790001
Phosphor Screen BAS IP MS 2025 E	GE Healthcare	Cat. no. 28956475
Peel-A-Way Embedding Mold S22	Polysciences	Cat. no. 18646A-1
Superfrost Plus microscope slides	Thermo Fisher	Cat. no. 4951PLUS4

### Resource availability

#### Lead contact

Further information and requests for resources and reagents should be directed to and will be fulfilled by the Lead Contact Ramesh S. Pillai (ramesh.pillai@unige.ch).

#### Materials availability

All unique reagents including plasmids, animal models etc generated in this study are available from the Lead Contact without any restriction.

#### Data and code availability

The accession number for the deep sequencing data reported in this paper is GEO: GSE146873. Other deep sequencing data used (GEO: GSE116329) are already published. Original data have been deposited to Mendeley Data: https://doi.org/10.17632/s92zgtbhjp.1. Code used in the current study is available from the corresponding authors upon reasonable request.

### Experimental model and subject details

#### Animal work

Mutant mice were generated at the Transgenic Mouse Facility of University of Geneva or obtained from the Knockout Mouse Project (KOMP). The mice were bred in the Animal Facility of Sciences III, University of Geneva. The use of animals in research at the University of Geneva is regulated by the Animal Welfare Federal Law (LPA 2005), the Animal Welfare Ordinance (OPAn 2008) and the Animal Experimentation Ordinance (OEXA 2010). The Swiss legislation respects the Directive 2010/63/EU of the European Union. Any project involving animals has to be approved by the Direction Générale de la Santé and the official ethics committee of the Canton of Geneva, performing a harm-benefit analysis of the project. Animals are treated with respect based on the 3Rs principle in the animal care facility of the University of Geneva. We use the lowest number of animals needed to conduct our specific research project. Discomfort, distress, pain and injury is limited to what is indispensable and anesthesia and analgesia is provided when necessary. Daily care and maintenance are ensured by fully trained and certified staff. All animals were housed 3-5 per cage and maintained on a 12-hour light/dark cycle, with water and food available *ad libitum*. The use of mice in this work was approved by the Canton of Geneva (GE/162/19, GE/16/19 and GE13).

#### Generation of catalytic-dead and RNA-binding mutant Mettl16 mouse lines

*Mettl16* gene locus is located in mouse on chromosome 10 and consists of 10 exons (Figure 7A). The locus was modified (Transgenic Core Facility of the University of Geneva) using CRISPR/Cas9 technology to introduce mutations meant to destroy the RNA methylation activity (F187G) and RNA-binding activity (185PP-AA186) of METTL16 (Mendel et al., 2018). Mouse embryos of the B6D2F1/J hybrid line (also called B6D2; The Jackson Laboratory, stock no. 100006) were used. This line is a cross between C57BL/6J (B6) and DBA/2J (D2) and is heterozygous for all B6 and D2 alleles. Single-cell mouse embryos were injected with a single guide RNA (gRNA) and a 200 nucleotides long single-stranded DNA (ssDNA) repair template (IDT, Belgium). Sequences of the gRNA and ssDNA repair templates are provided (Table S3). Template for F187G mutation had a mutation CCTCCCTTT to CCTCCCGGC, while the template for 185PP-AA186 mutation had CCTCCCTTT to GCCGCATTT (Figure 7A).

Founder male mice were crossed with wild-type C57BL/6J (Janvier) female partners to obtain germline transmission. Heterozygous male and female animals from the F1 generation were crossed with each other to obtain homozygotes. To genotype the animals, we PCR-amplified the region around the mutations and sequenced the PCR products (Figure S7B). While male and female heterozygotes for both mutations were detected, there were no homozygotes at weaning age (P21), pointing to potential embryonic lethality due to loss of either catalytic activity or loss of RNA-binding ability of METTL16 (Figure 6A).

#### Conditional Mettl16 knockout mouse generation

*Mettl16*^*tm1a(KOMP)Mbp*^ mouse was obtained from the Knockout Mouse Project (KOMP; https://www.komp.org/) repository at University of California, Davis, USA. *Mettl16*^*tm1a(KOMP)Mbp*^ mouse has the L1L2_Bact_P gene-trapping cassette inserted between exon 6 and exon 9 of the *Mettl16* gene (Figure S7C). This cassette has multiple functionalities. By default, it functions as a gene-trap as it has a LacZ ORF with a polyadenylation signal that is preceded by a splice acceptor site. Thus, the upstream exons of *Mettl16* pre-mRNA will become spliced to the LacZ sequence, and the polyadenylation signal will ensure that transcription terminates prematurely on the *Mettl16* locus. In addition, the cassette also brings two *loxP* sites flanking exons 7 and 8 of *Mettl16*, allowing conditional knockout (cKO) of the gene.

To allow for the conditional knockout of *Mettl16* gene, the FRT-flanked gene-trap cassette is removed by crosses with a mouse line expressing FLP recombinase from the ubiquitous ROSA26 promoter [B6.129S4-Gt(ROSA)26Sor^*tm1(FLP1)Dym*^/RainJ, The Jackson Laboratory]. Gene-trap cassette removal leaves behind only the *loxP* sites flanking exons 7 and 8, creating the male and female heterozygous floxed *Mettl16*^*loxP/+*^ mice (Figure S7C). The *Mettl16*^*loxP/+*^ male and female mice were crossed with each other to obtain homozygous male and female floxed *Mettl16*^*loxP/loxP*^ mice. In order to delete *Mettl16* in the germline, a mouse line expressing Cre recombinase under germline-specific promoter (*Ddx4*) was obtained: FVB-Tg(Ddx4-cre)1Dcas/J (The Jackson Laboratory). This line (males or females) was first crossed with male or female *Mettl16*^*+/−*^ heterozygous knockout animals carrying a null allele (Mendel et al., 2018), producing the male and female *Mettl16*^*-/+*^*;Ddx4*-Cre animals. These male or female animals were next crossed with the male or female *Mettl16*^*loxP/loxP*^ line, generating male and female animals with conditional knockout (cKO, *Mettl16*^*loxP/-*^*;Ddx4-Cre*) of the floxed *Mettl16* allele in the germline and male and female *Mettl16* heterozygotes (*Mettl16*^*loxP/+-*^*;Ddx4-Cre*). The germline-specific expression of *Ddx4* starts approximately at embryonic day 16.5 (E16.5) in male embryos, leading to early deletion of *Mettl16* in the testes. Such cKO males were found to be infertile due to an early block in spermatogenesis and had atrophied testes (Figures 6C and 6D). The conditional deletion of *Mettl16* is also expected to take place in the female germline, but we did not examine impact on fertility in such cKO females.

#### Genotyping

Ear punches of weaned male and female animals (21 days-old) were digested for 120 min at 95°C in 100 μl of buffer containing 10 mM NaOH and 0.1 mM EDTA. After centrifugation at 3000 rpm for 10 min, 50 μL of supernatant was transferred to a new tube containing 50 μL of TE buffer (20mM Tris-HCl, pH 8.0 and 0.1 mM EDTA). 1.5 μl of digestion mix was used for PCR with Phire Greeen Hot Start II PCR Mix (F126L, Thermo Fisher). The annealing temperatures were calculated using Tm calculator (Thermo Fisher). Reactions were examined by 2% agarose gel (Promega, cat.no. V3125) electrophoresis (Figure S7C).

For genotyping the male and female conditional *Mettl16* knockout mice, the primers sequences are provided (Table S3), so is a representative gel showing the PCR products (Figure S7C). For genotyping the *Mettl16*^*loxP/loxP*^ mice primers MM101 and MM102 were used (Table S3) to detect loxP inserted into the region (WT PCR product: 474 bp, loxP PCR: 439 bp). To genotype conditional *Mettl16* knockout mice (*Mettl16*^*loxP/-*^*;Ddx4-Cre)* primers (oIMR7643, oIMR7644) detecting *Ddx4-Cre* (PCR product size: 240 bp) as well as detecting *Mettl16*^*+/−*^ (MM314, MM315; PCR product size: 296 bp) were used. Representative gels showing the PCR products are shown (Figure S7C).

For genotyping the male and female point mutant *Mettl16* mice, a region of 308 bp around the mutation site was amplified using MM340 and MM341 primers (Table S3) and Phire Green Hit Start II polymerase producing a 308 bp PCR product. PCR conditions: 98°C for 1 min., 30 x (98°C for 5 s, 65°C for 10 s., 72°C for 15 s.), 72°C for 1 min., 4°C hold. PCR products were purified with QIAquick® PCR Purification Kit (cat.no. 28106, QIAGEN) and sent for Sanger sequencing (Fasteris SA, Geneva) (Figure S7B). Having confirmed the existence of the mutations in the genome, we then designed primers that allow routine genotyping by specific detection of the mutations by genomic PCR. To this end, primers MM342 and MM343 (269 bp PCR product) were used to detect F187G mutation, primers MM348 and MM349 (265 bp PCR product) to detect PP185-186AA mutation (Table S3). The PCR reaction conditions were identical for both mutations: 98°C for 1 min., 30 x (98°C for 5 s, 65°C for 10 s., 72°C for 15 s.), 72°C for 1 min., 4°C hold.

#### Nematode strains and growth conditions

*C. elegans* strains were grown under the standard OP50 conditions for maintenance (Brenner, 1974). For experiments, the worms were fed one of the two diets: nutrient-low media (*E. coli* bacterial strain OP50 on NGM plates) or nutrient-high media (*E. coli* strain NA22 on peptone-rich plates), as detailed (Table S4) and indicated for each experiment. N2 (Bristol strain) was used as wild-type, unless otherwise indicated. The list of strains used in this study can be found in Table S5. All of the *C. elegans* experiments and worm maintenance was carried out at 20°C.

For culture conditions were the nutrient-low media was supplemented with various components, the nutrient-low OP50 plates were prepared with additional methionine (Sigma, cat. no. M5308), leucine (Sigma, cat. no. L8912) or cysteine (Sigma, cat. no. C7602) (with the final concentration of 10 mM for each aminoacid), with folic acid (Sigma, cat. no. F8758) (100 μM concentration) or vitamin B12 (Sigma, cat. no. V6629) (73 nM concentration). The required additional components were added to the cooled autoclaved media, just before pouring plates.

#### Generation of *C. elegans* strains

All of the genome editing for strain creation was performed in the endogenous loci of *sams-3, sams-4, sams-5* and *mett-10* genes using CRISPR/Cas-9 technology as described in Arribere et al. (2014). In brief, Cas-9 and sgRNAs in the form of plasmids and repair templates as single-stranded oligonucleotides were delivered to the worm germ cells through microinjection into the gonad. Sequences of sgRNAs, repair templates and plasmids used to detect and sequence the edits are indicated in the Table S3. SAMS-3 was tagged with 2xHA on C terminus, METT-10 was tagged with 3xFLAG and 1xHA on the C terminus, intron 2 was removed from *sams-3*, *sams-4* and *sams-5* (Figure 4F). All of the edits were performed on the endogenous copy of the genes. For intron 2 deletions, multiple alleles were generated in different genetic backgrounds (Figure 4G).

#### Generation of *C. elegans* lines expressing transgene reporter constructs as transgenes

For the transgene reporter constructs experiments (Figures 3D and S3A), either the wild-type N2 (Bristol strain) or the *mett-10* knockout VC1743 strain was used for plasmid injections. Generation of extrachromosomal arrays was carried out via microinjection as described in WormBook” (Evans, 2006) Briefly, the plasmids containing WT or MUT transgene reporter constructs were co-injected along with reporter plasmids pRF4 [*rol-6*(*su1006*), causes roller phenotype due to a cuticle defect] and pCFJ421 [Pmyo-2::GFP::H2B (pharynx)] into the gonads of young adult wild-type or *mett-10* knockout worms. Concentrations of plasmid injected are 5 ng/μl (transgene reporter constructs), 2 ng/μl (pRF4) and 5 ng/μl (pCFJ421). Progenies displaying phenotypes induced by presence of reporter plasmids (rollers with strong pharyngeal GFP signal) were singled out. The constructs are expected to be maintained as extrachromosomal arrays. In the following generation, 6 lines showing the highest rate of array transmission were identified and the presence of WT or MUT plasmid was confirmed by RT-PCR using MM363 and MM364 primers (Table S3). The transgenic lines were maintained by picking rollers. For the splicing analysis, we used three independent lines for the triplicate repetitions. For each repetition, 30 rollers were picked into 100 ul of TRIzol (Thermo Fisher, cat.no. 15596026).

#### Collection of *C. elegans* for the RNA isolation

For analysis of wild-type and *mett-10* knock-out (VC1743) worms, synchronized adult population was washed off either from 15 cm plates (in case of NA22) or from 6 cm plate (in case of OP50). Worms were washed 3 times in M9 buffer (3 g KH_2_PO_4_, 6 g Na_2_HPO_4_, 5 g NaCl, 1 mL 1 M MgSO_4_ in 1l H_2_O), put in Trizol (3x volume of the worm pellet), flash-frozen in liquid nitrogen and stored at −80°C until the RNA isolation.

#### Cell lines

Cell lines were obtained from the European Collection of Authenticated Cell Cultures (ECACC). HeLa cells (ECACC, cat. no. 93021013) were grown in Dulbecco’s modified Eagle Medium (DMEM; Invitrogen, cat. no. 21969-035) supplemented with 10% fetal bovine serum (Thermo Fisher; cat. no. 10270106), 1% Penicilline/Streptomycin (Thermo Fisher; cat. no. 15140122), 2 mM L-Glutamine (Thermo Fisher; cat. no. 25030024). HeLa S3 (ECACC, cat. no. 87110901) were grown in spinner flasks (Corning, cat. No. 4500-125) in MEM medium supplemented with 2 mM L-Glutamine, 1% Non Essential Amino Acids (NEAA) (Thermo Fisher, cat. no. 11140050), 1% Penicilline/Streptomycin and 10% fetal bovine serum (Thermo Fisher). Both cell types were maintained in an environment with 5% CO_2_ at 37°C. HeLa cells were sub-cultured at 1:5 ratio every 3 to 4 days using 0.05% trypsin-EDTA (GIBCO, cat. no. 25300054), while HeLa S3 were counted daily using Neubauer chamber and maintained at the 200 000 – 500 000 cells/mL concentration by diluting the culture with growth media.

### Method details

#### Clones and constructs

The *C. elegans* METT-10 (Wormbase: CE31860), was cloned by RT-PCR amplification from adult worm total RNA. The *sams-3* SAM synthetase gene (Wormbase: CE03957) was PCR amplified from worm genomic DNA. The cDNAs for yeast (*S. pombe*) U2AF35 (U2AF23, UniProtKB/Swiss-Prot: Q09176.2) and yU2AF65 (U2AF59, UniProtKB/Swiss-Prot: P36629.1) were synthesized (Thermo Fisher).

#### Constructs for bacterial protein expression

The untagged full-length yeast (*S. pombe*) U2AF35 (U2AF23) and a tagged (6xHis-StrepII) fragment (93-161 aa) of yU2AF65 (U2AF59) were co-expressed in *E. coli* and purified as a complex. This minimal fragment of yU2AF65 (93-161 aa) is essential and sufficient for ensuring stability of yU2AF35 (Yoshida et al., 2015). The required ORFs were cloned into the pETDuet-1 vector (Novagen) for co-expression in *E. coli*. We also prepared complexes where the yU2AF35 has specific point mutations in the zinc finger 1 (Figures S6B–S6D). The Arginine 35 in ZF1 is proposed to be involved in recognition of the splice site adenosine (Yoshida et al., 2015). A conservative mutation to a positively-charged lysine (R35K) or to a non-conservative mutation to uncharged serine (R35S) were made. The logic of R35K mutation was to see if the shorter side-chain of Lysine could allow recognition of m^6^A. We also made a mutation (S34Y) in the serine 34, which is frequently mutated in patients with myelodysplastic syndromes (Yoshida et al., 2011). The constructs used for recombinant protein production were verified by restriction digest, as well as by Sanger sequencing.

#### Constructs for insect cell expression

The worm METT-10 ORF was cloned into pACEBac2-His-StrepII-SUMO vector (Geneva Biotech) for expression in Sf21 or High Five (Hi5) insect cells as a 6xHis-StrepII-SUMO-tagged fusion. The constructs used for recombinant protein production were verified by restriction digest, as well as by Sanger sequencing. Expression construct for human METTL16 (hMETTL16) was previously reported (Mendel et al., 2018).

#### Constructs for expression of *sams-3* transgene reporters in transgenic worms

Transgene reporter constructs were based on modified full-length *C. elegans* SAM synthetase gene *sams-3* (Wormbase: CE03957). The whole *sams-3* gene (2189 nt, including 5′ and 3′UTR sequences) was amplified using DH298 and DH299 primers, but to distinguish the transgene reporter constructs from the endogenous *sams-3* transcript, 20 nt-long artificial sequences were placed into the exon2 (TGAACGACCGTGTTCTAGGG, DH300 and DH301) and exon3 (ACAGCCTACTTTGAGTGCGTA, DH302 and DH303), allowing for a specific PCR amplification (Table S3). The inserted 20-nt artificial sequences also rendered the reporters non-coding, as they were designed to cause a frameshift in the protein coding sequence. In addition, the wild-type (WT) METT-10 methylation consensus motif (UAC**AG**AAAC) that overlaps the 3′ splice site AG was mutated (MUT) in the part of the consensus that belongs to the exon3 (UAC**AG**ACUU, mutation is underlined). The mutation was introduced using MM320 and MM321 primers, and by amplifying the whole plasmid (Table S3). Such a mutation is demonstrated to prevent methylation by METT-10 *in vitro* (Figure 3B). Making the mutations on the exonic part is meant to reduce the disruption of binding sites for the key splicing factors. The constructs were cloned into the pUC19 plasmid for *C. elegans* expression under the *his-72* promoter to ensure ubiquitous expression of the construct. The constructs were verified by restriction digest, as well as by Sanger sequencing.

#### Constructs for expression of transgene reporters in human cells

*Sams-3* full-length fragment was PCR-amplified from the pUC19-sams-3 plasmid prepared for worms injections, using MM415 and MM416 primers (Table S3). Forward primer introduced NotI restriction site, while reverse primer introduced NheI site. The amplified fragment was cloned into the mammalian expression vector phRL-TK (Promega), which was first digested with NotI-HF (NEB, cat. no. R3189S) and NheI-HF (NEB, cat. no. R3131S) restriction enzymes. This removes the whole luciferase (hRL) sequence. The phRL-|TK vector has the HSV Thymidine Kinase promoter, allowing low-level expression in mammalian cells. The final constructs used were verified by restriction digest, as well as by Sanger sequencing.

#### Antibodies

The polyclonal rabbit anti-m^6^A (Synaptic Systems; cat. no. 202003) for m^6^A-IP-seq, polyclonal rabbit anti-METTL16 (abcam; cat. no. ab186012) to detect mouse METTL16, polyclonal rabbit anti-PARK7 (Invitrogen, cat. no. PA5-13404) to detect mammalian PARK7 as a normalization control for western blots, anti-HA (a kind gift from Marc Bühler, clone #42F13) to detect HA-tag, anti-FLAG (Sigma, cat. no. F3165) to detect FLAG-tag, anti-TUBULIN (Abcam; cat. no. ab6160) to detect worm TUBULIN, and anti-H3 (Abcam; ab1791) to detect worm histone H3 were used. For secondary antibodies, the HRP-conjugated anti-rabbit IgG HRP-linked (GE Healthcare; cat. no. NA934), HRP-conjugated anti-mouse IgG (H+L) Superclonal Secondary Antibody (Thermo Fisher, cat. no. A27025) or HRP-conjugated anti-rat IgG (GE Healthcare, cat. no. NA935) were used.

#### Recombinant protein production

Production of full-length recombinant proteins was carried out either in insect cell lines using the baculovirus expression system or in the prokaryotic expression system using *E. coli*.

The insect ovary-derived cell lines used are: High Five (Hi5) insect cell line originating from the cabbage looper (*Trichoplusia ni*) and the Sf9 cells derived from the fall army worm *Spodoptera frugiperda*. Briefly, recombinant full-length hMETTL16 (Mendel et al., 2018) or worm METT-10 coding sequences were cloned into the pACEBac2-Sumo acceptor vector (His-Strep-Sumo tag) (Bieniossek et al., 2012). Plasmids were transformed into DH10EMBacY competent cells for recombination with the baculovirus genomic DNA (bacmid). The bacmid DNA was extracted and transfected with FuGENE HD (Promega, cat. no. E231A) into the *Sf*9 insect cells for virus production. The supernatant (V_0_) containing the recombinant baculovirus was collected after 72 to 96 hours post-transfection. To expand the virus pool, 6.0 mL of the V_0_ virus stock was added into 25 mL of *Sf*9 (0.5 × 10^6^/mL) cells. The resulting cell culture supernatant (V_1_) was collected 24 h post-proliferation arrest. For large-scale expression of the protein, Hi5 cells were infected with virus (V_1_) and cells were harvested 72 h post-proliferation arrest.

For bacterial expression, plasmids were transformed into the *E. coli* BL21(DE3) strain and expression was initiated by addition of 0.7 mM Isopropyl β-D-1-thiogalactopyranoside (IPTG) when the culture density reached 0.6 (OD_600_). The proteins were then expressed overnight at 20°C following induction.

#### Expression and purification of yeast U2AF35 protein

The U2AF heterodimer is formed by interactions between the large subunit U2AF65 and small subunit U2AF35 (Zamore and Green, 1989). The U2AF65 has three RNA recognition motifs (RRMs), while the U2AF35 has one RRM flanked by two CCCH-type zinc fingers (ZFs). Only a 28-amino acid fragment from U2AF65 is required for interaction with U2AF35 (Kielkopf et al., 2001). A soluble *S. pombe* U2AF complex consisting of full-length U2AF35 and a larger region (93-161 aa) encompassing the 28-amino acid proline-rich fragment from U2AF65, was previously described (Yoshida et al., 2015). This complex is shown to specifically recognize the AG dinucleotide, as single mutations in the RNA at these positions either abolish or greatly reduce binding to the RNA (Yoshida et al., 2015). Importantly, this complex does not bind a polypyrimidine stretch (U_10_) (Yoshida et al., 2015). The ZFs in U2AF35 cooperatively bind the RNA (Yoshida et al., 2015).

We used this *S. pombe* U2AF complex (which has RNA-binding property only in U2AF35) for our studies, and for simplicity is referred to as full-length yeast U2AF35.

The ORFs for yU2AF35 and His-StrepII tag fused yU2AF65 (93-161 aa) were cloned into pETDuet-1 vector, and the plasmid was transformed into *E. coli* BL21 (DE3) strain for co-expression. The protein complex was expressed overnight at 20°C with 0.5 mM IPTG. Cells were harvested and lysed by sonication in lysis buffer (30 mM Tris-HCl, pH 8.0, 200 mM NaCl, 5 mM 2-mercaptoethanol, 5% Glycerol, 20 mM Imidazole and proteinase inhibitor). The supernatant after centrifugation was subjected to Ni-NTA column and StrepTrap column for obtaining His-StrepII tagged protein complex. After removal of the tags by overnight TEV cleavage, the untagged protein complex was further purified by size exclusion column (Superdex S75) in buffer (20 mM HEPES, pH 7.0, 100 mM NaCl).

#### ITC experiment with yeast U2AF35

ITC experiments were performed (at EMBL Grenoble, France; kind help of Dr. Andrew McCarthy) using a MicroCal iTC 200 (Malvern Panalytical) at 20°C. The yeast U2AF35 protein, as well as RNAs, were dialyzed overnight in the buffer (20 mM HEPES, pH 7.0, 100 mM NaCl). The sample cell was filled with 50 μM of either unmethylated or m^6^A methylated RNA (CUAGG, methylated adenosine is underlined), and the syringe was filled with 500 μM yeast U2AF protein. Titrations were carried out with a first 0.4 μL injection followed by constant volume injections (19 injections of 2 μL) with 150 s spacing. Data analysis was performed using Origin software.

#### Worm total RNA purification

Worms were collected in TRIzol as described in the “Collection of C. elegans for the RNA isolation” section. To isolate RNA, worms were first kept in TRIzol at room temperature for 1 – 2 hours with frequent vortexing to ensure complete lysis. Then, samples were spun for 10 min. at 14000 x g at 4°C and supernatant was transferred to fresh tubes. Chloroform (Merck, cat. no. 102445) was added to TRIzol in 1 to 5 volume ratio and the tubes were first vortexed for 15 s. and then left at room temperature for 3 minutes. Next, the tubes were spun at 14000 x g for 15 min. at 4°C. The upper layer (aqueous phase) was transferred to fresh tubes and an equal amount of chloroform was added. The tubes were vortexed for 30 s. and spun at 14000 x g for 10 min. at 4°C. The upper layer (aqueous fraction) was transferred to fresh tubes, where 2.5 volume of 100% ethanol (VWR, cat. no. 20821.321) was added. The tubes were stored at −20°C for at least 1 hour to precipitate the RNA. After precipitation, the tubes were spun at 14000 x g for 30 min. at 4°C. The RNA pellet was washed once with 70% ethanol, dried at RT and resuspended in RNase-free water (Invitrogen, cat. no. 10977-05). The isolated RNA was stored at −80°C to avoid RNA degradation.

#### Poly(A)^+^ RNA purification

Worm poly(A)^+^ RNA was purified using Dynabeads mRNA Purification Kit (Invitrogen, cat. no. 61006). All the step were performed accordingly to the protocol provided by the manufacturer. Briefly, 225 μg of RNA was diluted in RNase-free water to a final volume of 300 μl. RNA was then heated to 65°C for 3 minutes and placed on ice. At the same time, 600 μl of resuspended Dynabeads Oligo (dT)_25_ beads were transferred to fresh tubes, which were placed on a magnetic stand. The supernatant was removed and 300 μl of Binding Buffer was added to equilibrate the beads. Tubes were again placed on a magnetic stand and supernatant was removed. Another 300 μl of Binding Buffer was added and the beads were mixed with the RNA (1:1 volume ratio). The RNA was incubated with the beads for 15 min. at RT with rocking. Then, tubes were placed at the magnetic stand and supernatant was removed. Beads were washed twice with 600 μl Washing Buffer B. Next, beads were resuspended in 40 μl of water, heated to 75°C for 2 minutes and immediately placed on ice. The supernatant with eluted mRNA was transferred into fresh tubes and stored at −80°C to avoid RNA degradation.

#### Detection of m^6^A methylation using SCARLET

We followed the method described previously (Liu et al., 2013) to produce the data presented in Figures 1J and S1H. The oligonucleotides used are provided in Table S3. In short, 1 μg of total RNA or mRNA, isolated as previously described, was mixed with 3 pmol of chimeric oligo in a total volume of 3 μl of 30 mM Tris-HCl, pH 7.5. We tested different lengths (17/18 nt, 20 nt, 23 nt, 30 nt) of chimeric oligos for both U6 snRNA and *sams-3* targets. Generally, the shortest chimeric oligos (18 nt) were performing the best, although in the case of U6 snRNA the difference wasn’t dramatic. On the other hand, in the case of the *sams-3* target, there was a huge increase in cleavage efficiency from 20 nt to 18 nt chimeric oligo. Thus, we recommend using short chimeric oligos and testing different sizes.

The mix was heated for 1 min. at 95°C followed by incubation at room temperature for 3 min. before placing on ice. Then, 1 μl of 5x RNase H mix (2 x T4 polynucleotide kinase buffer (NEB, cat. no. B0201S), 1 unit/μl of RNase H (Thermo Fisher, cat. no. EN0201)) and 1μl of FastAP (Thermosensitive Alkaline Phosphatase, 1U/μl, Thermo Scientific, cat. no. EF0651) were added to the tube (total volume was 5 μl). Samples were incubated for 1 hour at 44°C and then heated for 5 min. to 75°C in order to inactivate RNase H and FastAP. Immediately after the heating, samples were placed on ice. We have tested multiple RNase H enzymes: NEB, cat. no. M0297S; Thermo Scientific, cat. no. EN0201; Sigma, cat. no. R6501; Invitrogen, cat. no. 18021014; Takara, cat. no. 2150A. Although all of them worked, RNase H from Thermo Scientific (cat. no. EN0201) and RNase H from Sigma (cat. no. R6501) seemed to be the most efficient in our hands. After the RNase H digest, the RNA was 5ʹ-end labeled with ^32^P by adding 1 μl of 6 x T4 PNK mix (1 x T4 PNK buffer, 6 units/μL of T4 PNK (NEB, cat. no. M0201L) and 28 μCi/μL [γ-^32^P] ATP (PerkinElmer, NEG002A001MC)). The mix was incubated for 1 hour at 37°C, then heated for 5 min. at 75°C in order to inactivate T4 PNK and immediately put on ice.

We took 1.5 μl mix of 4 pmol of splint oligo/5 pmol of ssDNA universal oligo (ssDNA-116/MM437) was added to the tubes. The mix was annealed by heating for 3 min. at 75°C and cooling down for 3 min. at room temperature, then it was put on ice. 2.5 μL of 4 x ligation mix (1.4x T4 PNK buffer, 0.27 mM ATP (GE Healthcare, cat. no. 27-2056-01), 57% DMSO (ThermoScientific, F-515), 80 U/μL T4 DNA ligase (NEB, M0202M)) was added and RNA was ligated for 3.5 hours at 37°C. The reaction was stopped with 2 x RNA loading buffer (9 M urea, 100 mM EDTA, xylene and bromophenol dye) and 1 μL of RNase T1/A mix (160 U/μL RNase T1 (ThermoScientific, cat. no. EN0541) and 0.16 mg/mL RNase A (Sigma, cat. no. R6513-10MG)) was added. The RNA was incubated overnight at 37°C. Next, it was run on 10% Urea-PAGE gel together with a ^32^P labeled ssDNA universal oligo (ssDNA-116/MM437) used as a marker. The band corresponding to 117-/118-bp was cut out of the gel. RNA was eluted from the gel for 4 h at 25°C with 750 rpm shaking using RNA extraction buffer (300 mM sodium acetate, 1 mM EDTA, 0.1% SDS). Supernatant was transferred to a fresh tube and RNA was isolated with phenol/chloroform. RNA pellet was then resuspended in 3 μl of Nuclease P mix (0.33 U/μL of Nuclease P1 (Sigma, cat. no. N8630) in 30 mM sodium acetate pH 4.8) and incubated for 2 h at 37°C.

After digestion, 1 μL of digested RNA was transferred into TLC PEI Cellulose F plate (Merck, cat. no. 1055790001) and was resolved for 14 h in a mix of isopropanol:HCl:water (70:15:15, v/v/v). After that, the TLC plate was dried at RT for 30 – 60 min., wrapped in a plastic film and exposed to a phosphor screen BAS IP MS 2025 E (GE Healthcare, cat. no. 28956475). Phosphor screen was scanned in a Typhoon FLA 9500 laser scanner (GE Healthcare) at 700V and 100 μm pixel size.

#### Quantification of *sams-3* splicing by RT-PCR

The total RNA was isolated from various sources: WT worms grown on various food sources, or transgenic worms with WT or MUT transgene reporter constructs or HeLa cells expressing worm reporter constructs. The RNA was reverse transcribed using Maxima H Minus First Strand cDNA (Thermo Fisher, K1682) with random primers. The cDNA was diluted to 50 μl, and of this a 2 μl aliquot was used for PCR. Primers used for the PCR were MM395 and MM396 in the case of endogenous *sams-*3 transcript or MM363 and MM364 in the case of transgene reporter constructs construct (Table S3). Transgene reporter constructs PCR amplification (MM363, MM364) generates three bands: intron-retained transcript (517 bp), alternatively spliced transcript (239 bp) and fully spliced isoform (148 bp), while for the endogenous *sams-3* transcript PCR generates: intron-retained transcript (502 bp), alternatively spliced transcript (225 bp) and fully spliced isoform (133 bp). The PCR products were resolved in a 2% agarose gel (Figures 3D, S3A, 5A, S4C, and S5B), stained with ethidium bromide and visualized under UV light in a gel visualization system (Vilber Lourmat E-Box VX2). Gel pictures were analyzed using Fiji software package (Schindelin et al., 2012). The intensity of each transcript isoform was calculated using the gel analyzer function. It was then normalized to the total intensity of all the transcript isoforms within one sample, allowing for internal normalization and comparison of different samples. Results obtained by RT-PCR quantification of worms grown on different food sources are in agreement with RNA-seq quantification.

#### Quantification of RNA modifications using LC-MS/MS

Total RNA was isolated by Trizol extraction from adult *C. elegans* (worm), the *Bombyx mori* (Silkmoth) BmN4 cell line (insect) and adult mouse testes (mouse), as indicated in Figure 1B. These RNAs were also used to purify poly(A)^+^ RNA. The RNAs were hydrolyzed to ribonucleosides by 20 U Benzonase® Nuclease (Santa Cruz Biotech, cat. no. sc-202391) and 0.2 U Nuclease P1 (Sigma, cat. no. N8630-1VL) in 10 mM ammonium acetate pH 6.0 and 1 mM magnesium chloride at 40°C for 1 h. After that, ammonium bicarbonate to 50 mM, 0.002 U phosphodiesterase I and 0.1 U alkaline phosphatase (Sigma) were added, and incubated further at 37°C for 1 h. The hydrolysates were mixed with 3 volumes of acetonitrile and centrifuged (16,000 x g, 30 min, 4°C). The supernatants were dried and dissolved in 50 μL water for LC-MS/MS analysis of modified and unmodified ribonucleosides. Chromatographic separation was performed using an Agilent 1290 Infinity II UHPLC system with an ZORBAX RRHD Eclipse Plus C18 150 × 2.1 mm ID (1.8 μm) column protected with an ZORBAX RRHD Eclipse Plus C18 5 × 2.1 mm ID (1.8 μm) guard column (Agilent). The mobile phase consisted of water and methanol (both added 0.1% formic acid) run at 0.23 mL/min. For modifications, starting with 5% methanol for 0.5 min followed by a 2.5 min gradient of 5%–15% methanol, a 3 min gradient of 15%–95% methanol and 4 min re-equilibration with 5% methanol. A portion of each sample was diluted for the analysis of unmodified ribonucleosides which was chromatographed isocratically with 20% methanol. Mass spectrometric detection was performed using an Agilent 6495 Triple Quadrupole system, monitoring the mass transitions 268.1-136.1 (A), 284.1-152.1 (G), 244.1-112.1 (C), 245.1-113.1 (U), 296.1-150.1 (m^6^Am), 282.1-150.1 (m^6^A and m^1^A), 282.1-136.1 (Am), 296.1-164.1 (m^6^_2_A), 283.1-151.1 (m^1^I), 298.1-166.1 (m^7^G), 312.1-180.1 (m^2,7^G), 326.1-194.1 (m^2,2,7^G), 258.1-126.1 (m^3^C and m^5^C), 274.1-142.1 (hm^5^C), 286.1-154.1 (ac^4^C), 259.1-139.1 (m1Ψ), 333.1-201.1 (5-methoxycarbonylmethyl-2-thiouridine, mcm5s2U), and 333.1-183.1 ((*S*)- and (*R*)-5-methoxycarbonylhydroxymethyluridine, S-mchm5U and R-mchm5U) in positive electrospray ionization mode, and 267.1-135.1 (inosine, I) and 243.1-153.1 (pseudouridine, Ψ) in negative electrospray ionization mode. Modifications detected in a mock control (containing only the hydrolytic enzymes) were subtracted from modifications detected in the RNA samples. In general, the mock control contained at least 1000-fold less RNA than the RNA samples and gave negligible background in the modification analyses.

#### Metabolomics analyses of worm lysates

Approximately, 3-4 L4 hermaphrodite worms were placed on 6 cm plates containing either nutrient-low media (NGM medium and seeded with OP50 bacteria) or nutrient-high media (peptone-rich medium and seeded with NA22 bacteria) (Table S4). After around 5 days of incubation at 20°C, when the progeny reached adult stage, the worms were washed off the plates with PBS, washed 2 more times with PBS and once with water. The excess of the liquid was discarded and packed worm pellet was immediately snap-frozen in liquid nitrogen and stored at −80°C. Then we shipped the lysates in dry ice to the Metabolomics Platform, Faculty of Biology and Medicine, University of Lausanne, Switzerland. Analysis was conducted as previously described for polyamines concentration measurement (Chevalier et al., 2020).

#### *In vitro* RNA methylation assay with human METTL16 and worm METT-10

Methylation assays were carried using chemically synthetized RNA oligos (IDT, Belgium or Microsynth, Switzerland) (Table S3). Recombinant untagged full-length human METTL16 or full-length *C. elegans* METT-10 were used (Figure 3B).

Before the experiment, RNA was refolded by heating the 100 μM RNA solution in H_2_O or 50 mM NaCl to 65°C in a Thermoblock (Eppendorf) for 5 min, and allowed to slowly cool down to the room temperature. All methylation reactions were performed in a 50 mM Tris-HCl, pH 7.5, 100 mM KCl, 5 mM MgCl2, 2 mM DTT buffer with 10 μM of refolded single-stranded RNA, 5 μg of recombinant protein, 1 μl of RiboLock RNase Inhibitor (Thermo Fisher, cat. no. EO0381) and 0.02 μCi of ^14^C-SAM (Perkin Elmer, NEC363010UC) in a total volume of 20 μl. Unless otherwise indicated, all reactions were performed overnight at room temperature. RNA was subsequently extracted using phenol/chloroform extraction protocol, resuspended in 15 μl of 2x RNA loading buffer (90% formamide, 0.02% SDS, 1 mM EDTA, 0.02% bromophenol blue, 0.02% xylene cyanol), heated for 5 min. at 70°C, cooled down to room temperature and loaded on a 15% Urea-PAGE gel.

The 15% Urea-PAGE gel was prepared by mixing 12.6 g of urea, 3 mL of 10x TBE (1 M Tris base, 1 M boric acid, 0.02 M EDTA), 11.25 mL of 40% acrylamide (19:1) and 6.75 mL of H2O. To catalyze gel polymerization, 240 μl of APS and 24 μl of TEMED (Merck, cat. no. 1107320100) were added. Gel was left to polymerize for 40 min. at room temperature. Wells were washed with 1xTBE to remove urea deposits and gel was pre-run in 1x TBE at 20 W for 25 min. RNA markers 5ʹ end-labeled with ^32^P-γ-ATP and composed of four single-stranded RNA oligos (RP_RNA_19: 40 nt, RP_RNA_1: 30 nt, RP_RNA_3: 28 nt, RP_RNA_18: 16 nt; Table S3) were loaded into the gel, together with 10 μl of RNA samples from the *in vitro* methylation assay. Gel was run at 12 W for 1 h 30 min.

After running, to visualize RNA bands, the gel was stained with a methylene blue solution [0.2% (w/v) methylene blue in a 1:1 solution of 0.4M sodium acetate and 0.4M acetic acid] for 10 min. This staining can be done by carefully sealing the gel in a plastic bag and shaking it on a rocking platform/frequently mixing the contents. Next, the gel was destained with 1xTBE and scanned using Epson Perfection 3200 Photo scanner. The destaining is also done by removing the staining solution, injecting the wash buffer into the bag and carefully mixing the contents. After scanning, the gel was dried in a gel dryer (Bio-Rad, model 583) with a gradual heating program, 80°C for 1.5 h. The dried gel was transferred to a cassette and exposed with a phosphor screen BAS (GE Healthcare) for 24 h. The phosphor screen was scanned in a Typhoon FLA 9500 laser scanner (GE Healthcare) at 700V and at 100 μm pixel size. Control software used for Typhoon FLA 9500 is the 1.1 version. Scans were analyzed using ImageQuant TL 8.1 software (GE Healthcare).

#### Preparation of RNA substrates for *in vitro* splicing assay

Both the human β-globin and adenovirus-based MINX splicing constructs were prepared by splint ligation (Moore and Sharp, 1992) of two RNA fragments. A longer T7 transcribed 5′ fragment that has 5′ exon and most of the intron, while a shorter 3′ synthetic RNA fragment (IDT, Belgium) that has the 3′ splice site and the 3′ exon. This allows introduction of either a methylated or unmethylated adenosine at the splice site by chemical synthesis.

To obtain the 5′ RNA fragment, both the β-globin and MINX DNA fragments were amplified by PCR. Forward primers contained T7 promoter sequence, while reverse primers contained 2ʹ-*O*-methyl residues at the last two nucleotides to prevent non-template nucleotide addition by the T7 polymerase. In addition, the MINX construct was mutated in order to create a version with a weaker polypyrimidine tract. The mutation was introduced by using a modified reverse primer (MM583) for the PCR reaction (Table S3). The PCR fragments were purified and used for T7 transcription with an m^7^G cap analog (m^7^G(5)ppp(5)G, NEB, Cat. No. S1404S) used in 4:1 ratio to GTP. Using m^7^G analog is essential as only capped transcripts are efficiently spliced. After the *in vitro* transcription, RNA was first purified with MicroSpin G-25 size exclusion columns (GE Healthcare, cat. no. 27-5325-01), and then it was extracted with phenol/chloroform.

The 3′ synthetic fragments without methylation, or with m^6^A at the 3′ splice site, or with m^6^A within the 3′ exon were purchased (IDT, Table S3). 50 pmol of the T7 transcribed RNA and 50 pmol of the synthetic RNA were mixed with 50 pmol of an antisense DNA oligo splint (that has extensive complementarity to the two RNA ends that need to be joined) in a total volume of 15 μl of an annealing buffer (10 mM Tris-HCl pH 7.5, 50 mM NaCl, 1 mM EDTA). The mix was heated in a thermocycler to 90°C and cooled down to 25°C (a program with gradual cooling at −0.1°C/second was used). Next, the RNA-DNA hybrid was incubated with 6μl of T4 DNA ligase mix (1x T4 ligase buffer, 1 μl of high-concentration T4 DNA ligase (2,000U/μl; NEB, cat.no. M0202M), 1 μl of RiboLock RNase Inhibitor, 2 μl PEG8000 (50%)) for 4 h at 37°C. The reaction was subsequently purified with phenol/chloroform and resuspended in 10 μl of H_2_O.

Approximately, 1 – 2 μl of ligated RNA was used for 3′ end labeling reaction, where it was mixed with 13 – 14 μl of pCp ligation mix(1 x T4 RNA Ligase 1 reation buffer, 1 mM ATP, 10% DMSO, 1 μl of T4 RNA Ligase 1 (NEB, cat.no. M0204), 1 μl of ^32^P-labeled cytidine 3′, 5′ bis(phosphate) (pCp, PerkinElmer, cat.no. NEG019A250UC), 1 μl of RiboLock RNase Inhibitor and water) and incubated overnight at 4°C. This reaction was then loaded on a 5% Urea-PAGE gel and the band corresponding to ∼200 nt ligated RNA was cut out, eluted and resuspended in 20 – 30 μl of H_2_O. This gives a splicing pre-mRNA substrate that is protected at the 5′ end with an m^7^G cap and radioactively marked at the 3′ end with ^32^P-labeled pCp.

#### Preparation of nuclear extracts

Splicing extracts were prepared as described before (Lee et al., 1988) using HeLa S3 cells. The cells were maintained at the concentration of 200 000 – 500 000 cells/mL and prior to collection were expanded to 600 000 – 800 000 cells/mL. It is important to harvest cells at the logarythimc growth stage. 400 mL of HeLa S3 suspension culture was collected (1200 x g, 5 min, 4°C), washed twice with 1xPBS and spun down (1200 x g, 5 min, 4°C) to assess packed cell volume (PCV). 300 mL of cell culture resulted in approximately 1 mL of PCV. All subsequent steps were performed on ice in a cold room (4°C). All buffers (placed in an ice bucket), pipette tips, Eppendorf tubes, etc. were pre-chilled in the cold room before use. Cells were gently resuspended in buffer A (10 mM HEPES-KOH, pH 7.9, 1.5 mM MgCl2, 10 mM KCl, 0.5 mM DTT) in a volume equal to PCV. After 15 min. cells were passed 6-times through a 23 gauge syringe (vigorous passage) and centrifuged at 12 000 x g for 20 s. at 4°C. The supernatant was removed and crude nuclear pellet was resuspended in buffer C (20 mM HEPES-KOH pH 7.9, 25% glycerol, 420 mM NaCl, 1.5 mM MgCl2, 0.2 mM EDTA, 0.5 mM PMSF, 0.5 mM DTT) in the volume of 2/3^rd^ of PCV. Resuspended nuclear pellet was mixed on a tube revolver (Thermo Scientific, cat. no. 88881002) with 15 rpm speed for 30 min. at 4°C. N ext, it was spun down at 12 000 x g for 5 min. at 4°C. Supernatant was transferred to a fresh tube and spun again (12 000 x g for 5 min. at 4°C), while the remaining pellet was removed. Supernatant (nuclear extract) was dialyzed twice for 2 hours in 100-times the volume of extract in buffer D (20 mM HEPES-KOH pH 7.9, 20% glycerol, 100 mM KCl, 0.2 mM EDTA, 0.5 mM PMSF, 0.5 mM DTT). Protease inhibitor PMSF was always added fresh, just before using the buffers. Extracts were flash frozen in liquid nitrogen and stored at −80°C. Quality of the lysates was verified by retrieving a frozen aliquot for use in a splicing assay.

#### *In vitro* splicing reaction

*In vitro* splicing reaction was done as described previously (Mayeda and Krainer, 1999) In short, 15 μl of nuclear extract was mixed on ice with 10 μl of a mix containing the RNA substrate [1 μl of RNA, 1.25 mM ATP, 10 mM creatine phosphate, 8 mM MgCl2, 50 mM HEPES-KOH, pH 7.3, 6.5% polyvinyl alcohol (PVA)] and incubated at 30°C for 0, 1 and 2 hours (for β-globin substrate) or 0, 15, 30 and 60 min. (for MINX substrate). At each time point, the reaction was stopped by adding 180 μl of splicing stop solution [0.3 M sodium acetate, pH 5.2, 0.1% (w/v) sodium dodecyl sulfate (SDS), 62.5 μg/mL tRNA (Sigma, cat. no. R-9001)] and kept at 4°C until all samples were ready. RNA was extracted with equal volume of water-saturated phenol (AppliChem; cat.no. A1624) and centrifuged for 10 min. at 12 000 x g at 4°C. Do not use chloroform at any point of the RNA extraction as it forms a very large interphase with PVA. After centrifugation, supernatant was transferred to a fresh tube and 1 μl of RNA grade glycogen (20 μg/μL, Thermo Scientific, cat.no. R0551) as well as 100% ethanol (2.5x the volume of supernatant) was added and RNA was precipitated for at least 1 hour at −20°C. The tubes were centrifuged at 14’000 x g for 30 min. at 4°C and RNA was resuspended in 15 μl of 2x RNA loading buffer (90% formamide, 0.02% SDS, 1 mM EDTA, 0.02% bromophenol blue, 0.02% xylene cyanol). 10 μl was loaded on 8% Urea-PAGE gel and run at 12W for 1 hour. ^32^P-labeled RiboRuler Low Range RNA Ladder (Thermo Scientific, cat. no. SM1831) was loaded as a molecular-weight size marker. Gel was dried and exposed with phosphor screen BAS (GE Healthcare). The phosphor screen was scanned in a Typhoon FLA 9500 laser scanner (GE Healthcare) at 700V and 100 μm pixel size.

#### Histology of mouse tissue sections

Adult males (post-natal day 60, P60) were euthanized using CO2 and testes were isolated. Pictures of freshly isolated testes were taken with SteREO Discovery V12 (Zeiss) (Figure 6C). To prepare the paraffin sections, isolated testes were fixed in Bouin’s solution (Sigma, cat. no. HT10132) for 48h at 4°C. Next, testes were washed in PBS for 48 h at 4°C, with frequent PBS changes. Samples were transferred into the embedding cassettes (Simport; cat. no. M508-3) and sent to the histology platform of University of Geneva. The samples were dehydrated in 70% (2 × 2h), 90% (1h), 95% (1h) and 100% ethanol (3 × 30 min) followed by incubation (3 times for 30 min) in HistoSAV2 (Biosystems). The solution was removed, replaced with paraffin, and incubated at 56-58°C. Testes were then transferred into plastic molds (Peel-A-Way® Embedding Mold S22, Polysciences, ; cat. no. 18646A-1) filled with paraffin and left at room temperature for paraffin to solidify. The sections (5 μm thickness) were cut using microtome (Leica RM2135) and mounted on the Superfrost Plus microscope slides (Thermo Fisher; cat no. 4951PLUS4). The sections were allowed to stretch for 24 h at 42°C and then were stored at room temperature.

Next, sections were stained using Hematoxylin and Eosin (H&E) stain protocol. The slides containing the paraffin sections were placed in a glass slide holder filled with HistoSAV2 (3 × 5 min) to remove the paraffin. For rehydration, the slides were incubated in 3x 100% ethanol, 96% ethanol, 70% ethanol, 50% ethanol and water (3 min for each step). Sections were stained with Hematoxylin solution (Merck) for 3-5 min and rinsed in running tap water. Then, sections were stained with Eosin Y solution (Sigma Aldrich; cat. no. E4382) for 3 to 5 min and washed with water. For dehydration, the sections were incubated in 50% (30 s), 70% (30 s), 96% (30 s), 100% ethanol (2 min) and HistoSAV (3 × 3 min). Neo-Mount (Merck) was put on the sections and immediately covered with coverslips. Pictures were taken using microscope AXIO Imager M2 (Zeiss).

#### Protein extraction from mouse tissues

Adult (P60) wild-type C57BL/6J male and female were euthanized using CO2 and various different tissues were isolated. Tissues were washed with PBS and immediately flash-frozen in liquid nitrogen. For lysate preparation, a piece the frozen tissue was cut out on a metal block placed on dry ice. The tissue piece was homogenized in 1 mL lysis buffer [50 mM Tris pH 7.4, 150 mM NaCl, 0.5% Triton X-100, 0.5% sodium deoxycholate, 1 mM DTT, Complete Protease Inhibitor Cocktail Tablet (Roche, Cat. No. 5056489001)]. The lysate was transferred to a 1.5 mL Eppendorf tube, centrifuged at 14000 x g for 30 min, and the supernatant was collected. Protein concentration was measured using the detergent-compatible colorimetric assay using a kit DC Protein Assay (Bio-Rad, 5000112). The reaction is similar to Lowry assay. The lysate concentration was normalized to 1 mg/mL using lysis buffer. Protein extracts were stored at −80°C.

#### Protein extraction from worms

Worms from three 6 cm plates containing synchronized adult population were washed off with M9 buffer. Worm pellets were washed additional 2 times, the excess of liquid was discarded and packed worm pellets were resuspended in lysis buffer (8 mM Na2HPO4, 2 mM KH2PO4, 137 mM NaCl, 100 mM KCl-1mM MgCl2, 1 mM EGTA, 10% Glycerol, 1% CHAPS, PMSF), 3x the volume of the worm pellet. The samples were immediately snap-frozen in liquid nitrogen and stored at −80°C. To obtain worm lysates, the samples were sonicated (10x 30 s ON, and 30 s OFF) with occasional snap-freezing in liquid nitrogen to break the worm cuticle. The lysates were clarified (20min spin 21’000 g at 4°C) and supernatant was transferred to fresh tubes. Protein concentration was measured immidiatelly after the protein extraction, using DC Protein Assay (Bio-Rad, 5000112). Protein lysate was mixed with 5X Laemmli Sample Buffer and water to a final concentration of μg/μl protein, boiled for 5 minutes at 95°C and frozen. Samples were stored at −80°C prior to the analysis.

#### Western blot

Mouse whole tissue lysates (30 μg/well) or worm lysates were separated on SDS-PAGE gels prepared using Ultra-Pure ProtoGel 30% acrylamide (37.5:1) (National Diagnostic; cat. no. EC-890) mixed with ultra-pure water and resolving gel buffer, to obtain 12% resolving gel (0.375 M Tris, 0.1% SDS, pH 8.8), or with stacking gel buffer to obtain 8% stacking gel (0.125 M Tris, 0.1% SDS, pH 6.8). N,N,N’,N’-Tetramethylethylendiamin (Merck, cat. no. 1107320100). The gel was polymerized by addition of 10% ammonium persulfate (AppliChem, cat.no. A1142). Gel electrophoresis was performed at 90 V for 30 min. and then at 120 V for 90 min. After separation, proteins were blotted on the Amersham Protran 0.45 mm nitrocellulose membrane (GE Healthcare; cat. no. 10600002) overnight at 5 V at room temperature using Trans-Blot SD. Semi-Dry Transfer Cell system (Bio-Rad; cat. no. 1703940). After transfer, membranes were washed with Tris-buffered saline (TBS, 20 mM Tris, 150 mM NaCl, pH 7.6) and blocked for 30 min. at room temperature with 5% dry milk in TBS with 0.05% Tween20 (TTBS) (SIGMA; cat. no. P7949). After 30 min. membranes were incubated with primary antibody: 1:500 anti-METTL16 (abcam; ab186012) or 1:100 anti-PARK7 (Invitrogen, PA5-13404) for 1 h at RT in 5% milk with TTBS. Membranes were then washed 5 times for 5 minutes with TTBS and incubated with HRP-conjugated anti-rabbit IgG HRP-linked (GE Healthcare; NA934) secondary antibody at 1:10,000 dilution for 1 h at RT in 5% milk in TTBS. After 1 h, membranes were washed 5 times for 5 minutes with TTBS followed by 3 washes for 5 minutes with TBS and incubated with one of the detection reagents: Amersham Prime Western Blotting Detection Reagent (GE Healthcare; RPN2232), SuperSignal West Femto Maximum Sensitivity Substrate (Thermo Fisher; cat. no. 34095) or Pierce ECL 2 Substrate (Thermo Fisher; cat. no. 1896433A) for 5 min. at room temperature. The chemiluminiscence signal was detected using Amersham Hyperfilm ECL (GE Healthcare; cat. no. 28906837). The processed films were scanned using Perfection 3200 Photo scanner (Epson) with XSane image scanning software (ver. 0999).

#### Preparation of RNA libraries

##### m^6^A-IP-seq to map m^6^A transcriptome-wide in mouse, worm and insects

To compare the extent of m^6^A RNA methylation between the species and map their location in the respective transcriptomes, we carried out m^6^A-IP-seq using pre-mixed RNAs from the different organisms. To this end, total or poly(A)^+^ RNA was isolated from the adult worm (*C. elegans*) grown on nutrient-high media (peptone-rich media+ NA22 *E. coli* strain, Table S4), adult mouse testis (P30) and Silkworm (*Bombyx mori*) BmN4-SID1 insect cell line. The RNAs were pre-mixed before further processing for RNA fragmentation and m^6^A-IP-seq. In this mixed sample, the mouse RNA serves as an internal control for efficient m^6^A immunoprecipitation via the unambiguous detection of m^6^A peaks that are already reported (Dominissini et al., 2012; Ke et al., 2015; Wojtas et al., 2017).

Poly(A)^+^ transcripts were purified from 75 μg of total RNA using the Dynabeads mRNA purification kit (Life Technologies; cat. no 61006). For total RNA fragmentation, 5 μg of total RNA each from mouse testis, adult worms and BmN4-SID1 cells was mixed with 2 μl of fragmentation reagent (AM8740, Thermo Fisher Scientific) in a final volume of 20 μl in a PCR tube. The reaction mix was incubated at 75°C for 12 minutes in a PCR machine. The tube was then transferred on ice immediately, and the reaction was stopped by adding 2.2 μl of stop solution provided with fragmentation reagent. Similarly, for poly(A)^+^ RNA fragmentation, 2 μg of poly(A)^+^ selected RNA each from mouse testis, adult worms and BmN4-SID1 was fragmented with fragmentation reagent as above. Denaturing urea-PAGE confirmed that majority of the RNA fragments were in the size range of 20-80 nts. A small portion (10%) of fragmented RNA from each sample was kept aside as input, while the remainder was subjected to immunoprecipitation.

The m^6^A immunoprecipitation was performed as described (Ke et al., 2015). Briefly, Protein A Dynabeads were washed once in PXL buffer (1 × PBS, 0.1% SDS, 0.5% sodium deoxycholate, 0.5% NP-40) followed by pre-treatment with BSA (final concentration 1 μg/ μL) in 200 μL PXL buffer for 45 minutes at RT. BSA pre-treated beads was then conjugated with m^6^A rabbit polyclonal antibody (20 μg; Synaptic Systems, catalog no. 202003) in 200 mL PXL buffer supplemented with 4 mL of RNasin RNase inhibitor (Promega; N2611) for one hour at RT on a rotating wheel. Dynabeads were further washed twice with PXL buffer, and finally, beads were resuspended in 400 mL of PXL buffer and 5 mL of RNasin. Fragmented RNA was added to the beads and incubated 4°C for 2 hours on a rotating wheel. After two hours incubation, the beads were washed twice by ice-cold Nelson low-salt buffer (15 mM Tris at pH 7.5, 5 mM EDTA), once by ice-cold Nelson high-salt buffer (15 mM Tris at pH 7.5, 5 mM EDTA, 2.5 mM EGTA, 1% Triton X-100, 1% sodium deoxycholate, 0.1% SDS, 1 M NaCl), once by ice-cold Nelson stringent wash buffer (15 mM Tris at pH 7.5, 5 mM EDTA, 2.5 mM EGTA, 1% Triton X-100, 1% sodium deoxycholate, 0.1% SDS, 120 mM NaCl, 25 mM KCl), and last by ice-cold NT-2 buffer (50 mM Tris at pH 7.4, 150 mM NaCl, 1 mM MgCl2, 0.05% NP-40). Antibody bound RNAs were eluted by incubating the beads with 0.5 mg/mL *N*^*6*^-methyl adenosine (Sigma-Aldrich; M2780) in NT2 buffer for one hour at 4°C. The eluted RNAs were precipitated with ethanol and glycogen and dissolved in RNase-free water. The input and IP RNAs were first 3ʹ end dephosphorylated with T4 PNK (NEB; M0201S, 10 U/mL) in the absence of ATP at 37°C for 45 minutes (40 mL reaction: 35.5 mL RNA, 4 mL 10X T4 PNK buffer, 0.5 mL of T4 PNK) followed by phosphorylation of 5ʹ end (50 mL reaction: 40 mL dephosphorylated RNA, 6.5 mL water, 1 mL RNasin, 0.5 mL 100 mM ATP, 1 mL 10X T4 PNK buffer 1 mL T4 PNK) at 37°C for 45 minutes. RNAs were phenol chloroform-extracted, ethanol precipitated and resuspended in 6 mL of RNase free water. The input RNA fragments and the immunopurified RNAs after the phosphorylation step were directly used for strand-specific library preparation (barcoded at 3′ end) using NEBNext® Multiplex Small RNA Library Prep Set for Illumina® (NEB; catalog No. E7560L) following manufacturer’s instructions. The libraries were resolved on 3% high-resolution MethaPhor agarose (Lonza; catalog. No. 50180) gels in 1X TAE buffer at 70 V. Fragments in the size-range of ∼150-250 bp were gel-extracted with the use of MinElute Gel Extraction Kit (QIAGEN; cat No. 28604). Multiple libraries with different barcodes (at 3′ end) were mixed in equimolar ratios and sequenced with the NextSeq Illumina® Platform (EMBL Gene Core facility, Heidelberg). The maximum sequencing length was 75 nucleotides. The list of sequencing libraries generated is provided in Table S1.

##### m^6^A-IP-seq to compare m^6^A levels in WT and mett-10 worms

For identification of m^6^A targets of METT-10 (Figures 1F and 1G), total RNA from biological triplicates of adult wild-type and *mett-10* KO worms, grown on nutrient-high media (Table S4), was isolated using TRIzol (Thermo Fisher Scientific) according to manufacturer instructions. Poly(A)^+^ transcripts were purified from 75 μg of total RNA using the Dynabeads mRNA purification kit (Life Technologies; cat. no 61006). The RNA fragmentation and m^6^A-IP-seq protocol, as described above, was followed for total RNA from WT, and poly(A)^+^ RNA from both WT and KO (in biological triplicates). For comparison between WT and the *mett-10* KO was made under different diet conditions (Figure 4B), the animals were fed on either nutrient-high or nutrient-low media (Table S4). Triplicate biological replicates were processed for m^6^A-IP-seq using poly(A)^+^ RNA. Library preparation was as described above.

### Quantification and statistical analysis

All statistical methods are indicated in the figure legends.

#### Analysis of m^6^A-IP-seq to compare m^6^A levels in **mouse, worm and insects**

The reads were sorted into individual libraries based on the barcodes and clipped using cutadapt (parameters: -a AGATCGGAAGAGCACACGTCT -m 15 -e 0.2 -O 4 -q 10–match-read-wildcards). The clipped reads were aligned to the mouse (GRCm38 – Ensembl release 95) or worm (WBcel235 – Ensembl release 95) genome using STAR (parameters:–outFilterType BySJout–limitOutSJcollapsed 50000000–limitIObufferSize 1500000000). *Bombyx* m^6^A distribution was not analyzed. We detected similar ratio of m^6^A/input reads for both species (Figure S1B). The m^6^A peak calling was done separately for reads mapped to mouse or worm genome using MACS2 (macs2 callpeak -f BAM -q 0.01–nomodel–extsize 50–call-summits). Consensus peaks from the biological replicates were identified using MSPC (parameters: -r bio -w 1e-4 -s 1e-8). Unlike in the mouse, only very low number of m^6^A peaks was identified in the worm. Peaks were annotated based on their overlap with the annotated features described in Ensembl gtf files (Figures S1C and S1D) and the enriched motifs were searched using MEME (parameters: -brief 50000 -nmotifs 5 -dna -revcomp -mod zoops -oc). Top motif is shown in Figures 1D and S1E. To investigate the m^6^A distribution along the individual transcripts, the adaptor trimmed reads from poly(A)^+^ libraries were mapped to individual ENSEMBL mRNAs using bowtie (parameters: -v 0 -a–best –strata). The read counts were divided by number of transcripts they mapped to and coverage was calculated using IRanges::coverage function along the transcripts. We focused only on transcripts from genes which showed significant (adjusted p value ≤ 0.1) increase in m^6^A/input ratio. These were identified using mapping the reads using SALMON (parameters: -l A -p 10–gcBias–validateMappings) followed by DESeq2 analysis. For the transcripts of these genes metaplots were created comparing the coverage along the 5′ UTRs, CDS and 3′ UTRs longer than 100 nt. Each part was divided into 100 pieces for which the mean coverage was calculated. In the mouse we observed expected m^6^A enrichment at the start of 5′ UTRs (due to m^6^Am) and at the end of CDS (Figure 1E). The worm distribution was rather uniform. The mean m^6^A enrichment was also plotted for 0.5 kb vicinity of the STOP codon, which also showed m^6^A enrichment only for the mouse.

#### Analysis of m^6^A-IP-seq comparing m^6^A levels in WT and *mett-10* KO worms

The reads were sorted into individual libraries based on the barcodes and clipped using cutadapt (parameters: -a AGATCGGAAGAGCACACGTCT -m 15 -e 0.2 -O 4 -q 10–match-read-wildcards). The clipped reads were aligned to worm transcripts (WBcel235 – Ensembl release 95) using bowtie (parameters: -v 0 -a -k 10–allow-contain). The read counts were divided by number of transcripts they mapped to and the counts were summarized to gene levels. To find the genes whose transcripts lose m^6^A methylation in the absence of METT-10 we looked for genes with significant (padj ≤ 0.1) decrease of m^6^A-IP/input ratio in *mett-10* KO using DESeq2 likelihood ratio test (LRT) where we compared the full model (∼input_or_m^6^A_IP + genotype + input_or_m^6^A_IP:genotype) to the reduced model (∼input_or_m^6^A_IP + genotype) (Figure 1G). We identified the transcripts of U6 snRNA genes and three highly similar SAM-synthetase genes (*sams-3*, *sams-4*, *sams-5*) as the main targets of METT-10. To identify the precise adenosine which is methylated in U6 snRNA transcripts, we mapped the reads to the worm consensus sequence of U6 snRNA (Figure S1G), which was obtained by BLAST of mouse *Rnu6* to worm genome, using bowtie (parameters: -v 0 -a -k 10–allow-contain) and plotted the normalized (reads per million - rpm) coverage which confirmed that the worm METT-10 recognizes the same motif as the human METTL16. The methylation was completely gone in the *mett-10* KO (Figure 1I). Plotting the read counts normalized to library sizes (rpm) showed that although the U6 snRNAs are not polyadenylated we still were able to obtain enough reads in the poly(A)^+^ libraries – but less than in the total RNA library from WT (Figures 1H and S1I). Comparison of normalized (rpm) read counts in input samples discovered a bit higher U6 snRNA levels in the KO (Figure S1H). To find out whether the loss of U6 snRNA m^6^A methylation in the KO affects general splicing, we aligned the reads to the genome (WBcel235 – Ensembl release 95) using STAR (parametres:–outFilterType BySJout –limitBAMsortRAM = 40000000000–outSAMattributes All) and used the STAR generated SJ.out.tab files to count the reads spanning the annotated and novel splice junctions. The read counts were normalized to library sizes (rpm). We did not observe any decrease in the input KO samples which would suggest the negative impact on splicing (Figure S1J).

Investigation of normalized (rpm) m^6^A coverage along SAM synthetase genes (*sams-3, sams-4, sams-5*), which showed decreased m^6^A/input ratio in the KO, revealed the m^6^A peak in the WT which overlaps the exon-intron boundary and is completely gone in the *mett-10* KO (Figure S2A). Comparison of DESeq2 normalized read counts in input samples showed increased expression of these genes (Figure S1, Figure S2E and S2C). Stronger WT m^6^A signal with clear peak summit was obtained from *sams-3* and *sams-4* which harbor identical sequence in this region. We used bowtie to align the reads specifically to this consensus sequence and plotted the normalized (rpm) coverage (Figure 2A), which identified the adenosine of the 3′ splice site to be methylated in the WT. Interestingly, when changing the plates on which the worms were grown from NA22 plates to OP50 plates, the methylation in the WT was strikingly decreased which was apparent from both the coverage along the exon-intron boundary and also the amount of reads mapping to the boundary (Figures 4B and S4A).

To visualize the read coverages along selected genomic loci, we calculated thee normalized read coverages (rpm) of STAR mapped reads for individual samples. Plotting of the normalized read coverage (rpm) of input samples along the *mett-10* locus demonstrated the loss of reads from the 5′ portion of *mett-10* in the KO (Figure S1F).

To investigate the effect of m^6^A loss on *sams-3* expression we plotted the mean coverage of its exons and introns from individual input samples (Figure 2B). This showed the general increased coverage of the exons in the KO, together with decreased coverage of intron 2 which contains the 3′ splice site methylated in the WT. To specifically compare the individual isoforms of *sams-3*: canonically spliced protein coding (PC), alternatively spliced non-coding (AS) and non-coding intron retained isoform (IR), following criteria were used. The PC abundance was estimated based on spliced read counts spanning the second intron (chrIV: 5848949-5849317), AS isoform was quantified based on the counts of spliced reads spanning alternative intron (chrIV: 5848949-5849224) and the IR variant abundance was calculated as mean coverage of the canonical intron minus 10 nucleotides from both sites. All counts were normalized to library sizes (rpm) (Figures 2C and S4B). Deep sequencing data generated during the study (Table S1) are deposited with the Gene Expression Omnibus (GEO: GSE146873).

#### Search for mouse genes with METTL16 methylation motif at their 3′ splice site

From 403563 annotated mouse 3′ splice sites in the ENSEMBL database, 916 were found to overlap with one of the METTL16 methylation motifs (UAC**m**^**6**^**AG**AGA or UAC**m**^**6**^**AG**AAA). Next, we extracted a 41 nt sequence centered around the target adenosine and predicted the secondary structure taken by the sequences using RNAfold. We then ranked the sequences based on the similarity of their predicted structures to that taken up by the bonafide METTL16/METT-10 target hairpin in the worm *sams-3* (same sequence as in RNA-1 in Table S3). The similarity score was calculated as a sum of nucleotide positions represented as “(” or “)” in the dot-bracket notation of the secondary structure in both test sequences and in RNA-1 sequence. Several of the top-ranked sequences were used for *in vitro* methylation assay with recombinant human METTL16 (Figures 6E and 6F).

To see whether any of the METTL16 motif-containing 3′ splice sites display increased usage in *Mettl16*^*−/−*^ mice, pointing toward their methylation dependent regulation, we searched our dataset (GEO: GSE116329) of E2.5 and E3.5 embryos (Mendel et al., 2018). We normalized the counts of splice junction reads spliced at the 3′ splice sites to gene expression levels, and searched for those 3′ splice sites with increased relative usage in *Mettl16*^*−/−*^. Two such sites with increased usage in E3.5 embryos were identified which localize to chr17:84777132-84777139 (in *Lrpprc* gene) and chr19:40350011-40350018 (in *Sorbs1* gene) (Figure 6G).

## Supplemental information

Document S1. Tables S1 and S3–S5

Table S2. List of genes with reduced m^6^A methylation in the worm *mett-10* MUT, related to Figure 1
