# Supplementary material for: Splice site m6A methylation prevents binding of U2AF35 to inhibit RNA splicing
Source: Cell. 2021 Jun 10;184(12):3125–3142.e25. doi: 10.1016/j.cell.2021.03.062 (PMC8208822; doi:10.1016/j.cell.2021.03.062)
Supplement: Document S1. Tables S1 and S3–S5 [file mmc1.pdf]

Cell, Volume *184*

Supplemental information

Splice site m^{6}A methylation prevents binding of U2AF35 to inhibit RNA splicing

Mateusz Mendel, Kamila Delaney, Radha Raman Pandey, Kuan-Ming Chen, Joanna M. Wenda, Cathrine Broberg Vågbø, Florian A. Steiner, David Homolka, and Ramesh S. Pillai

Table S1. List of all deep-sequencing libraries created in this study. Related to STAR Methods and Figures 1-4.

Data is available from Gene Expression Omnibus (GEO: GSE146873).

sample	experiment	description	reads	sequencing type
RR723	m6A profiling on RNA mix from mouse, worm and BmN4-SID1	INPUT-Total RNA-1	78748292	single-end
RR724	m6A profiling on RNA mix from mouse, worm and BmN4-SID1	INPUT-Total RNA-2	109227705	single-end
RR725	m6A profiling on RNA mix from mouse, worm and BmN4-SID1	INPUT-Total RNA-3	98509826	single-end
RR726	m6A profiling on RNA mix from mouse, worm and BmN4-SID1	INPUT-PolyA+ RNA-1	111319107	single-end
RR727	m6A profiling on RNA mix from mouse, worm and BmN4-SID1	INPUT-PolyA+ RNA-2	111369816	single-end
RR728	m6A profiling on RNA mix from mouse, worm and BmN4-SID1	INPUT-PolyA+ RNA-3	104925272	single-end
RR729	m6A profiling on RNA mix from mouse, worm and BmN4-SID1	m6A IP-Total RNA-1	97238279	single-end
RR730	m6A profiling on RNA mix from mouse, worm and BmN4-SID1	m6A IP-Total RNA-2	116365247	single-end
RR731	m6A profiling on RNA mix from mouse, worm and BmN4-SID1	m6A IP-Total RNA-3	103682866	single-end
RR732	m6A profiling on RNA mix from mouse, worm and BmN4-SID1	m6A IP-PolyA+ RNA-1	99423158	single-end
RR733	m6A profiling on RNA mix from mouse, worm and BmN4-SID1	m6A IP-PolyA+ RNA-2	90657183	single-end
RR734	m6A profiling on RNA mix from mouse, worm and BmN4-SID1	m6A IP-PolyA+ RNA-3	105299920	single-end
RR618	m6A profiling of the worm Mett-10 KO and WT on NA22 plates	input_totalRNA_WT-1	107952378	pair-end
RR619	m6A profiling of the worm Mett-10 KO and WT on NA22 plates	input_totalRNA_WT-2	104276535	pair-end
RR620	m6A profiling of the worm Mett-10 KO and WT on NA22 plates	input_totalRNA_WT-3	99043875	pair-end
RR621	m6A profiling of the worm Mett-10 KO and WT on NA22 plates	m6A_IP_totalRNA_WT-1	88177640	pair-end
RR622	m6A profiling of the worm Mett-10 KO and WT on NA22 plates	m6A_IP_totalRNA_WT-2	92603501	pair-end
RR623	m6A profiling of the worm Mett-10 KO and WT on NA22 plates	m6A_IP_totalRNA_WT-3	80301018	pair-end
RR624	m6A profiling of the worm Mett-10 KO and WT on NA22 plates	input_polyA_2_WT-1	51187060	pair-end
RR625	m6A profiling of the worm Mett-10 KO and WT on NA22 plates	input_polyA_2_WT-2	52291052	pair-end
RR626	m6A profiling of the worm Mett-10 KO and WT on NA22 plates	input_polyA_2_WT-3	33117898	pair-end
RR627	m6A profiling of the worm Mett-10 KO and WT on NA22 plates	input_polyA_2_KO-1	39691391	pair-end
RR628	m6A profiling of the worm Mett-10 KO and WT on NA22 plates	input_polyA_2_KO-2	35240657	pair-end
RR629	m6A profiling of the worm Mett-10 KO and WT on NA22 plates	input_polyA_2_KO-3	39991008	pair-end
RR630	m6A profiling of the worm Mett-10 KO and WT on NA22 plates	m6A_IP_polyA_2_WT-1	12022240	pair-end
RR631	m6A profiling of the worm Mett-10 KO and WT on NA22 plates	m6A_IP_polyA_2_WT-2	36551759	pair-end
RR632	m6A profiling of the worm Mett-10 KO and WT on NA22 plates	m6A_IP_polyA_2_WT-3	25683360	pair-end
RR633	m6A profiling of the worm Mett-10 KO and WT on NA22 plates	m6A_IP_polyA_2_KO-1	54841480	pair-end
RR634	m6A profiling of the worm Mett-10 KO and WT on NA22 plates	m6A_IP_polyA_2_KO-2	44893258	pair-end
RR635	m6A profiling of the worm Mett-10 KO and WT on NA22 plates	m6A_IP_polyA_2_KO-3	43057847	pair-end
RR600	m6A profiling of the worm Mett-10 KO and WT on NA22 plates	input_polyA_1_WT-1	22391643	pair-end
RR601	m6A profiling of the worm Mett-10 KO and WT on NA22 plates	input_polyA_1_WT-2	23096755	pair-end
RR602	m6A profiling of the worm Mett-10 KO and WT on NA22 plates	input_polyA_1_WT-3	19042425	pair-end
RR603	m6A profiling of the worm Mett-10 KO and WT on NA22 plates	m6A_IP_polyA_1_WT-1	18362889	pair-end
RR604	m6A profiling of the worm Mett-10 KO and WT on NA22 plates	m6A_IP_polyA_1_WT-2	18425770	pair-end
RR605	m6A profiling of the worm Mett-10 KO and WT on NA22 plates	m6A_IP_polyA_1_WT-3	18620691	pair-end
RR892	m6A profiling of the worm Mett-10 KO and WT on OP50 plates	input_polyA_WT-1	44602975	single-end
RR893	m6A profiling of the worm Mett-10 KO and WT on OP50 plates	input_polyA_WT-2	57428958	single-end
RR894	m6A profiling of the worm Mett-10 KO and WT on OP50 plates	input_polyA_WT-3	63145019	single-end
RR895	m6A profiling of the worm Mett-10 KO and WT on OP50 plates	input_polyA_KO-1	56220445	single-end
RR896	m6A profiling of the worm Mett-10 KO and WT on OP50 plates	input_polyA_KO-2	46443279	single-end
RR897	m6A profiling of the worm Mett-10 KO and WT on OP50 plates	input_polyA_KO-3	42188533	single-end
RR898	m6A profiling of the worm Mett-10 KO and WT on OP50 plates	m6A_IP_polyA_WT-1	11536036	single-end
RR899	m6A profiling of the worm Mett-10 KO and WT on OP50 plates	m6A_IP_polyA_WT-2	11339449	single-end
RR900	m6A profiling of the worm Mett-10 KO and WT on OP50 plates	m6A_IP_polyA_WT-3	11065165	single-end
RR901	m6A profiling of the worm Mett-10 KO and WT on OP50 plates	m6A_IP_polyA_KO-1	14331371	single-end
RR902	m6A profiling of the worm Mett-10 KO and WT on OP50 plates	m6A_IP_polyA_KO-2	13228517	single-end
RR903	m6A profiling of the worm Mett-10 KO and WT on OP50 plates	m6A_IP_polyA_KO-3	12318564	single-end

Table S3. DNA primers and RNA oligonucleotides used in this study. Related to STAR Methods and Figures 1-6.

Name	Sequence	Name in the study/Comments
**Worm mutants**		
oKDMM42	GGTGATTGTTGTTCGAGTGC	Reverse primer for detecting and sequencing of intron 2 deletion in *sams-3*. If edit is present, it creates 473 bp with oKDMM43
oKDMM43	GTGACCAGGTAAGTAACCAACG	Forward primer for detecting and sequencing of intron 2 deletion in *sams-3*. If edit is present, it creates 473 bp with oKDMM42
oKDMM41	GACTCCAGCAGCGATTTCAG	Forward primer for detecting and sequencing of intron 2 deletion in *sams-4*. If edit is present, it creates 433 bp with oKDMM44
oKDMM44	ACGTTATCTCCGAAGCGTAG	Reverse primer for detecting and sequencing of intron 2 deletion in *sams-4*. If edit is present, it creates 433 bp with oKDMM41
oKDMM12	GCAACGTGTCGCTAAAGAGG	Forward primer for detecting and sequencing of intron 2 deletion in *sams-5*. If edit is present, it creates 526 bp with oKDMM39
oKDMM39	TCCGTCTGCTTACCTTTCGAG	Reverse primer for detecting and sequencing of intron 2 deletion in *sams-5*. If edit is present, it ceeates 526 bp with oKDMM12
oKDMM17	CCGTCATTCCCAAACTCCATC	Forward primer to detect and sequence C-terminal HA tag in *sams-3*. If edit is present, it creates 554 bp band with oKDMM17
oKDMM18	ATGAACCAACCGCTGAGAAC	Reverse primer to detect and sequence C-terminal HA tag in *sams-3*. If edit is present, it creates 554 bp band oKDMM17 to be sequenced
oFSa0131	TGTGGTGTTTGCACAGGTAG	Forward primer to detect and sequence deletion of *mett-10*. If edit is present, it creates around 260 bp band with oFSa0195
oFSa0195	ATGTCCATGCCAACATCCAGC	Reverse primer to detect and sequence deletion of *mett-10*. If edit is present, it creates around 260 bp band with oFSa0131
oKDMM5	AAACTTGATTGTACAATGTCTGAG	sgRNAs for C-terminal HA tagging of *sams-3*
oKDMM6	TCTTGTCAGACATTGTACAATCAA	
oKDMM7	CGATCTCAAACGGAAATGGAATTGCTCATGGAGGAGGATCAATGGGAGGATACCCATATGAT<br>GTTCCAGATTACGCTGGCAGTTACCCATATGATGTTCCAGATTACGCTGGCAGTTAATGATT<br>GTACAATGTCTGATTCATTTGTTG	Repair template for the C-terminal tagging of *sams-3*
oKDMM19	AAACTTTCTGTAATCAAAGTAACC	sgRNA for removing intron 2 of *sams-3*, *sams-4* and *sams-5*
oKDMM20	TCTTGGTTACTTTGATTACAGAAA	
oKDMM21	AAACTTGTAACAGGTAAAGAAACC	sgRNA for removing intron 2 of *sams-3*, *sams-4* and *sams-5*
oKDMM22	TCTTGGTTTCTTTACCTGTTACAA	
oKDMM23	AAACCCTGAATCACTCACCACAAGCAC	sgRNA for removing intron 2 of *sams-4*
oKDMM24	TCTTGTGCTTGTGGTGAGTGATTCAGG	
oKDMM25	AAACCAAGCAACCTTAGCGTGAGC	sgRNA for removing intron 2 of *sams-4*
oKDMM26	TCTTGCTCACGCTAAGGTTGCTTG	
oKDMM27	TCTTGCTCACGCAAAGGTTGCATG	sgRNA for removing intron 2 of *sams-3*
oKDMM28	AAACCATGCAACCTTTGCGTGAG	
oKDMM29	TCTTGAAAGGTTGCATGTGGTAAG	sgRNA for removing intron 2 of *sams-3*
oKDMM30	AAACCTTACCACATGCAACCTTTC	
oKDMM31	AAACACAGTTTCTGTAAAACTTAC	sgRNA for removing intron 2 of *sams-5*
oKDMM32	TCTTGTAAGTTTTACAGAAACTGT	
oKDMM33	AAACCAAGCAACCTTGGCGTGAGC	sgRNA for removing intron 2 of *sams-5*
oKDMM34	TCTTGCTCACGCCAAGGTTGCTTG	
oKDMM35	CGACAGCTTTCGAGGTGATCTCACCGCACAACATGATCATACCCGTTTTAGTCACtGTTTCA<br>CAAGCAACCTTAGCGTGAGGGTCTTGAGCCAGATGAGCG	Repair template for removing intron 2 in *sams-4*
oKDMM36	CGCTGTTCTTGATGCTCATTTGGCTCAAGATCCTCACGCAAAGGTTGCATGTGAAACAGTGA<br>CTAAAACGGGTATGATCATGTTGTGCGGTGAGATCACCTC	Repair template for removing intron 2 in *sams-3*
oKDMM37	CAGCTTTTGAGGTGATCTCACCGCACAACATGATCATACCCGTTTTGGTCACTGTTTCACAA<br>GCAACCTTGGCGTGAGGATCCTGAGCCAAGTGAGCATC	Repair template for rem+A22:C38oving intron 2 in sams-5
**sams-3 artificial constructs**		
DH298	ACGAAGTGTTTCAACCTT	Forward primer for sams-3 FL amplification
DH299	CTCAATAAATGATTTTTTATAAG	Reverse primer for sams-3 FL amplification
DH300	CCCTAGAACACGGTCGTTCACATCAAGAACAGCGTCCGAGAT	Primer to introduce specific sequences into exon2
DH301	TGAACGACCGTGTTCTAGGGACGCAAAGGTTGCATGTG	Primer to introduce specific sequences into exon2
DH302	TACGCACTCAAAGTAGGCTGTCGGACGAGAACCTGGTAGTC	Primer to introduce specific sequences into exon3
DH303	ACAGCCTACTTTGAGTGCGTAATACGATGATTCCTCGAAAG	Primer to introduce specific sequences into exon3
DH304	CTTATAAAAAATCATTTATTGAGCCTGCAGGACGCTGGCATGC	Forward primer for pUC19 fragment amplification
DH305	AAGGTTGAAACACTTCGTTGTTGTTCTGGAAATTGAGA	Reverse primer for pUC19 fragment amplification
MM320	GATTACAGACTTGGTGACTAAAACGGGTATG	Forward primer for METT-10 consensus motif mutation
MM321	TTAGTCACCAAGTCTGTAATCAAAGTAACCG	Reverse primer for METT-10 consensus motif mutation
**HeLa splicing constructs**		
MM415	ATGGCTAGCCACCACGAAGTGTTTCAACCTTACAAC	Forward primer for sams-3 FL clonning into phRL-TK vector
MM416	CATGCGGCCGCTCTAGAACTCAATAAATGATTTTTTATAAGGTC	Forward primer for sams-3 FL clonning into phRL-TK vector
**Splicing**		
MM378	GGTGAACGTGGATGAAGTTGGTGGTGAGGCCCTGGGCAGGTTGGTATCAAGGTTACAAGACA<br>GGTTTAAGGAGACCAATAGAAACTGGGCATGTGGAGACAGAGAAGACTCTTGGCCTCGAGGT<br>TTCTGATAGGCACTGACTCTCTCTGCCTATTGGTCTATTTTCCCACCCTTAGGCTGCTGGTG<br>GTCTACCCTTGGACCCAGAGGTTCTTTGAGGATATCCGTACACCATCAGGGTACGAGCTAGC<br>CCATGGCGTACACCATCAGGGTACGACTAGTAGATCTCGTACACCATCAGGGTACGGAATTC	Human B-globin with T7 promoter
MM379	TAATACGACTCACTATAGGGTGAACGTGGATGAAGTTG	Forward primer to amplify 3' fragment of B-globin
MM460	/52MOErG//i2MOErG/GAAAATAGACCAATAGGCAG	Reverse primer to amplify 3' fragment of B-globin
MM_RNA_59	/5phos/ACCCTTAGGCTGCTGGTGGTCTACCCTTGGACCCAGAGGTTCTTTGAG	Unmethylated oligo for B-globin pre-mRNA ligation

MM_RNA_60	/5phos/ACCCTTm6AGGCTGCTGGTGGTCTACCCTTGGACCCAGAGGTTCTTTGAG	Splice site methylated oligo for B-globin pre-mRNA ligation
MM_RNA_61	/5phos/ACCCTTAGGCTGCTGGTGGTCTm6ACCCTTGGACCCAGAGGTTCTTTGAG	Exon methylated oligo for B-globin pre-mRNA ligation
MM453	CTGGGTCCAAGGGTAGACCACCAGCAGCCTAAGGGTGGGAAAATAGACCAATAGGCAGA	Splint oligo for B-globin ligation
MINX_FL	CCACTCTTGGATCGGAAACCCGTCGGCCTCCGAACGGTAAGAGCCTAGCATGTAGAACTGGT<br>TACCTGCAGCCCAAGCTTGCTGCACGTCTAGGGCGCAGTAGTCCAGGGTTTCCTTGATGATG<br>TCATACTTATCCTGTCCCTTTTTTTTCCACAGCTCGCGGTTGAGGACGACAAACTCTTCGCG<br>GTCTTTCCAGTGGGG	MINX pre-mRNA sequence
MM_RNA_85	/5phos/cacagctcgcggttgaggacaaactcttcgcggtctttccagtgg	Unmethylated oligo for MINX pre-mRNA ligation
MM_RNA_86	/5phos/cacm6Agctcgcggttgaggacaaactcttcgcggtctttccagtgg	Splice site methylated oligo for MINX pre-mRNA ligation
MM_RNA_87	/5phos/cacagctcgcggttgagg/iN6Me-rA/caaactcttcgcggtctttccagtgg	Exon methylated oligo for MINX pre-mRNA ligation
MM578	gagtttgtcctcaaccgcgagctgtggaaaaaaaaggg	Splint oligo for MINX ligation
MM617	TAATACGACTCACTATAGGGCCACTCTTGGATCGGAAACCC	Forward primer to amplify 3' fragment of MINX
MM582	/52MOErG//i2MOErA/aaaaaaagggacaggaTaagtatgac	Reverse primer to amplify 3' fragment of MINX
MM583	/52MOErG//i2MOErT/gtgttaaaagggacaggaTaagtatg	Reverse primer to amplify 3' fragment of the weak polypyrimidine tract MINX
MM585	gagtttgtcctcaaccgcgagctgtggtgtgttaaaaggg	Splint oligo for the weak polypyrimidine tract MINX ligation
***sams-3* RT-PCR**		
MM395	CTCGGACGCTGTTCTTGATG	PCR for intron retention, endogenous sams-3
MM396	AACCTGGTAGTCGACGACAG	PCR for intron retention, endogenous sams-3
MM363	TGTGAACGACCGTGTTCTAGG	PCR for intron retention in mini-gene constructs
MM364	CGTATTACGCACTCAAAGTAGGC	PCR for intron retention in mini-gene constructs
***Mettl16* knock-in mice**		
tracerRNA		tracerRNA, 67 bases, cat no 1072533, IDT
CD.Cas9.PGDV2419.AB, RNA	CTCAAAGGAACCACTCAGCG GUUUUAGAGCUAUGCU	gRNA for cat-dead and RNA-binding mutant *Mettl16* mouse
MM233	TCATCTCCCTAGTGGTGAAAGTGCCGCAGAAGACACTCCTGATGGATGCGCTTAAAGAAGAG<br>TCTGAGATAGTCTATGACTTCTGTATGTGCAACCCTCCCGGCTTCGCCAACCAATTGGAAGC<br>CAAGGTACAGTACCTTTAAAGTTACCAAATGCTTAGCTAGCTTTTACATCATTGCAGAGATA<br>TCAACAAAAGGCAG	ssDNA template for homology recombination for F187G modification
MM232	TCATCTCCCTAGTGGTGAAAGTGCCGCAGAAGACACTCCTGATGGATGCGCTTAAAGAAGAG<br>TCTGAGATAGTCTATGATTTCTGTATGTGCAACGCCGCATTTTTTGCCAACCAATTGGAAGC<br>CAAGGTACAGTACCTTTAAAGTTACCAAATGCTTAGCTAGCTTTTACATCATTGCAGAGATA<br>AGAACAAAAGGCAG	ssDNA template for homology recombination for 185AA-PP186 modification
MM342	TCTCCATTGCTGCAGGTCTG	Forward primer for F187G genotyping
MM343	CTTCCAATTGGTTGGCGAAGC	Reverse primer for F187G genotyping
MM348	CGTCTCCATTGCTGCAGGTC	Forward primer for PP185-186AA genotyping
MM349	ATTGGTTGGCAAAAAATGCGGC	Reverse primer for PP185-186AA genotyping
MM340	AACTTGATGCGTCTCCATTG	Forward primer to amplify the whole catalytic site region for sequencing
MM341	TGGTAACTTTAAAGGTACTGTACC	Reverse primer to amplify the whole catalytic site region for sequencing
**Mettl16 conditional KO**		
MM101	aaagatttgccttggcgtttcc	Forward primer to detect loxP insertion in the *Mettl16* loxus
MM102	cccaggaagtggcactatcaag	Reverese primer to detect loxP insertion in the *Mettl16* locus
oIMR7643	CACGTGCAGCCGTTTAAGCCGCGT	Forward primer for *Ddx4-cre* genotoping
oIMR7644	TTCCCATTCTAAACAACACCCTGAA	Reverse primer for *Ddx4-cre* genotyping
MM314	AAATTCAAATGGGCATTGGG	Forward primer for *Mettl16 deletion genotyping*
MM315	CCGAATCCTCATCTATTTACATTG	Reverse primer for *Mettl16 deletion genotyping*
**In vitro methylation**		**Name in the study/Comments**
MM RNA 5	CCCCCCCGUAGGCUACAGAGAAGGGGGGG	Mouse 3'UTR ; 29nt
MM RNA 6	UGUUGGCGUAGGCUACAGAGAAGCCUUCA	Mouse 3'UTR mut ; 29nt
MM RNA 31	ACAACGGUUACUUUGAUUACAGAAACGGUGACUAAAACGG	RNA-0; 40nt
MM RNA 32	GGUUACUUUGAUUACAGAAACGGUGACUAA	RNA-1 ; 30nt
MM RNA 34	GGUUACUUUGAUUACUGAAACGGUGACUAA	RNA-2 ; 30nt
MM RNA 33	GGUUACUUUGGUUACAGAAACGGUGACUAA	RNA-3 ; 30nt
MM RNA 35	GGUUACUUUGAUUACAGACUUGGUGACUAA	RNA-4 ; 30nt
MM RNA 36	GGUUACUUUGAUUACAGUAACGGUGACUAA	RNA-5 ; 30nt
MM RNA 37	GGUUACUUUGAUUACAGAGACUGUGACUAA	RNA-6 ; 30nt
MM RNA 43	GGUUACUUUGAUUACAGAGACGGUGACUAA	RNA-7 ; 30nt
MM RNA 38	CCCCCCCCUACAGAAACCCCCCCCC	RNA-8 ; 25nt
MM RNA 39	CCCCCCCCUACAGAAACGGGGGGGG	RNA-9 ; 25nt
MM RNA 100	GGUUACUUUGAUUACAGAAACUCAUUGGAA	RNA-10 ; 30nt
MM RNA 101	UCAGUGGUUGAUUACAGAAACUCAUUGGAA	RNA-11 ; 30nt
MM RNA 120	GGUUACUUUGAUUACAGAAACGGUGUGUAA	RNA-12 ; 30nt
MM RNA 121	GCAUACUUUGAUUACAGAAACGGUGUGUAA	RNA-13 ; 30nt
MM RNA 122	GGUUACUUUGAUUACAGAAACGCAGACUAA	RNA-14 ; 30nt
MM RNA 123	GCAUUGUUUGAUUACAGAAACGCAGUGUAA	RNA-15 ; 30nt
MM RNA 124	CCCCCCUUUGAUUACAGAAACGGGGGGGAA	RNA-16 ; 30nt
MM RNA 44	AAACUUUGUUUUAUUAGAAACUGUUGCUAA	Mouse ; 30nt
MM RNA 45	AAACUUUGUUUUAUUUGAAACUGUUGCUAA	Mouse mut ; 30nt
MM RNA 46	UUAUUUAUAUCCUACAGAAACCAUAACUAA	Silkworm ; 30nt
MM RNA 47	UUAUUUAUAUCCUACUGAAACCAUAACUAA	Silkworm mut ; 30nt
MM RNA 48	CAUAAUAUUUAAUACAGAAACCGUCGCCAA	Fly ; 30nt
MM RNA 49	CAUAAUAUUUAAUACUGAAACCGUCGCCAA	Fly mut ; 30nt
MM RNA 67	UAUUUUUUUCUUUCUCUUACAGAAAUGAAAAAGAAGAUGAA	RNA-67/Mucl1 ; 40nt
MM RNA 68	AGAGUGGUUUCUGUUUUUACAGAAAUGGAAACUGAAAAUAG	RNA-68/Spag1 ; 40nt
MM RNA 69	AAUCUCUAUGUAUUUCUUACAGAAAAUGCAUCAGAAACGUG	RNA-69/Pla2r1 ; 40nt
MM RNA 70	UUUCUGGAACUUAUUUUUACAGAAAAGUUUUAGAGACUGUC	RNA-70/Gyg ; 40nt
MM RNA 71	GCAUGGGGUGGGUCUAUUACAGAAACCAGCCCCGAGCAAAG	RNA-71/Des ; 40nt
MM RNA 72	AUGCAAAACUUUCUGUUUACAGAGAACCCCGGUUUCUCAUA	RNA-72/Helt ; 40nt
MM RNA 73	ACUCUUGUCUUCUGUCUUACAGAGAACGGAAGGCUCCCUUA	RNA-73/Pecam ; 40nt
MM RNA 74	AUUCUUUUUUUUUUCAUUACAGAGAAAGACAAAGCCUGAUC	RNA-74/Gm48535 ; 40nt
MM RNA 75	CUUUUGGGCUUUUUCAUUACAGAGAAAUUUGGAAGCACAUG	RNA-75/Gm15810 ; 40nt
MM RNA 76	GAUGCUUGUUUUGUGUUUACAGAGAAAAUGACAGGGUGGUC	RNA-76/Tjp2 ; 40nt
MM RNA 80	UAGGAUUUUUUUUCUUCUACAGAAACGACUGGGAGCCCCCAGAUA	Sorbs1 ; 45nt
MM RNA 81	UAAUUGUGUUUCAUUUUACAGAGAGCGUCUGAGCCAAGCC	Lrpprc ; 40nt

RP RNA 19	GCGUCUUUACGGUGCUUAAAACAAAACAAAACAAAACAAA	40 nt RNA marker
RP RNA 1	TGACATGAACACAGGTGCTCAGATAGCTTT	30 nt RNA marker
RP RNA 3	UGACAUGAACACAGGUGCUCAGAUAGCU	28 nt RNA marker
RP RNA 18	AGCACCGUAAAGACGC	16 nt RNA marker
**ITC**		
KM RNA 10	CUAGG	ITC experiment; 5 nt
KM RNA 11	CUm6AGG	ITC experiment; 5 nt
**SCARLET**		
MM437	GGAGAGACAACTTAAAGAGACTTAAAAGATTAATTTAAAATTTATCAAAAAGAGTATTGACT TAAAGTCTAACCTATAGGATACTTACAGCCATCGCCGGTCCTGTGAGTTAATAG	Universal DNA oligo for splint ligation
MM438	TGATCATACCCGTTTTAGTCACCGTTTCTCTATTAACTCACAGGACCGGCGATGGCTG	sams-3 splint oligo
MM698	mCmGmUmUmUmCmU GTAA mUmCmAmAmAmGmU	sams-3 chimera
MM614	CCTTGCGCAGGGGCCATGCTAATCTTCTCTCTATTAACTCACAGGACCGGCGATGGCTG	U6snRNA splint oligo
MM702	mCmUmUmCmUmCmU GTAT mUmGmUmUmCmC	U6 snRNA chimera
MM616	CCACTTTTTATATCTATAGCGCGCGTTGTCTATTAACTCACAGGACCGGCGATGGCTG	Positive control splint oligo
MM615	mGmCmGmUmUmGmU TCAG mAmGmUmCmUmAmA	Positive control chimera
MM_RNA_98	UUAGACUCUGAACAACGCGCGCUAUAGAUAUAAAAAGUGG	Positive control oligo - unmethylated
MM_RNA_99	UUAGACUCUGAm6ACAACGCGCGCUAUAGAUAUAAAAAGUGG	Positive control oligo - methylated
MM_RNA_96	GTTACAACGGTTACTTTGATTACAGAAACGGTGACTAAAACGGGT	sams-3 control oligo - unmethylated
MM_RNA_97	GTTACAACGGTTACTTTGATTACm6AGAAACGGTGACTAAAACGGGT	sams-3 control oligo - methylated

Table S4. Composition of nutrient-low and nutrient-high plates used for *C. elegans* culture. Related to Figure 1-4

Nutrient-low media

**NGM (nematode growth medium)**	**2 litres**
Agar	35 g
Peptone	5 g
Phosphate Buffer pH 6,0 + NaCl	50 ml phosphate buffer + NaCl
MgSO4 1 M	2 ml
CaCl2 1 M	2 ml
Cholesterol in ETOH (5 mg/ml)	2 ml
**Buffer NGM Phosphate + Nacl 40X**	**2 litres**
1 M Phosphate Buffer pH:6.0	72 g K2HPO4
	216 g KH2PO4
2 M NaCl	240 g NaCl

Nutrient-high media

**Pepton-rich plates**	**2 litres**
Agar	50 g
Peptone	40 g
Phosphate Buffer pH: 6,0 + NaCl	50 ml phosphate buffer + NaCl
MgSO4 1M	2 ml
Cholesterol in ETOH (5 mg/ml)	2 ml
**Buffer Pepton Rich Phosphate + Nacl 40X**	**2 litres**
1 M Phosphate Buffer	40 g K2HPO4
	240 g KH2PO4
820 mM NaCl	96 g NaCl

Table S5: List of worm strains used in this study. Related to Figure 1-4.

Strain	Genotype	Source	Comments
N2	wild type	CGC	wild type strain
VC1743	*mett-10 (ok2204) III*	CGC	*Mett-10* deletion
FAS198	*sams-3* (*uge125*) IV	This study	*sams-3* intron 2 deletion
FAS199	*sams-4* (*uge126*) IV	This study	*sams-4* intron 2 deletion
FAS200	*sams-3* (*uge127*) IV; *sams-4 (uge126)* IV	This study	*sams-3* intron 2 deletion and *sams-4* intron 2 deletion
FAS201	*sams-4* (*uge126*) IV; *sams-5 (uge128)* IV	This study	*sams-4* intron 2 deletion and *sams-5* intron 2 deletion
FAS202	*sams-3* (*uge129*) IV; *sams-4 (uge126)* IV; *sams-5 (uge128)* IV	This study	*sams3 i*ntron 2 deletion, *sams-4* intron 2 deletion and *sams-5* intron 2 deletion
FAS203	*sams-3* ((*uge130*) [*sams-3*::HA]) IV	This study	HA-tagged *sams-3*
FAS204	*sams-3 (uge131)* IV; *sams-3* ((*uge130*) [*sams-3*::HA]) IV	This study	HA-tagged *sams-3* with intron 2 deleted
FAS206	*sams-3* ((*uge130*) [*sams-3*::HA]) IV; *sams-1 ((uge132)* [OLLAS::*sams-1*]) X; *mett-10 (ok2204)* III	This study	HA-tagged *sams-3* with *mett-10* deletion<br>NOTE: This strain has *sams-1* tagged with OLLAS in the background, which was not used in this study
